# Mechanochemical solid state synthesis of copper(I)/NHC complexes with K_3_PO_4_

**DOI:** 10.3762/bjoc.19.34

**Published:** 2023-04-14

**Authors:** Ina Remy-Speckmann, Birte M Zimmermann, Mahadeb Gorai, Martin Lerch, Johannes F Teichert

**Affiliations:** 1 Institut für Chemie, Technische Universität Berlin, Straße des 17. Juni 115, 10623 Berlin, Germany; 2 Fakultät für Naturwissenschaften, Technische Universität Chemnitz, Straße der Nationen 62, 09111 Chemnitz, Germany

**Keywords:** ball mill, bifunctional catalysis, catalytic hydrogenations, copper, mechanochemical synthesis, N-heterocyclic carbenes

## Abstract

A protocol for the mechanochemical synthesis of copper(I)/N-heterocyclic carbene complexes using cheap and readily available K_3_PO_4_ as base has been developed. This method employing a ball mill is amenable to typical simple copper(I)/NHC complexes but also to a sophisticated copper(I)/N-heterocyclic carbene complex bearing a guanidine moiety. In this way, the present approach circumvents commonly employed silver(I) complexes which are associated with significant and undesired waste formation and the excessive use of solvents. The resulting bifunctional catalyst has been shown to be active in a variety of reduction/hydrogenation transformations employing dihydrogen as terminal reducing agent.

## Introduction

Prominent goals of green chemistry heralded for synthetic chemistry are minimization or ideally the complete prevention of chemical waste. In this vein, the use of innocuous chemicals, replacement of hazardous reagents, atom efficient reactions and overall safer chemical processes are desirable [1–2]. Therefore, one current challenge for syntheses is the development of green and environmentally friendly routes to access value-added products.

**Scheme 1:**
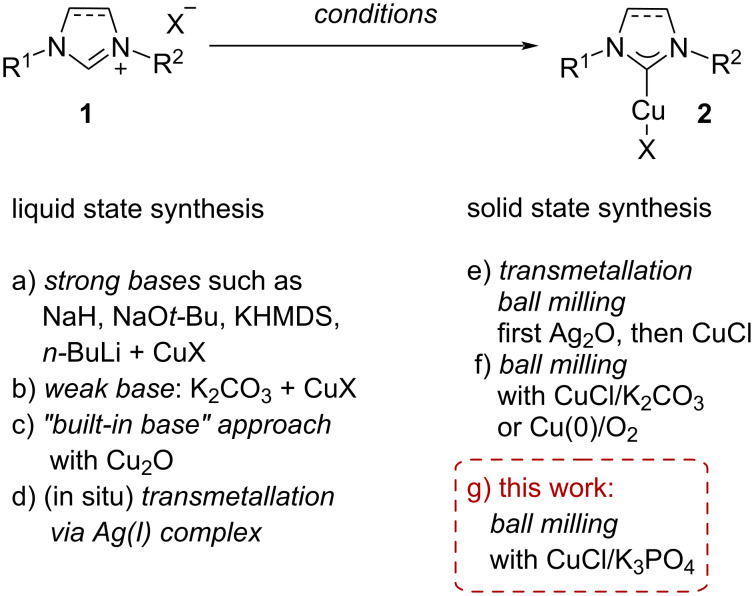
General synthetic routes to copper(I)/NHC complexes (X = Cl, Br).

One important way to more economical syntheses is the concept of catalysis to avoid stoichiometric amounts of reactants and to design reactions more atom efficient [1–3]. However, the focus has seldomly been on the preparative methods to access the required catalysts themselves. As case in point, we decided to re-investigate the synthesis of copper(I)/N-heterocyclic carbene (NHC) complexes, which are broadly applicable catalysts for a wide variety of transformations [4–6]. While generally there are many different synthetic routes to transition metal/NHC complexes [7–15] not all of them are applicable to the preparation of copper(I)/NHC compounds (Scheme 1) [5–6,13,16–19]. Generally, the so-called direct routes via the appropriate imidazol(in)ium salt, a copper precursor and a suitably strong base (e.g., NaH, NaO*t-*Bu, KHMDS or *n-*BuLi) [20–26] are challenging for copper complexes **2** as they tend to give low yields (Scheme 1a) [5–6,13,16–19]. One elegant protocol employing K_2_CO_3_ as weak base in combination with copper(I) salts for simple copper(I)/NHC complexes has been disclosed (Scheme 1b) [27]. While this variant is the method of choice due to its simplicity and practicability, other alternatives have to be sought in cases where these direct synthesis approaches fail: On the one hand, the so-called “built-in base” route relies on the use of Cu_2_O which can be directly reacted with a suitable NHC precursor **1** (Scheme 1c) [28]. In any case, the most common approach hinges upon the use of the preliminary preparation of an intermediate silver(I)/NHC complex followed by facile transmetallation to copper(I) (Scheme 1d). In some cases, this transmetallation step is carried out in situ [14–15,29–32]. Notably, these generally successful synthetic routes produce a considerable amount of transition metal waste (next to the inherent use of solvents) and are therefore in misalignment with the principles of green chemistry.

Syntheses via mechanochemical methods offer elegant and atom-economic alternatives to liquid state synthesis approaches [33–43]. In accordance with the in situ transmetallation route in liquid state synthesis, a one-pot two-step procedure in a ball mill was discovered (Scheme 1e) [44]. The possibility of synthesizing copper(I)/NHC complexes in the ball mill is promising due to the avoidance of organic solvents. Direct approaches from the NHC-precursor to the copper(I)/NHC complex not undergoing the transmetallation step have been disclosed (Scheme 1f) [45–47]. These approaches are an elegant alternative to the transmetallation route performing without unwanted transition metal waste. Two possible direct mechanochemical pathways have been presented for the synthesis of copper(I)/NHC complexes: First, the complexes can be synthesized by milling the ligand precursor **1** with metallic copper powder in air [45]. Another mechanochemical pathway was published using K_2_CO_3_ as a base and copper(I) chloride [46–47]. This latter procedure is practical, avoids the use of solvents, and relies on an abundant and cheap base.

**Scheme 2:**
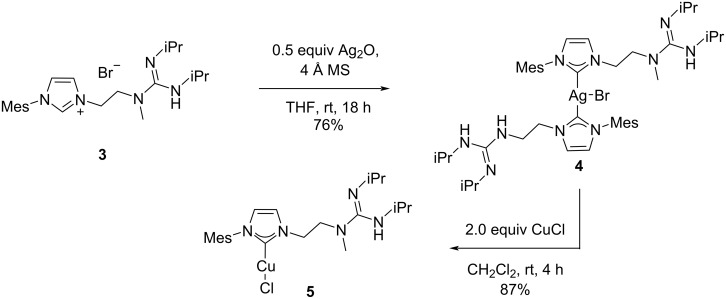
Preparation of sophisticated Cu(I)/NHC complexes: Synthesis of bifunctional catalyst **5** via transmetallation. Mes = mesityl [48–49].

We have recently disclosed an ester reduction with H_2_ as terminal reducing agent utilizing bifunctional copper(I)/NHC complex **5** bearing a guanidine moiety as additional catalytic unit [48]. This catalyst acts by employing the copper(I)/NHC complex for H_2_ activation on the one hand and by using the guanidine subunit for simultaneous organocatalytic activation of the ester on the other hand. Following a previously established synthetic pathway [49], we have found that transmetallation via silver(I)/NHC complex **4** was the only viable synthetic entry point to this sophisticated bifunctional catalyst (Scheme 2) [10,12,14,50]. First, the silver(I)/NHC complex **4** had to be synthesized and isolated prior to transmetallation with copper(I) chloride [48–49]. The required formation of silver(I) complex **4** diminishes the overall yield of copper complex **5**. As an additional disadvantage, the silver(I) byproducts have to be carefully removed in order to maintain reproducible results in subsequent catalytic hydrogenations [48]. We deemed this synthetic route unattractive with regards to sustainable synthesis due to the silver waste generated in the process and sought to replace the transmetallation route with a more atom economic approach to circumvent these problems.

## Results and Discussion

We therefore examined different approaches to avoid the transmetallation step (**4**→**5**) and to establish a protocol for the direct synthesis of **5** in solution from imidazolium salt **3** (Table 1, liquid state approaches) [48]. The use of strong bases such as *n-*BuLi or NaO*t-*Bu (in various equivalents, Table 1, entries 1 and 2) or weak bases (Et_3_N, K_2_CO_3_ or K_3_PO_4,_ Table 1, entries 3–5) either did give no conversion to **5** at all or delivered a catalytically inactive complex, which we assign to a CO_2_ adduct of **5** (Table 1, entry 4) [51–52]. We hypothesize that in this CO_2_ adduct, the guanidine moiety is unavailable to perform its assisting part in catalysis through hydrogen-bonding interaction [48]. As additional evidence to support the formation of the CO_2_ adduct of **5**, we can show that bubbling of CO_2_ through a solution of **5** leads to catalytically inactive complexes (see Supporting Information File 1 for details). This also supports the notion that during catalytic ester hydrogenation, the guanidinium moiety acts as a hydrogen bond donor to the esters [48]. The formation of a CO_2_ adduct hinders the ability to form hydrogen bonds. Furthermore, utilizing Cu_2_O for a “built-in base” approach did not give complex **5** (Table 1, entry 6).

**Table 1:** Attempted direct synthesis of bifunctional catalyst **5** from imidazolinium salt **3**: liquid and solid state approaches.

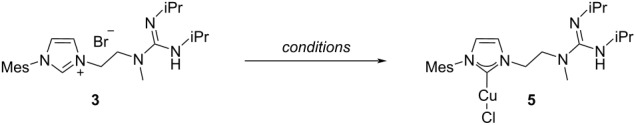				

Entry		Reagents	Conditions	Results

Liquid state aproaches [20]				

1	strong bases	1.00 equiv **3**, 1.10 equiv CuCl, 3.00 equiv *n-*BuLi	THF, 0 °C → rt, 16 h	no formation of **5**
2		1.00 equiv **3**, 1.10 equiv CuCl, 1.05/2.00/3.00/5.00 equiv NaO*t-*Bu	THF, rt, 16 h	no formation of **5**
3	weak bases	1.00 equiv **3**, 1.10 equiv CuCl, 3.00 equiv NEt_3_	THF, 0 °C → rt, 16 h	no formation of **5**
4		1.00 equiv **3**, 1.00 equiv CuCl, 2.00 equiv K_2_CO_3_	acetone, 60 °C, 16 h	formation of catalytically inactive **5**∙CO_2_
5		1.00 equiv **3**, 1.00 equiv CuCl, 2.00 equiv K_3_PO_4_	acetone, 60 °C, 16 h	no formation of **5**
6	“built-in base” approach	1.00 equiv **3**, 2.0 equiv Cu_2_O	CH_2_Cl_2_, 4 Å MS, 60 °C, 16 h	no formation of **5**

Solid state approaches (steel vessel (12 mL), 6 steel balls (1 cm diameter) if not noted otherwise)				

7	strong bases	1.0 equiv **3**, 1.0 equiv CuCl, 1.5 equiv NaO*t-*Bu	450 rpm, 4 h	no formation of **5**
8		1.0 equiv **3**, 1.0 equiv CuCl, 1.5 equiv NaOH	450 rpm, 4 h	no formation of **5**
9		1.0 equiv **3**, 1.0 equiv CuCl, 1.5 equiv KHMDS	450 rpm, 4 h	no formation of **5**
10		1.0 equiv **3**, 1.0 equiv CuCl, 1.5 equiv NaH	gastight zirconia vessel (45 mL), 6 zirconia balls (1.5 cm diameter), 450 rpm, 4 h	formation of **5** observed, inseparable mixture of products
11	no added base [46]	1.0 equiv **3**, 1.0 equiv CuCl	450 rpm, 4 h	formation of [**3**][CuClBr]^–^ observed by HRMS
12	“built-in base” approach	1.0 equiv **3**, 0.5 equiv Cu_2_O	450 rpm, 4 h	no formation of **5**
13	**weak base**	**1.0 equiv 3, 1.0 equiv CuCl, 1.5 equiv K** **_2_** **CO** **_3_**	**450 rpm, 4 h**	**44% of 5**
14		**1.0 equiv 3, 1.0 equiv CuCl, 1.5 equiv K** **_3_** **PO** **_4_**	**450 rpm, 4 h**	**91% of 5**

Since our attempts to establish direct synthetic routes to **5** from **3** in liquid state were not fruitful, we turned our attention to the mechanochemical synthesis of bifunctional catalyst **5**, based on two recent reports on preparation of copper(I)/NHC complexes [45,47].

All mechanochemical syntheses were carried out in a planetary ball mill and the vessel was loaded in an argon-filled glovebox. Copper(I) chloride, imidazolium salt **3** and the appropriate base were mixed (in a molar ratio of 1.0:1.0:1.5, respectively) and ground for 4 hours. Afterwards purification included dissolving the crude product in CH_2_Cl_2_, filtration over a PTFE syringe filter and concentrating the filtrate under reduced pressure. Employing strong bases such as KHMDS, NaO*t-*Bu or NaOH did not lead to the desired product (Table 1, entries 7–9). All three approaches have in common that the conjugated acid of the added base is a liquid. In the literature, the improvement of mechanochemical syntheses by addition of small amounts of a liquid have been reported (LAG, liquid-assisted grinding) [43]. However, in our case, the formation of small amounts of liquid during the milling process lead to agglutination of the remaining solids and therefore insufficient homogenization of the reaction mixture. This gave a mixture of compounds, in which the envisaged complex **5** could not be identified.

A different approach was made using sodium hydride as a base (Table 1, entry 10). Instead of small amounts of liquid, here, deprotonation leads to the formation of dihydrogen. Hence, another gastight mill was utilized for this approach. Unfortunately, a successful synthesis of **5** directly from **3** was not possible under these conditions: NMR analysis of the resulting mixture indicated the presence of **5**, but also of unwanted side-products that could not be identified. Purification of **5** from this complex mixture turned out not to be feasible. Further modifications of the milling conditions did not lead to the elimination of these side-products, therefore the experiments with NaH as a base were discontinued. As a side comment, the addition of no base at all led to the formation of the imidazolinium cuprate ([**3**][CuClBr]^−^, Table 1, entry 11) [46]. The direct transition of the “built-in base” approach conditions to mechanochemical synthesis (copper(I) oxide and imidazolium salt **3** as starting materials), lead to no formation of **5** (Table 1, entry 12).

The use of K_2_CO_3_ for the mechanochemical synthesis of copper(I)/NHC complexes [46–47] was feasible for the preparation of **5** (Table 1, entry 13). Importantly, we found that extending on this concept also K_3_PO_4_ could be employed equally well while giving significantly higher yields than the previous protocol [46–47] (Table 1, entry 14). All of the approaches discussed here are attractive due to the use of copper(I) chloride as the copper source. Interestingly, the use of K_2_CO_3_, which led to the formation of a catalytically inactive postulated CO_2_ adduct of **5** in the liquid state synthesis, did lead to catalytically active **5** in the mechanochemical approach. In a similar vein, the different outcome with K_3_PO_4_ as a base (which led to no catalytically active complexes in the liquid state synthesis) was surprising, as in the ball mill, clean and catalytically active copper(I) complex **5** was obtained. To avoid the possible formation of the catalytically inactive CO_2_ adduct when employing K_2_CO_3_ for the synthesis of **5** we decided to use K_3_PO_4_ for subsequent investigations (see also below, Table 2). Even though the imidazolium bromide salt **3** was employed in combination with CuCl as copper(I) precursor, elemental analysis of **5** clearly supported the formation of **5** as a chloride salt (see Supporting Information File 1).

**Table 2:** Synthesis of standard Cu(I)/NHC-complexes using K_3_PO_4_ as a weak base (standard procedure: steel vessel (12 mL), 6 steel balls (1 cm diameter), 450 rpm, 4 h). (dimer = [(NHC)_2_Cu^+^]Cl^−^).

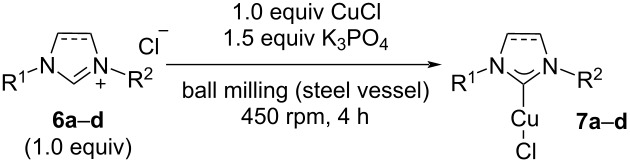			

Complex	Yield	Cu(0)/O_2_ [45]	CuCl/K_2_CO_3_ [46]

[Cu(IMes)Cl] (**7a**)	71% (8% homoleptic cationic Cu(I) complex)	85%	65%
[Cu(SIMes)Cl] (**7b**)	73%	76%	53%
[Cu(IPr)Cl] (**7c**)	63%	82%	78%
[Cu(SIPr)Cl] (**7d**)	64% (12% homoleptic cationic Cu(I) complex)	65%	66%

For the optimized protocol, the starting materials were mixed in a steel vessel and ground at 450 rpm for a total time of four hours. After ball milling, an off-white powder was obtained which gave complex **5** in very good yield of 91% after extraction with CH_2_Cl_2_ and filtration. NMR analysis of **5** matched previously reported data [48–49] and showed no side products. It has to be mentioned that complex **5** synthesized via the mechanochemical route is isolated as a CH_2_Cl_2_ adduct (**5**/CH_2_Cl_2_ = 1:1) as confirmed by NMR spectroscopy and elemental analysis. If complex **5** is formed via the liquid state synthesis [48–49], also a CH_2_Cl_2_ adduct is isolated, albeit with a **5**/CH_2_Cl_2_ ratio of 2:1.

In order to demonstrate the general applicability of the K_3_PO_4_-based protocol for the mechanochemical synthesis of copper(I)/NHC complexes, we decided to prepare the most common copper(I)/NHC complexes **7a**–**d** [5–6] employing our method (Table 2). When the corresponding imidazoli(ni)um salts **6a**–**d** were submitted to the standard protocol, complexes **7a**–**d** were obtained with acceptable yields, with similar yields compared to previous methods. In some cases, the homoleptic cationic copper(I) complexes [(NHC)_2_Cu]^+^CuCl_2_^−^ were observed as side products [48,53].

**Scheme 3:**
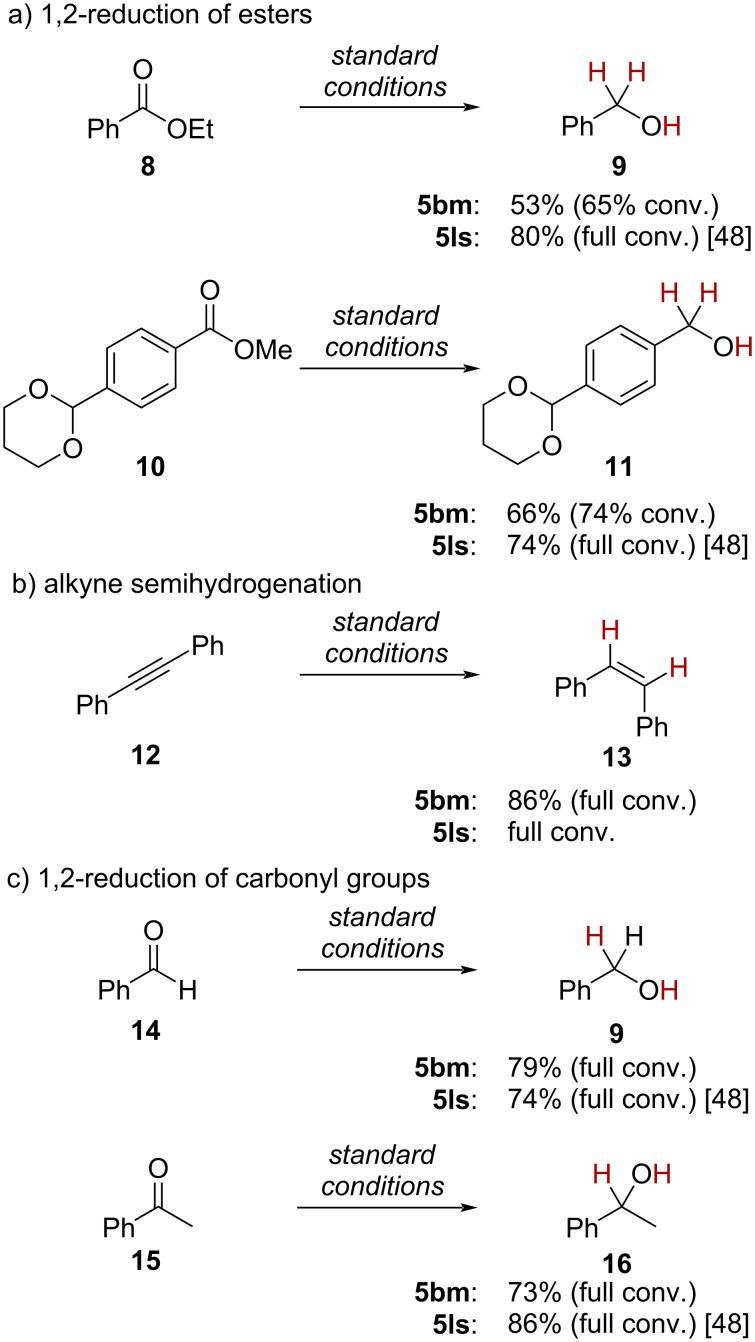
Application of bifunctional catalyst **5** in copper(I)-catalyzed hydrogenations: comparison of **5** prepared by solid state/ball milling (**5bm**) and liquid state (**5ls**) synthesis. Standard conditions: Substrate (0.40 mmol), 10 mol % **5**, 1.1 equiv NaO*t-*Bu, 1.3 equiv 15-crown-5, 100 bar H_2_, 1,4-dioxane (3 mL), 70 °C, 24 h.

We decided to directly compare complex **5** from mechanochemical synthesis (**5bm**) with its counterpart from the liquid state transmetallation route (**5ls**) in catalysis. We found that **5bm** was catalytically active, however displaying slightly diminished activity in general most likely due to different adduct ratio inhibiting the catalytic activity (Scheme 3). This was established using the standard reactions for catalytic hydrogenations with copper(I)/NHC complexes [4]. In this vein, we tested complex **5** from solid and liquid phase synthesis in the catalytic hydrogenation of esters, carbonyl compounds and in the semihydrogenation of alkynes. In the catalytic hydrogenation of ethyl benzoate (**8**) lower overall conversion to benzyl alcohol (**9**) and lower yield was found with **5bm** (65% conv. and 53% yield with **5bm**, in comparison to 100% conv. and 80% yield with **5ls**; Scheme 3a). We hypothesize that the higher amount of CH_2_Cl_2_ as part of the prepared complex, which is not a suitable solvent for catalytic ester reduction with H_2_ [48], led to lower catalyst activity. Possible coordination of residual phosphate to the guanidine moiety was excluded as analysis by ^31^P NMR experiments. The copper(I)-catalyzed 1,2-reduction of functionalized ester **10** was also successfully achieved using the ball mill synthesized bifunctional catalyst **5bm**, again with slightly diminished yields and conversions.

Application of the ball mill-synthesized complex **5bm** in the alkyne semihydrogenation of tolane (**12**) gave (*Z*)-stilbene (**13**) with full stereoselectivity in good yield (86%, Scheme 3b). Noteworthy, the complex **5** was never evaluated in this reported reaction. Therefore, **5bm** behaves similarly to other copper(I)/NHC complexes in this transformation [54–60]. The catalytic 1,2-reduction of carbonyl compounds is mainstay for copper(I)/NHC complexes [61–67], which is why we also tested **5bm** in these transformations: The 1,2-reduction of benzaldehyde (**14**) and acetophenone (**15**) proceeded with good yields (Scheme 3c). No aldol addition for the acetophenone substrate has been observed although working under strongly basic conditions [68–69].

## Conclusion

In conclusion, we have disclosed a practical approach to a sophisticated bifunctional copper(I)/NHC complex based on a mechanochemical protocol. This operationally simple synthetic route circumvents the previously necessary use of surplus transition metal reagents and therefore diminishes unwanted waste formation. The new protocol presented here is based on K_3_PO_4_ and has successfully displayed the activity of the resulting catalyst in a variety of hydrogenative transformations. We show that the new protocol is also amenable to the synthesis of other standard copper(I)/NHC complexes. Our results do not only add to the wide area of applications of mechanochemical synthesis but also showcase that transition metal complexes bearing additional functional groups can be prepared with a ball milling synthesis. We think that our protocol could be useful for the atom economic preparation of other complex catalysts, which are difficult or wasteful to be prepared by typical liquid state synthesis methods.

## Experimental

Mechanochemical synthesis procedure for **5**: The product was synthesized using a Fritsch Pulverisette 7 *classic line*, a high-energy planetary ball mill. The starting materials 1-(2-(2,3-diisopropyl-1-methylguanidino)ethyl)-3-mesityl-1*H*-imidazol-3-ium bromide (**3**, 75 mg, 0.16 mmol, 1.0 equiv), CuCl (17 mg, 0.16 mmol, 1.0 equiv) and K_3_PO_4_ (53 mg, 0.25 mmol, 1.5 equiv) were filled into a 12 mL steel vessel equipped with six steel balls (1 cm diameter). The beaker was sealed in an argon-filled glovebox. Milling was carried out with 450 rpm for a total of four hours. After each hour the milling was paused for 30 minutes to avoid overheating of the machine. The raw product was obtained as an off-white powder after milling. The ground product was mixed with CH_2_Cl_2_ (3 mL) and the resulting suspension was filtered over a PTFE syringe filter (0.45 μm). The filtrate was concentrated under reduced pressure. The product **5** was obtained as the CH_2_Cl_2_ adduct as a colourless solid (86 mg, 0.15 mmol, 91%).

## Supporting Information

File 1 General procedures, experimental details, analytical data and copies of NMR spectra.
