# Supplementary material for: Mechanochemical solid state synthesis of copper(I)/NHC complexes with K3PO4
Source: Beilstein J Org Chem. 2023 Apr 14;19:440–7. doi: 10.3762/bjoc.19.34 (PMC10113518; doi:10.3762/bjoc.19.34)
Supplement: File 1 — General procedures, experimental details, analytical data and copies of NMR spectra. [file Beilstein_J_Org_Chem-19-440-s001.pdf]

BEILSTEIN JOURNAL OF ORGANIC CHEMISTRY

Supporting Information

for

Mechanochemical solid state synthesis of copper(I)/NHC complexes with K_3PO_4

Ina Remy-Speckmann, Birte M. Zimmermann, Mahadeb Gorai, Martin Lerch and Johannes F. Teichert

Beilstein J. Org. Chem. **2023,** *19,* 440–447. doi:10.3762/bjoc.19.34

General procedures, experimental details, analytical data and copies of NMR spectra

License and Terms: This is a supporting information file under the terms of the Creative Commons Attribution License (https://creativecommons.org/licenses/by/4.0). Please note that the reuse, redistribution and reproduction in particular requires that the author(s) and source are credited and that individual graphics may be subject to special legal provisions.
The license is subject to the *Beilstein Journal of Organic Chemistry* terms and conditions: (https://www.beilstein-journals.org/bjoc/terms)

1 General information S3
1.1 Solvents S4
1.2 Reactions under H_2 pressure S4
1.3 Chemicals S4
2 General procedures S5
2.1 General procedure 1 – H_2-mediated reduction (GP1) S5
2.2 General procedure 2 – Ball mill synthesis attempts for [CuGua] **5** S6
3 Experimental details S7
3.1 Synthesis of [CuGua] **5** S7
3.2 Synthesis of standard Cu(I)/NHC-complexes S10
3.2.1 Synthesis of [Cu(IMes)Cl] (**7a**) S10
3.2.2 Synthesis of [Cu(SIMes)Cl] (**7b**) S10
3.2.3 Synthesis of [Cu(IPr)Cl] (**7c**) S11
3.2.4 Synthesis of [Cu(SIPr)Cl] (**7d**) S11
3.3 Products of catalytic ester reduction with H_2 .... S12
3.3 Evidence for the formation of **5**·CO_2 .... S15
4 References S17
5 Spectra S18

1 General information

Liquid state reactions were carried out in flame dried glassware under a nitrogen atmosphere using standard Schlenk techniques. Glassware and stir bars contaminated with transition metals were treated with *aqua regia* (conc. HCl/conc. HNO_3 3:1) prior to cleaning. For cleaning, glassware and stir bars were kept in an isoPrOH/KOH bath overnight, rinsed with H_2O, kept in a citric acid/H_2O bath overnight and finally rinsed with deionized H_2O and dried at 120 °C. Solutions and reagents were added with nitrogen-flushed disposable syringes/needles. Solvents were added using glass syringes and stainless-steel needles (stored at 120 °C). Analytical thin layer chromatography (TLC) was performed on silica gel 60 G/UV_{254} aluminium sheets (*Macherey-Nagel*). Ball-milling experiments were carried out using a *Fritsch* Pulverisette 7 *classic line* planetary ball mill. NMR spectra were recorded on AV400, AV500 or AV700 instruments (*Bruker*) at the Institut für Chemie of *Technische Universität Berlin*. Chemical shifts are reported in parts per million (ppm) and are referenced to the residual solvent resonance as the internal standard according to the standard literature.[1] Data are reported as follows: chemical shift, multiplicity (br s = broad singlet, s = singlet, d = doublet, t = triplet, q = quartet, m = multiplet, m_c = centrosymmetric multiplet), coupling constants (Hz), integration and – if possible – atom assignment. The assignment refers to the atom number shown in the corresponding molecule figure and was achieved by analysis of DEPT (DEPT 135) and 2D-NMR spectra (COSY, HMQC, HSQC, HMBC, NOESY). If a distinct assignment was not possible, atoms were marked with "*" and are interchangeable. Designation "*Ar*" refers to atoms of an aromatic system where a distinct assignment was not possible. Melting points (m.p.) were determined using a Leica Galen III melting point apparatus (*Wagner & Munz*). Infrared (IR) spectra were recorded on a Cary 630 FT-IR spectrometer equipped with an ATR unit (*Agilent Technologies).* Mass spectra (HRMS) were obtained from the Analytical Facility at the Institut für Chemie at *Technische Universität Berlin* (ESI/APCI: LTQ Orbitrap XL, *Thermo Scientific*; EI: GC-system 5975C, HP-5MS, *Agilent Technologies*). Analytical gas chromatography (GC) of reaction mixtures and pure substances was performed using a gas chromatograph 430-GC (*Varian Inc.*). The instrument was equipped with a FactorFour VF-WAXms capillary column (*Varian Inc., length*: 30 m, inner diameter: 0.25 mm, film thickness of the stationary phase: 0.25 µm). The following temperature program was used for the analysis: carrier gas N_2; injection temperature 270 °C; detector temperature 270 °C; flow rate 4.0 mL/min; temperature program: 40 °C start temperature, 20 °C/min heating rate to 250 °C for 10 min, then 20 °C/min heating rate to final temperature 260 °C for 5 min. The data was recorded with the program Galaxie 1.9.302.952 (*Varian Inc.*). Elemental analyses were obtained at *Technische Universität Chemnitz* using a vario MICRO cube (*Elementar*),

recording a CHNS analysis. Calibration is done beforehand using sulfanilic acid. Residual solvent was verified by ^{1}H.

1.1 Solvents

THF and 1,4-dioxane were dried over sodium/benzophenone and distilled under N_2 atmosphere prior to use. Et_3N, CH_2Cl_2 and MeOH were dried over CaH_2 and distilled under N_2 atmosphere prior to use. Acetone and EtOH were distilled under reduced pressure prior to use. Solvents (technical grade) for extraction/chromatography (*n*-pentane, cyclohexane, CH_2Cl_2, *tert*-butyl methyl ether, EtOAc) were distilled under reduced pressure prior to use. Liquid substrates for hydrogenation reactions were degassed prior to use.

1.2 Reactions under H_2 pressure

All reactions under H_2 pressure were carried out in glass vials (50 × 14 mm, *Schütt*), equipped with a magnetic stir bar and a rubber septum in autoclaves BR-100 or Br-300 (including the appropriate heating blocks, *Berghof*). The autoclave was purged with N_2 (3 × 10 bar) before the vials were placed in the autoclave and the septum was pierced under a counter flow of N_2. The autoclave was purged with N_2 (1 × 1 bar, 3 × 10 bar) and H_2 (3 × 10 bar) or D_2 (2 × 5 bar) before the appropriate H_2 or D_2 pressure was applied (pressure is given as initial pressure before heating). The heating block was pre-heated before the autoclave was placed inside. After the respective reaction time the autoclave was allowed to cool to rt and H_2 or D_2 was released. The autoclave was purged with N_2 (3 × 10 bar) before the vials were taken out.

1.3 Chemicals

All reagents were purchased from established commercial suppliers (*Sigma-Aldrich, Alfa Aesar, TCI, Acros, Strem, Merck, ABCR, Fluka, Fisher Scientific*) and used without further purification. NaO*t*-Bu was sublimed and stored in an Ar-filled glovebox. 15-crown-5 was dried over 3 Å MS, distilled under N_2 atmosphere and stored under N_2 over 3 Å MS. H_2 (99.999%) and D_2 (99.8%) was purchased from *Air Liquide*. Methyl 4-(1,3-dioxan-2-yl)benzoate (**10**)[2] and 1-(2-(2,3-diisopropyl-1-methylguanidino)ethyl)-3-mesityl-1*H*-imidazol-3-ium bromide (**3**)[3] was synthesized following known procedures.

2 General procedures

2.1 General procedure 1 – H_2-mediated reduction (GP1)

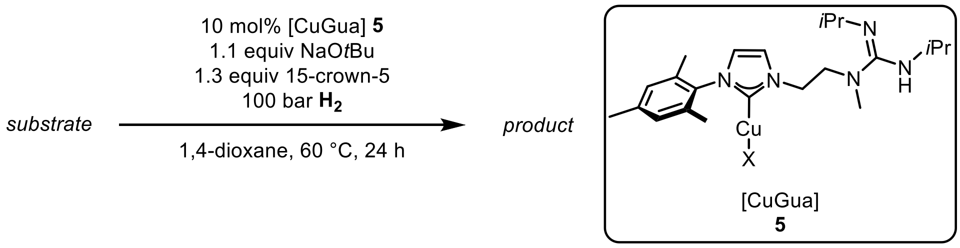

In an Ar-filled glovebox, [CuGua] **5** (28 mg, 6.0 µmol, 10 mol%) and NaO*t*Bu (63 mg, 0.66 mmol, 1.1 equiv) are placed in a 20 mL microwave vial with a stirring bar. The vial is capped inside the glovebox and then transferred outside. The solids are dissolved in 1,4-dioxane (2.8 mL). The mixture is stirred for 5 min at 40 °C. Dried 15-crown-5 (0.16 mL, 0.78 mmol, 1.3 equiv) is added to the reaction mixture at rt. The degassed substrates (0.60 mmol, 1.0 equiv) are dissolved in 1,4-dioxane (0.7 mL) and transferred to the reaction vial. The vial is placed in an autoclave and the septum is pierced with a needle under N_2-counterflow. The autoclave is purged with H_2 (3 × 10 bar). The reaction mixture is stirred for 24 h at 60 °C under H_2-atmosphere (100 bar). The crude reaction mixture is filtered over a small plug silica (eluant: CH_2Cl_2/MeOH, 50:1, 1 × 5 cm, 100 mL) and all volatiles are removed under reduced pressure. The crude products are purified by flash column chromatography on silica gel.

Notes: Before transferring the reaction mixture to the autoclave, it was necessary to have a tight closed reaction vial and to work under inert conditions. NaO*t*-Bu had to be sublimed and stored in a glovebox. 15-crown-5 had to be dried as described before. Between addition of the substrate and pressurizing with H_2 were approximately 5 min.

2.2 General procedure 2 – Ball mill synthesis attempts for [CuGua] 5

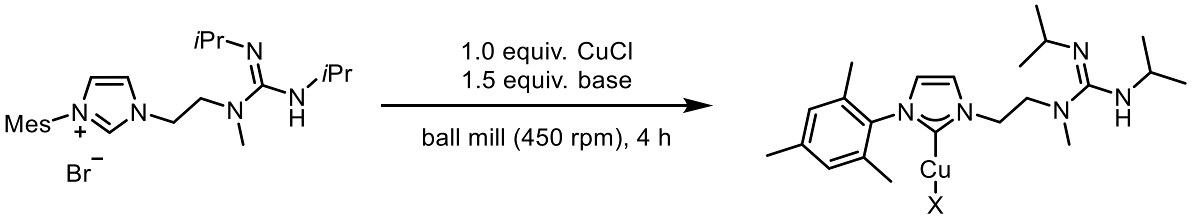

3 **5**

$C_{22}H_{35}ClCuN_5$
Mw = 468.55

The synthesis was carried out using the *Fritsch* Pulverisette 7 *classic line*, a high-energy planetary ball mill. The starting materials 1-(2-(2,3-diisopropyl-1-methylguanidino)ethyl)-3-mesityl-1*H*-imidazol-3-ium bromide (**3**, 75 mg, 0.16 mmol, 1.00 equiv), CuCl (16.5 mg, 0.16 mmol, 1.00 equiv) and a base (0.25 mmol, 1.50 equiv) were filled into a 12 mL steel vessel equipped with six steel balls (1 cm diameter). The beaker was sealed in an Ar-filled glovebox. Milling was carried out with 450 rpm for a total of four hours. After each hour the milling was paused for 30 minutes to avoid overheating of the machine. The ground product was mixed with CH_2Cl_2 (3 mL) and the resulting suspension was filtered over a PTFE syringe filter (0.45 µm). The filtrate was concentrated under reduced pressure.

Notes: For experiments with sodium hydride (NaH) as a base a 45 mL zirconia vessel with six zirconia balls (1.5 cm in diameter) was used.

3 Experimental details

3.1 Synthesis of [CuGua] 5

Scheme S2: Synthesis of [CuGua] **5**.[3]

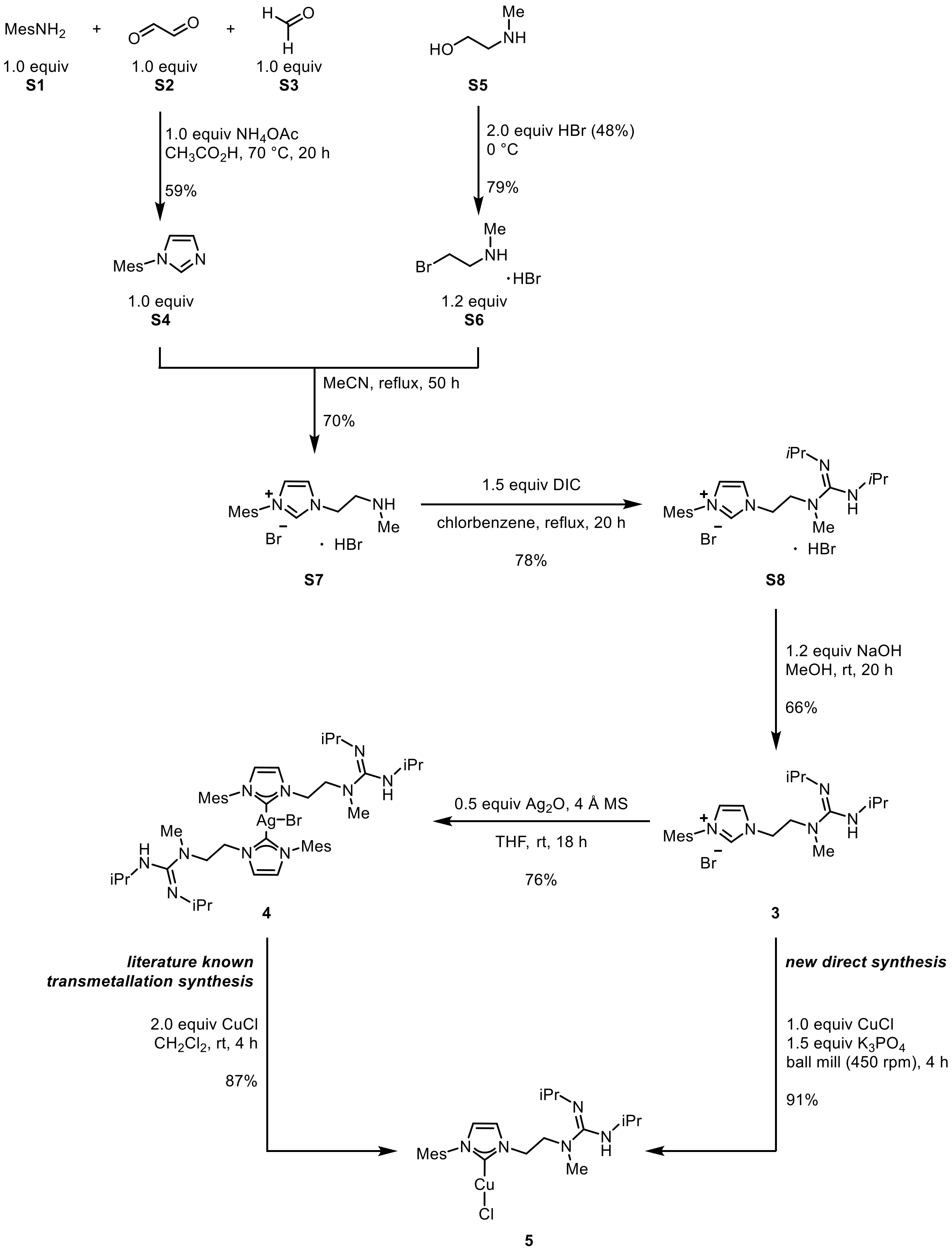

3.1.1 (1-(2-(2,3-Diisopropyl-1-methylguanidino)ethyl)-3-mesityl-1,3-dihydro-2*H*-imidazol-2-ylidene)copper(I) chloride (5)

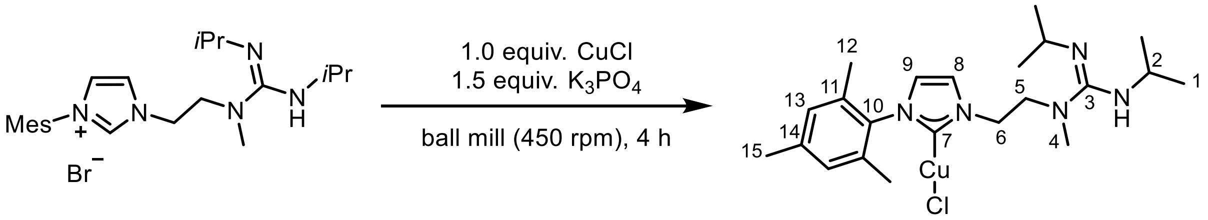

3

5
$C_{22}H_{35}ClCuN_5$
Mw = 468.55

The product was synthesized using a FRITSCH Pulverisette 7 *classic line*, a high-energy planetary ball mill. The starting materials 1-(2-(2,3-diisopropyl-1-methylguanidino)ethyl)-3-mesityl-1*H*-imidazol-3-ium bromide (**3**, 75 mg, 0.16 mmol, 1.00 equiv), CuCl (16.5 mg, 0.16 mmol, 1.00 equiv) and K_3PO_4 (53 mg, 0.25 mmol, 1.50 equiv) were filled into a 12 mL steel vessel equipped with six steel balls (1 cm diameter). The beaker was sealed in an Ar-filled glovebox. Milling was carried out with 450 rpm for a total of four hours. After each hour the milling was paused for 30 minutes to avoid overheating of the machine. The raw product was obtained as a grey powder after milling. The ground product was mixed with CH_2Cl_2 (3 mL) and the resulting suspension was filtered over a PTFE syringe filter (0.45 µm). The filtrate was concentrated under reduced pressure. The product **5** was obtained as a colourless solid (86 mg, 0.15 mmol, 91%).

^{1}H NMR (500 MHz, CD_2Cl_2): δ = 1.05–1.12 (m, 12H, H-1), 2.01 (s, 6H, H-12), 2.34 (s, 3H, H-15), 2.82 (s, 3H, H-4), 3.30–3.44 (m, 3H, N–*H*, H-2), 3.63 (t, $^3J_{5,6}$ = 6.2 Hz, 2H, H-5), 4.39 (t, $^3J_{6,5}$ = 6.2 Hz, 2H, H-6), 6.87 (d, $^3J_{8,9}$ = 1.6 Hz, 1H, H-8), 7.01 (s, 2H, H-13), 7.25 (d, $^3J_{9,8}$ = 1.5 Hz, 1H, H-9) ppm.

^{13}C NMR (126 MHz, CD_2Cl_2): δ = 18.0 (C-12), 21.2 (C-15), 23.9 (C-1a)*, 25.6 (C-1b)*, 38.7 (C-4), 46.8 (C-2a)**, 48.3 (C-2b)**, 49.3 (C-6), 52.2 (C-5), 121.8 (C-8), 122.2 (C-9), 129.5 (C-13), 135.3 (C-11), 136.0 (C-10), 139.7 (C-14), 155.9 (C-3), 179.1 (C-7) ppm.

HRMS (ESI) for $C_{22}H_{36}ClCuN_5^+$ [(M–Cl)$^+$]: calculated: 432.2188, found: 432.2179.

Elemental analysis: [CH_2Cl_2 + $C_{22}H_{35}ClCuN_5$]: calculated C: 49.91, found C: 50.22
calculated H: 6.74, found H: 6.72
calculated N: 12.65, found N: 12.92.

The data is in accordance with literature.[3]

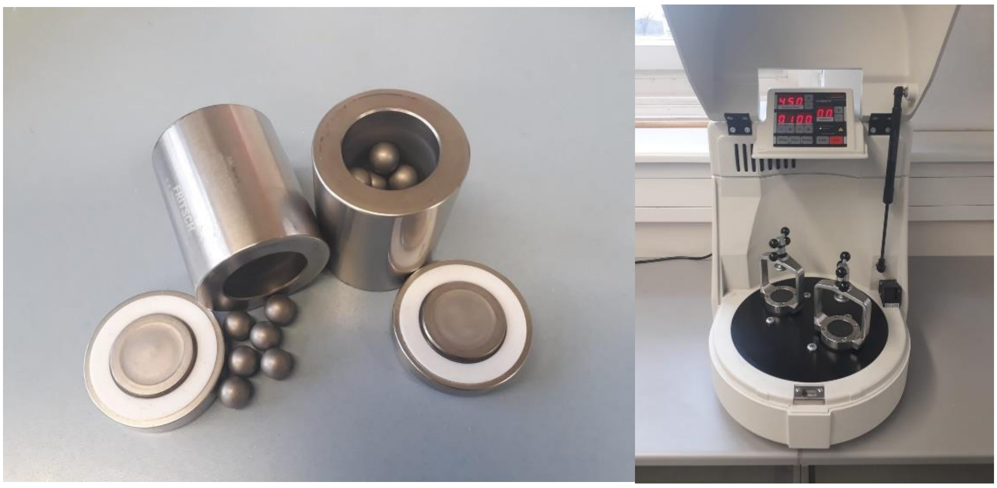

Figure S2: FRITSCH Pulverisette 7 *classic line*.

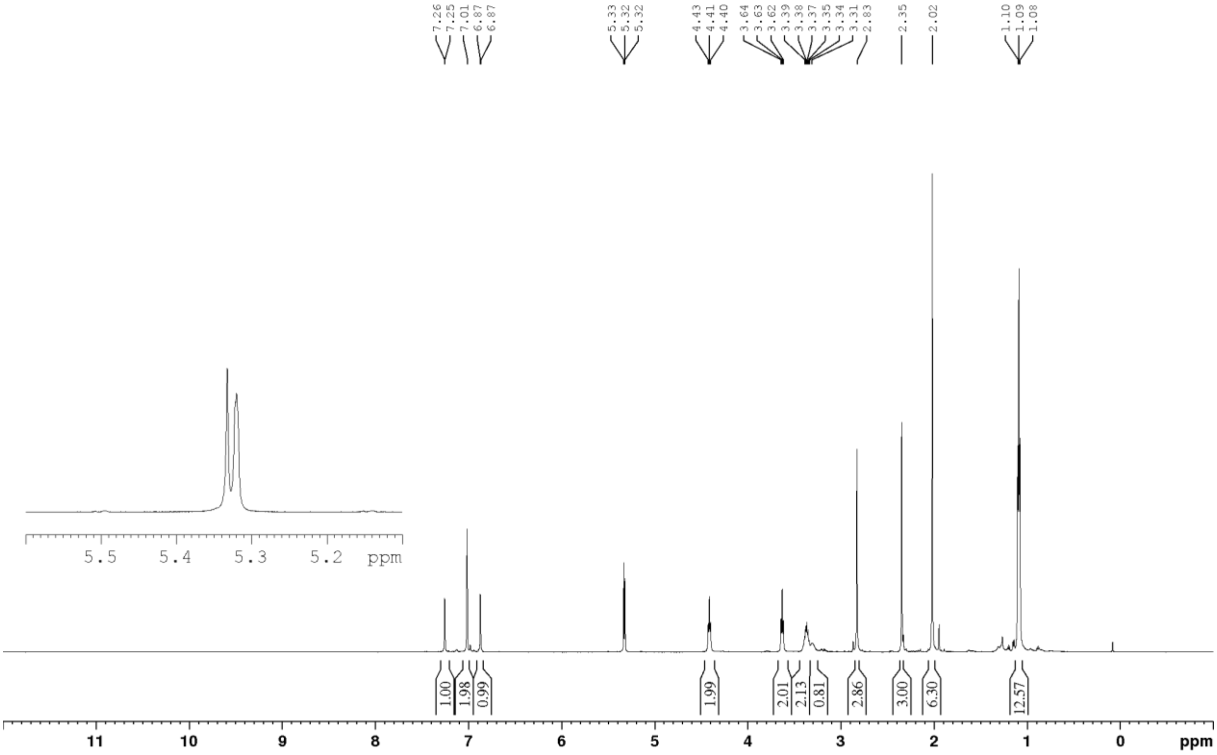

Figure S3: ^{1}H NMR in CD_2Cl_2 of **5** synthesized in a ball mill.

Nr.	Gewicht [mg]	Name	Methode	N-Fläche	C-Fläche	H-Fläche	S-Fläche	N [%]	C [%]	H [%]	S [%]	C/N Verh.
23	2.0040	CuGua-INA	2mgChem70s	10 002	27 238	12 146	0	12.97	50.29	6.733	0.000	3.8757
24	2.2570	CuGua-INA	2mgChem70s	11 182	30 731	13 731	0	12.86	50.36	6.724	0.000	3.9155
25	1.9250	CuGua-INA	2mgChem70s	9 558	26 018	11 582	0	12.92	50.01	6.697	0.000	3.8722

Figure S4: Elemental analysis of **5** synthesized in a ball mill.

3.2 Synthesis of standard Cu(I)/NHC-complexes

3.2.1 Synthesis of [Cu(IMes)Cl] (7a)

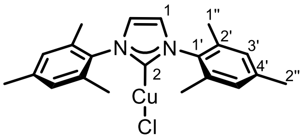

7a
$C_{21}H_{24}ClCuN_2$
M = 403.43 g/mol

Synthesis was carried out using the FRITSCH Pulverisette 7 *classic line*, a high-energy planetary ball mill. The starting materials 1,3-bis-(2,4,6-trimethylphenyl)-imidazolium chloride (IMesCl, **6a** (75 mg, 0.22 mmol, 1.00 equiv), CuCl (21.8 mg, 0.22 mmol, 1.00 equiv) and K_3PO_4 (70.1 mg, 0.33 mmol, 1.50 equiv) were filled into a 12 mL steel vessel equipped with six steel balls (1 cm diameter). The beaker was sealed in an Ar-filled glovebox. Milling was carried out with 450 rpm for a total of four hours. After each hour the milling was paused for 30 minutes to avoid overheating of the machine. The ground product was mixed with CH_2Cl_2 (3 mL) and the resulting suspension was filtered over a PTFE syringe filter (0.45 µm). The filtrate was concentrated under reduced pressure. The product **7a** was obtained as a colourless solid (63.4 mg, 0.15 mmol, 71% [8% cationic dimer complex]).

^{1}H NMR (500 MHz, $CDCl_3$): δ = 2.10 (s, 12H, H-1''), 2.34 (s, 6H, H-2''), 6.99 (s, 4H, H-3'), 7.05 (s, 2H, H-1) ppm.

^{1}H NMR for cationic dimer complex (500 MHz, $CDCl_3$): δ = 1.68 (s, 24H, H-1''), 2.43 (s, 12H, H-2''), 6.89 (s, 8H, H-3'), 7.10 (s, 4H, H-1) ppm.

HRMS (ESI) for $C_{23}H_{27}CuN_3^+$ [(M(MeCN))–Cl^+]: calculated: 408.1501, found: 408.1497.

Data is in accordance with literature.[4] The amount of the cationic dimer complex was calculated from ^{1}H-NMR spectra using the integrals of the signals at 7.05 ppm for the desired complex and 7.10 ppm for the cationic dimer complex.

3.2.2 Synthesis of [Cu(SIMes)Cl] (7b)

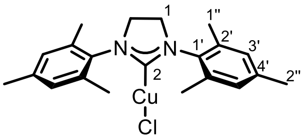

7b
$C_{21}H_{26}ClCuN_2$
M = 405.45 g/mol

Synthesis was carried out using the FRITSCH Pulverisette 7 *classic line*, a high-energy planetary ball mill. The starting materials 1,3-bis(2,4,6-trimethylphenyl)imidazolinium chloride (SIMesCl, **6b**) (75 mg, 0.22 mmol, 1.00 equiv), CuCl (21.7 mg, 0.22 mmol, 1.00 equiv) and K_3PO_4 (0.70 mg, 0.33 mmol, 1.50 equiv) were filled into a 12 mL steel vessel equipped with six steel balls (1 cm diameter). The beaker was sealed in an Ar-filled glovebox. Milling was carried out with 450 rpm for a total of four hours. After each hour the milling was paused for 30 minutes to avoid overheating of the machine. The ground product was mixed with CH_2Cl_2 (3 mL) and the resulting suspension was filtered over a PTFE syringe filter (0.45 µm). The filtrate was concentrated under reduced pressure. The product **7b** was obtained as a colourless solid (64.5 mg, 0.16 mmol, 73%).

^{1}H NMR (500 MHz, $CDCl_3$): δ = 12.29 (s, 6H, H-2''), 2.31 (s, 12H, H-1''), 3.96 (s, 4H, H-1), 6.95 (s, 4H, H-3') ppm.

HRMS (ESI) for $C_{23}H_{29}CuN_3^+$ [(M(MeCN))–Cl+]: calculated: 410.1657, found: 410.1652.
The data is in accordance with literature.[4]

3.2.3 Synthesis of [Cu(IPr)Cl] (7c)

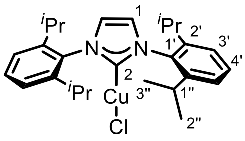

7c
$C_{27}H_{36}ClCuN_2$
M = 487.60 g/mol

Synthesis was carried out using the FRITSCH Pulverisette 7 *classic line*, a high-energy planetary ball mill. The starting materials 1,3-bis-(2,6-diisopropylphenyl)imidazolium chloride (IPrCl, **6c**) (75 mg, 0.18 mmol, 1.00 equiv), CuCl (17.5 mg, 0.18 mmol, 1.00 equiv) and K_3PO_4 (56.2 mg, 0.27 mmol, 1.50 equiv) were filled into a 12 mL steel vessel equipped with six steel balls (1 cm diameter). The beaker was sealed in an Ar-filled glovebox. Milling was carried out with 450 rpm for a total of six hours. After each hour the milling was paused for 30 minutes to avoid overheating of the machine. The ground product was mixed with CH_2Cl_2 (3 mL) and the resulting suspension was filtered over a PTFE syringe filter (0.45 µm). The resulting solution was filtered over silica and the filtrate was concentrated under reduced pressure. The product **7c** was obtained as a colourless solid (54.0 mg, 0.11 mmol, 63%).

^{1}H NMR (500 MHz, $CDCl_3$): δ = 1.22 (d, ^3J = 6.9 Hz, 12H, H-2''*), 1.30 (d, ^3J = 6.9 Hz, 12H, H-3''*), 2.56 (sept, ^3J = 6.9 Hz, 4H, H-1''), 7.12 (s, 2H, H-1), 7.29 (d, ^3J = 7.8 Hz. 4H, H-3'), 7.49 (t, ^3J = 7.8 Hz, 2H, H-4') ppm.
HRMS (ESI) for $C_{29}H_{39}CuN_3^+$ [(M(MeCN))–Cl+]: calculated: 492.2440, found: 492.2430.
Data is in accordance with literature.[5]

3.2.4 Synthesis of [Cu(SIPr)Cl] (7d)

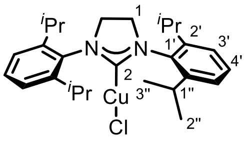

7d
$C_{27}H_{38}ClCuN_2$
M = 489.61 g/mol

Synthesis was carried out using the FRITSCH Pulverisette 7 *classic line*, a high-energy planetary ball mill. The starting materials 1,3-Bis-(2,6-diisopropylphenyl)-imidazolinium chloride (SIPrCl, **6d**) (75 mg, 0.18 mmol, 1.00 equiv), CuCl (17.4 mg, 0.18 mmol, 1.00 equiv) and K_3PO_4 (55.9 mg, 0.26 mmol, 1.50 equiv) were filled into a 12 mL steel vessel equipped with six steel balls (1 cm diameter). The beaker was sealed in an Ar-filled glovebox. Milling was carried out with 450 rpm for a total of four hours. After each hour the milling was paused for 30 minutes to avoid overheating of the machine. The ground product was mixed with CH_2Cl_2 (3 mL) and the resulting suspension was filtered over a PTFE syringe filter (0.45 µm). The filtrate was concentrated under reduced pressure. The product **7d** was obtained as a colourless solid (55.1 mg, 0.11 mmol, 64% [12% cationic dimer complex]).

HRMS (ESI) for $C_{29}H_{41}CuN_3^+$ [(M(MeCN))–Cl+]: calculated: 494.2596, found: 494.2588.

^{1}H NMR (500 MHz, $CDCl_3$): δ = 1.34 (d, ^3J = 6.9 Hz, 12H, H-2''*), 1.37 (d, ^3J = 6.9 Hz, 12H, H-3''*), 3.00-3.11 (m, 5H, H-1''), 4.01 (s, 4H, H-1), 7.24 (d, ^3J = 7.8 Hz. 4H, H-3'), 7.40 (t, ^3J = 7.8 Hz, 2H, H-4') ppm.
^{1}H NMR for cationic dimer complex (500 MHz, $CDCl_3$): δ = 1.26 (d, ^3J = 6.8 Hz, 24H, H-2''*), 1.41 (d, ^3J = 6.8 Hz, 24H, H-3''*), 3.00-3.11 (m, 10H, H-1''), 4.88 (s, 8H, H-1), 7.29 (d, ^3J = 7.8 Hz, 8H, H-3'), 7.40 (t, $^3J_{5,6}$ = 7.8 Hz, 4H, H-4') ppm.
Data is in accordance with literature.[5] The amount of the cationic dimer complex was calculated from ^{1}H NMR spectra using the integrals of the signals at 7.40 ppm for the desired complex and 7.48 ppm for the cationic dimer complex. The signal at 3.00–3.11 ppm integrates too high because of an overlap of signals for the desired complex and the cationic dimer complex.

3.3 Products of catalytic ester reduction with H_2

3.3.1 Benzyl alcohol (9)

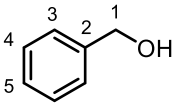

9
C_7H_8O
Mw = 108.14

Prepared according to **GP1** from benzaldehyde (**14**, 42 µL, 0.40 mmol, 1.0 equiv), [CuGua] **5bm** (19 mg, 40 µmol, 10 mol %), NaO*t*-Bu (42 mg, 0.44 mmol, 1.1 equiv), 15-crown-5 (0.10 ml, 0.52 mmol, 1.3 equiv.) in 1,4-dioxane (3.0 mL). The reaction mixture was stirred for 24 h at 70 °C. After GC analysis 95% conversion to the alcohol **9** was achieved. Purification by flash column chromatography on silica gel (*n*-pentane/*tert*-butyl methyl ether = 5:1) yielded **9** as colourless oil (34 mg, 0.32 mmol, 79%)

R_f = 0.43 (SiO_2, *n*-pentane/*tert*-butyl methyl ether = 1:1).

^{1}H NMR (500 MHz, $CDCl_3$): δ = 1.89 (br s, 1H, O–*H*), 4.69 (s, 2H, H-1), 7.28–7.33 (m, 1H, H-5), 7.36–7.38 (m, 4H, H-3, H-4) ppm.

^{13}C NMR (126 MHz, $CDCl_3$): δ = 65.4 (C-1), 127.0 (C-3), 127.7 (C-5), 128.6 (C-4), 140.9 (C-2) ppm.

HRMS (APCI) for $C_7H_7^+$ [(M–OH)$^+$] calculated: 91.0548, found: 91.0542.
The data is in accordance with literature.[6]

3.3.2 Benzyl alcohol (9)

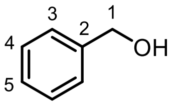

9

C_7H_8O

Mw = 108.14

Prepared according to **GP1** from ethylbenzoate (**8**, 60 µL, 0.40 mmol, 1.0 equiv), [CuGua] **5bm** (19 mg, 40 µmol, 10 mol %), NaO*t*-Bu (42 mg, 0.44 mmol, 1.1 equiv), 15-crown-5 (0.10 ml, 0.52 mmol, 1.3 equiv) in 1,4-dioxane (3.0 mL). The reaction mixture was stirred for 24 h at 70 °C. After GC analysis 65% conversion to the alcohol **9** was achieved. Purification by flash column chromatography on silica gel (*n*-pentane/*tert*-butyl methyl ether = 5:1) yielded **9** as colourless oil (23 mg, 0.21 mmol, 53%).

$\mathbf{R}_f$ = 0.43 (SiO_2, *n*-pentane/*tert*-butyl methyl ether = 1:1).

^{1}H NMR (500 MHz, $CDCl_3$): δ = 1.99 (br s, 1H, O–*H*), 4.68 (s, 2H, H-1), 7.28–7.33 (m, 1H, H-5), 7.37–7.40 (m, 4H, H-3, H-4) ppm.
^{13}C NMR (126 MHz, $CDCl_3$): δ = 65.4 (C-1), 127.0 (C-3), 127.7 (C-5), 128.6 (C-4), 140.9 (C-2) ppm.

HRMS (APCI) for $C_7H_7^+$ [$(M–OH)^+$] calculated: 91.0548, found: 91.0542.
The data is in accordance with literature.[6]

3.3.3 (4-(1,3-Dioxan-2-yl)phenyl)methanol (11)

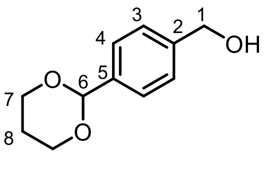

11

$C_{11}H_{14}O_3$

Mw = 194.23

Prepared according to **GP1** from methyl 4-(1,3-dioxan-2-yl)benzoate (**10**, 78 mg, 40 mmol, 1.0 equiv), [CuGua] **5bm** (19 mg, 40 µmol, 10 mol%), NaO*t*-Bu (42 mg, 0.44 mmol, 1.1 equiv), 15-crown-5 (0.10 ml, 0.52 mmol, 1.3 equiv) in 1,4-dioxane (3.0 mL). The reaction mixture was stirred for 24 h at 70 °C. After GC analysis 74% conversion to the alcohol **11** was achieved. Purification by flash column chromatography on silica gel (*n*-pentane/*tert*-butyl methyl ether = 1:1) yielded **11** as white solid (51 mg, 0.26 mmol, 66%)

Mp: 98 °C. $\mathbf{R}_f$ = 0.27 (SiO_2, *n*-pentane/*tert*-butyl methyl ether = 1:1).

^{1}H NMR (500 MHz, $CDCl_3$): δ = 1.43 (d sept, $^2J_{8\alpha,8\beta}$ = 13.5 Hz, $^3J_{8\alpha,7}$ = 1.5 Hz, 1H, H-8α), 2.00 (t, $^3J_{O–H,1}$ = 5.7 Hz, 1H, O–*H*), 2.22 (m, 1H, H-8β), 3.98 (m, 2H, H-7), 4.26 (m, 2H, H-7), 4.63 (d, $^3J_{1,O–H}$ = 5.4 Hz, 2H, H-1), 5.49 (s, 1H, H-6), 7.32 (d, $^3J_{3,4}$ = 8.2 Hz, 2H, H-3), 7.45 (d, $^3J_{4,3}$ = 8.2 Hz, 2H, H-4) ppm.
^{13}C NMR (126 MHz, $CDCl_3$): δ = 25.7 (C-8), 65.0 (C-1), 67.4 (C-7), 101.5 (C-6), 126.2 (C-3), 126.7 (C-4), 138.0 (C-5), 141.6 (C-2) ppm.
HRMS (APCI) for $C_{11}H_{15}O_3^+$ [$(M+H)^+$] calculated: 195.1016, found: 195.1017.

IR (ATR): $\tilde{\nu}$ = 3317(m), 2938(m), 2911(m), 2863(m), 2733(m), 2301(m), 2218(w), 2087(w), 2004(w), 1920(w), 1824(w), 1651(w), 1466(m), 1381(s), 1353(m), 1282(m), 1236(m), 1124(m), 1010(m), 987(m), 829(s), 773(s), 665(m) cm^{-1}.

3.3.4 (*Z*)-1,2-Diphenylethene (13)

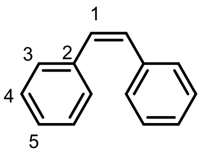

13
$C_{14}H_{12}$
Mw = 180.25

Prepared according to **GP1** from diphenylacetylene (**12**, 72 mg, 0.40 mmol, 1.0 equiv), [CuGua] **5bm** (19 mg, 40 µmol, 10 mol %), NaO*t*-Bu (42 mg, 0.44 mmol, 1.1 equiv), 15-crown-5 (0.10 ml, 0.52 mmol, 1.3 equiv) in 1,4-dioxane (3.0 mL). The reaction mixture was stirred for 24 h at 70 °C. After GC analysis full conversion to the alkene **13** was achieved. Purification by flash column chromatography on silica gel (*n*-pentane) yielded **13** as colourless oil (62 mg, 0.34 mmol, 86%).

R*f* = 0.95 (SiO_2, *n*-pentane).
¹H NMR (500 MHz, $CDCl_3$): δ = 6.64 (s, 2H, H-1), 7.20–7.24 (m, 2H, H-5), 7.24–7.27 (m, 4H, H-4), 7.28–7.30 (m, 4H, H-3) ppm.
¹³C NMR (126 MHz, $CDCl_3$): δ = 127.1 (C-5), 128.2 (C-3), 128.9 (C-4), 130.3 (C-1), 137.3 (C-2) ppm.
HRMS (APCI) for $C_{14}H_{12}^{\cdot+}$ [$M^{\cdot+}$] calculated: 180.0939, found: 180.0933. The data is in accordance with literature.[7]

3.3.5 1-Phenylethan-1-ol (16)

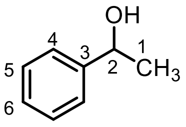

16
$C_8H_{10}O$
Mw = 122.17

Prepared according to **GP1** from acetophenone (**15**, 48 µL, 0.40 mmol, 1.0 equiv), [CuGua] **5bm** (19 mg, 40 µmol, 10 mol %), NaO*t*-Bu (42 mg, 0.44 mmol, 1.1 equiv), 15-crown-5 (0.10 ml, 0.52 mmol, 1.3 equiv) in 1,4-dioxane (3.0 ml). The reaction mixture was stirred for 24 h at 70 °C. After GC analysis full conversion to the alcohol **16** was achieved. Purification by flash column chromatography on silica gel (*n*-pentane/*tert*-butyl methyl ether = 5:1) yielded **16** as colourless oil (36 mg, 0.29 mmol, 73%)

R*f* = 0.47 (SiO_2, *n*-pentane/*tert*-butyl methyl ether = 1:1).
¹H NMR (500 MHz, $CDCl_3$): δ = 1.50 (d, $^3J_{1,2}$ = 6.5 Hz, 3H, H-1), 2.00 (d, $^3J_{O\text{–}H,2}$ = 3.6 Hz, 1H, O–*H*), 4.89 (dq, $^3J_{2,O\text{–}H}$ = 3.6 Hz, $^3J_{2,1}$ = 6.5 Hz, 1H, H-2), 7.26–7.30 (m, 1H, H-6), 7.34–7.39 (m, 2H, H-4, H-5) ppm.

¹³C NMR (126 MHz, $CDCl_3$): δ = 25.2 (C-1), 70.4 (C-2), 125.4 (C-4), 127.5 (C-6), 128.5 (C-5), 145.8 (C-3) ppm.

HRMS (APCI) for $C_8H_9^+$ [(M–OH)$^+$] calculated: 105.0704, found: 105.0698.
The data is in accordance with literature.[8]

3.3 Evidence for the formation of 5·CO_2

Attempts were carried out to isolate and characterize the suggested CO_2 adduct of complex **5**. However, the complex could not be isolated due to limited stability. Nevertheless, the crude ^{1}H NMR spectrum as follows shows that all key resonances are present. Further evidence comes from the HRMS of the crude mixture as depicted below.

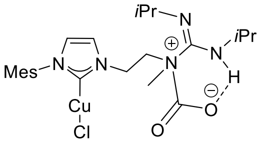

5•CO_2
$C_{23}H_{35}ClCuN_5O_2$
Mw = 512.56
calculated [M] + H$^+$: 512.1848

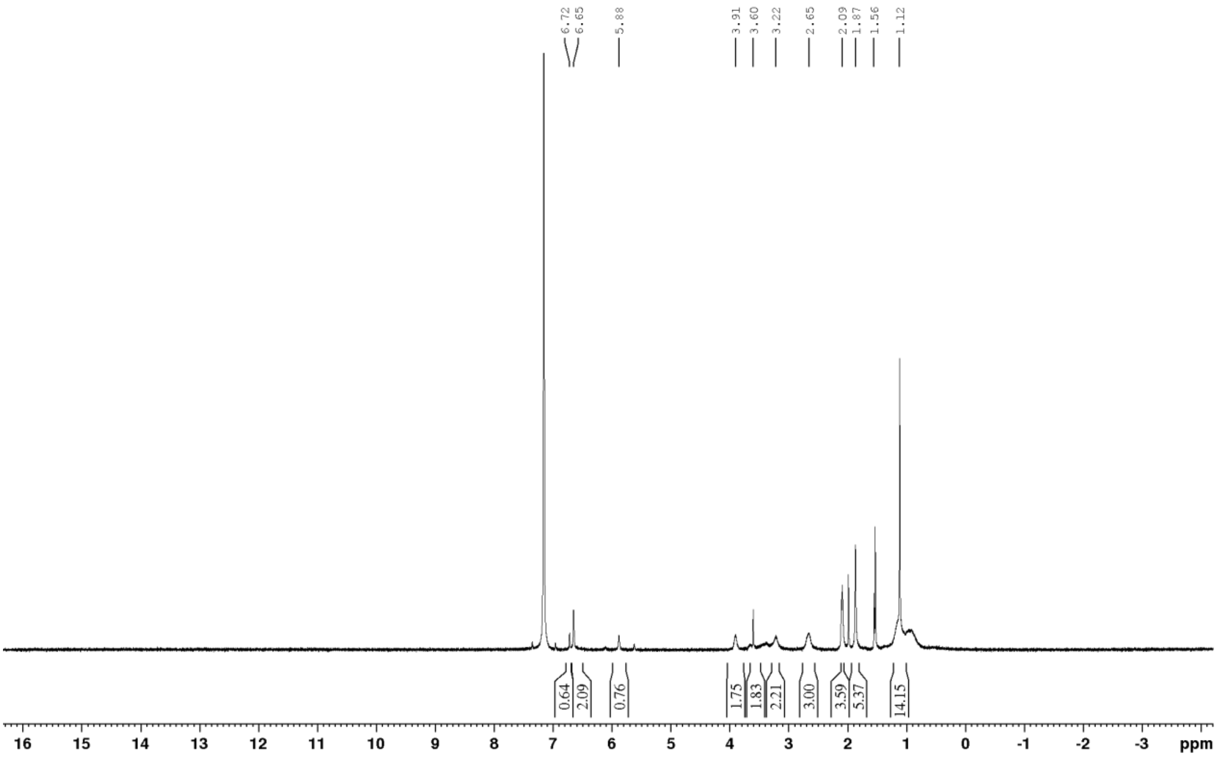

Figure S5: Crude ^{1}H NMR of **5·**CO_2.

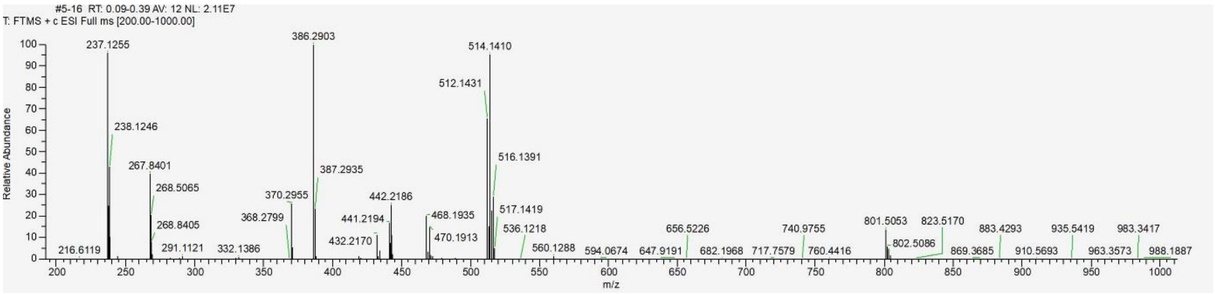

Figure S6: Mass spectrum of **5**·CO_2.

In addition to these data, reactivity studies further support the formation of a CO_2 adduct with complex **5**, and that this adduct is catalytically inactive: When the standard catalytic hydrogenation of ethyl benzoate was carried out under standard conditions,[9] but CO_2 was bubbled through the solution of **5** at rt for 5 minutes prior to the catalytic reaction, no conversion in the catalytic hydrogenation of esters was observed.

This shows that coordination of CO_2 to the complex prevents any reactivity in the catalytic ester hydrogenation. This finding is in support of our hypothesis that the guanidine subunit is turned into a hydrogen bond donor upon protonation in situ. Complexation of CO_2 prevents the formation of any hydrogen bonds to the ester substrate, resulting in an inactive catalyst.

We have furthermore measured the elemental analysis of the CO_2 adduct of **5** after several months of storage in an Ar filled glovebox:

Elemental analysis: [$C_{23}H_{36}ClCuN_5O_2$]: calculated C: 53.79, found C: 53.67
calculated H: 7.07, found H: 7.16
calculated N: 13.64, found N: 12.36.

4 References

[1] a) G. R. Fulmer, A. J. M. Miller, N. H. Sherden, H. E. Gottlieb, A. Nudelman, B. M. Stoltz, J. E. Bercaw, K. I. Goldberg, *Organometallics* **2010**, *29*, 2176; b) H. E. Gottlieb, V. Kotlyar, A. Nudelman, *J. Org. Chem.* **1997**, *62*, 7512.

[2] S. He, G. Dong, Z. Wang, W. Chen, Y. Huang, Z. Li, Y. Jiang, N. Liu, J. Yao, Z. Miao et al., *ACS Med. Chem. Lett.* **2015**, *6*, 239.

[3] C.-C. Tai, M.-S. Yu, Y.-L. Chen, W.-H. Chuang, T.-H. Lin, G. P. A. Yap, T.-G. Ong, *Chem. Commun.* **2014**, *50*, 4344.

[4] G. Pisanò, C. S. J. Cazin, *Green Chem.* **2020**, *22*, 5253.

[5] O. Santoro, A. Collado, A. M. Z. Slawin, S. P. Nolan, C. S. J. Cazin, *Chem. Commun.* **2013**, *49*, 10483.

[6] R. Cano, M. Yus, D. J. Ramón, *Tetrahedron* **2011**, *67*, 8079.

[7] F. Pape, N. O. Thiel, J. F. Teichert, *Chem. Eur. J.* **2015**, *21*, 15934.

[8] S.-F. Hsu, B. Plietker, *Chem. Eur. J.* **2014**, *20*, 4242.

[9] B.M. Zimmermann, T. Tran Ngoc, D.-I. Tzaras, T. Kaicharla, J.F. Teichert, *J. Am. Chem. Soc.* **2021**, *143*, 16865–16873.

5 Spectra

(1-(2-(2,3-Diisopropyl-1-methylguanidino)ethyl)-3-mesityl-1,3-dihydro-2*H*-imidazol-2-ylidene)copper(I) chloride in CD_2Cl_2 (5) *via* ball mill

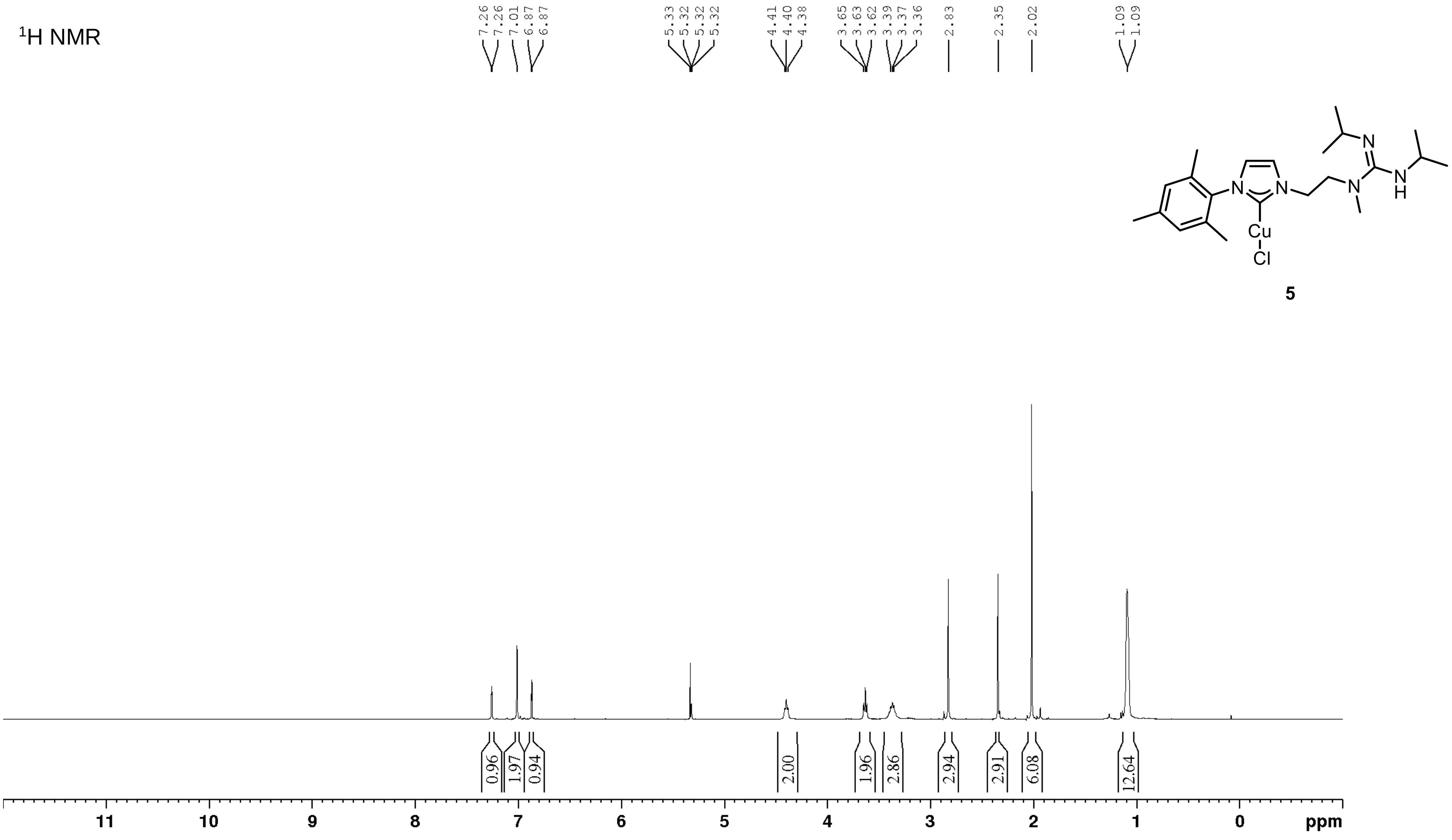

5

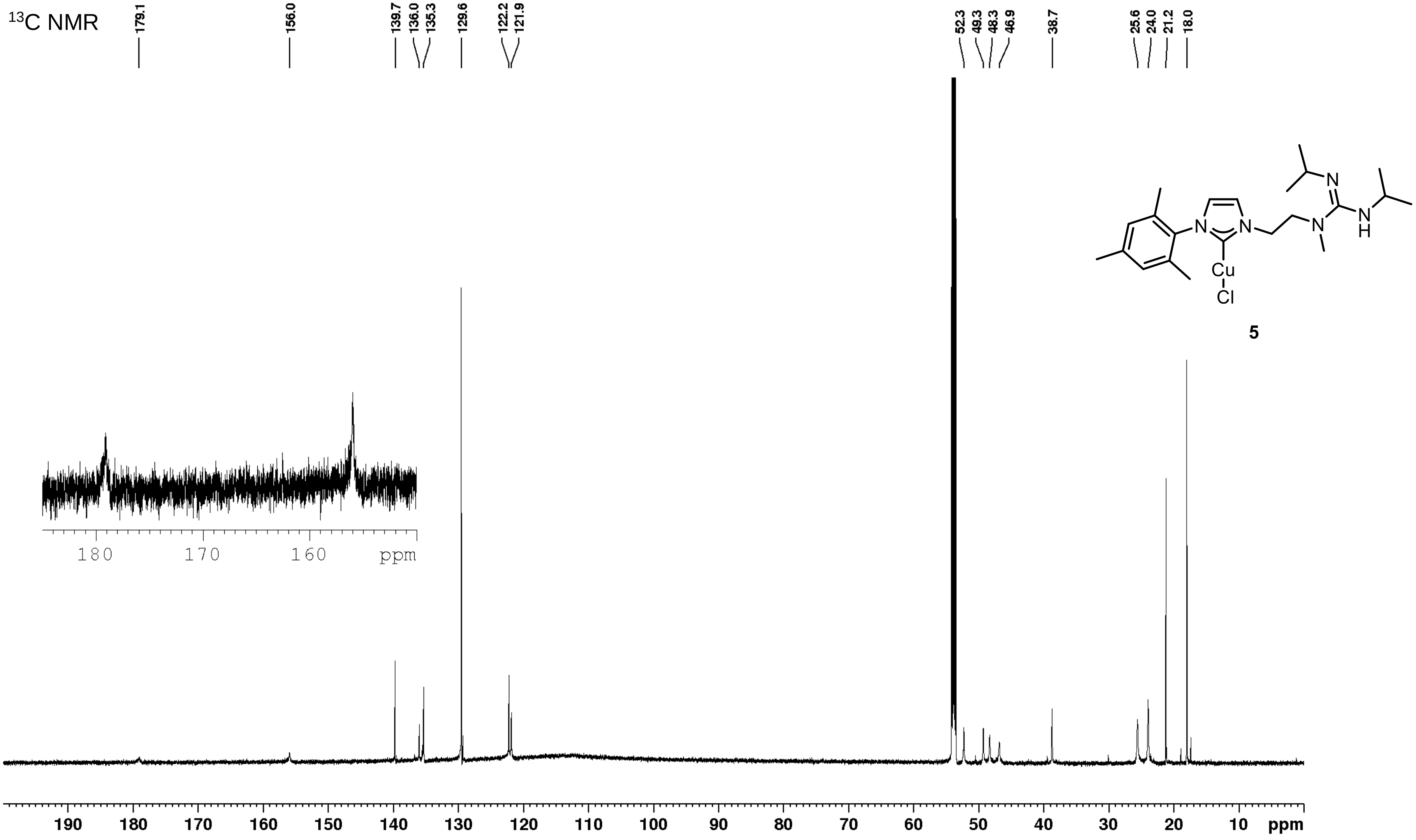

13C NMR
179.1
156.0
139.7
136.0
135.3
129.6
122.2
121.9
52.3
49.3
48.3
46.9
38.7
25.6
24.0
21.2
18.0
N
N
N
N
N
H
Cu
Cl
5
180
170
160
ppm
190
180
170
160
150
140
130
120
110
100
90
80
70
60
50
40
30
20
10
ppm

^{1}H-^{1}H COSY

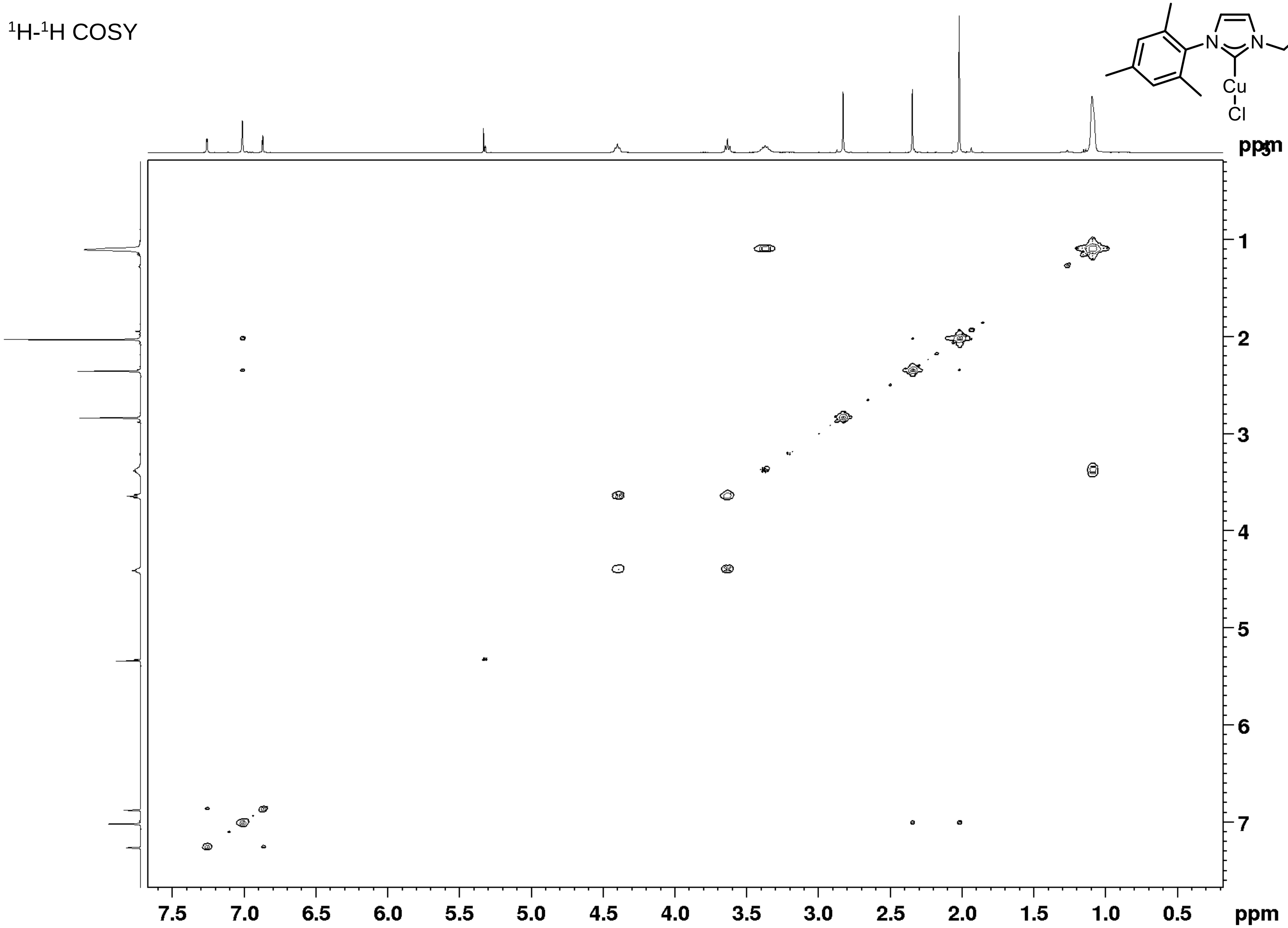

ppm
7.5
7.0
6.5
6.0
5.5
5.0
4.5
4.0
3.5
3.0
2.5
2.0
1.5
1.0
0.5
1
2
3
4
5
6
7

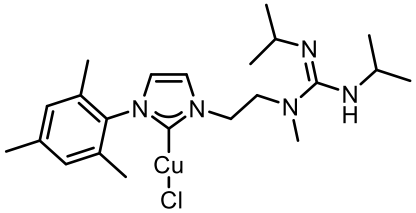

N
N
Cu
Cl
N
N
N
H

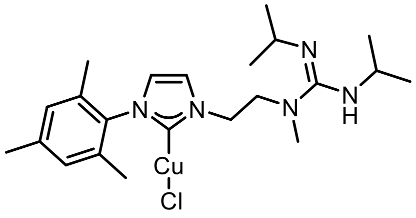

Cu
Cl

5

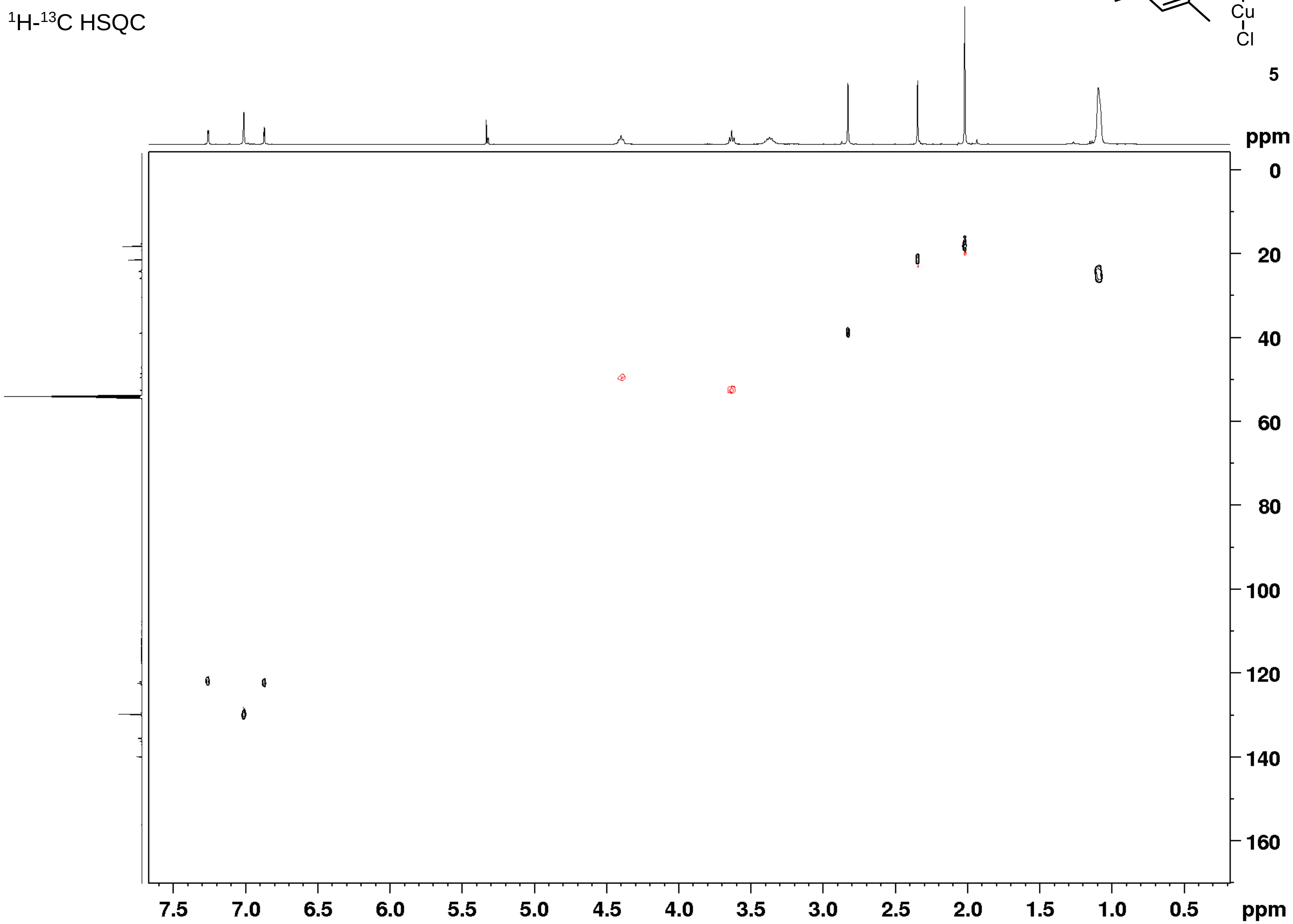

^{1}H-^{13}C HSQC
ppm
0
20
40
60
80
100
120
140
160
7.5
7.0
6.5
6.0
5.5
5.0
4.5
4.0
3.5
3.0
2.5
2.0
1.5
1.0
0.5
ppm

^{1}H-^{13}C HMBC

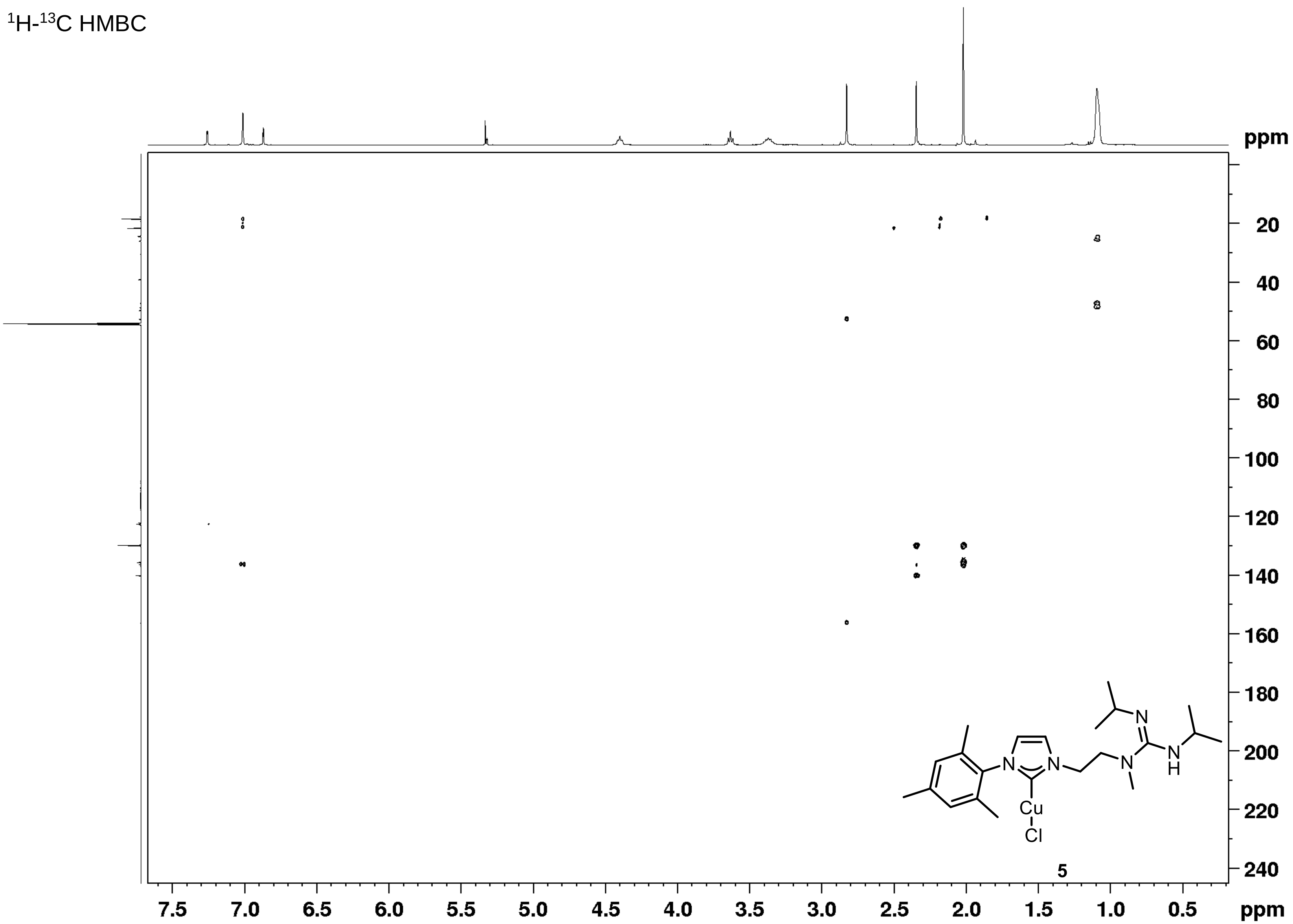
ppm
20
40
60
80
100
120
140
160
180
200
220
240
7.5
7.0
6.5
6.0
5.5
5.0
4.5
4.0
3.5
3.0
2.5
2.0
1.5
1.0
0.5
ppm
N
N
N
N
N
H
Cu
Cl
5

HRMS

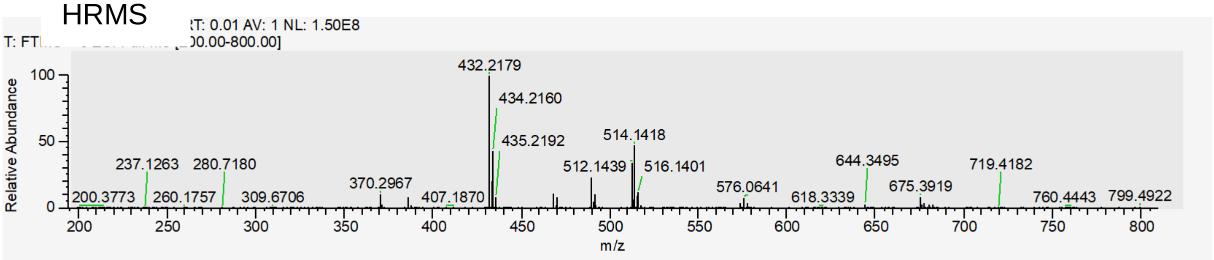
RT: 0.01 AV: 1 NL: 1.50E8
T: FT
00.00-800.00]
Relative Abundance
100
50
0
432.2179
434.2160
435.2192
514.1418
512.1439
516.1401
237.1263
280.7180
200.3773
260.1757
309.6706
370.2967
407.1870
576.0641
618.3339
644.3495
675.3919
719.4182
760.4443
799.4922
200
250
300
350
400
450
500
550
600
650
700
750
800
m/z

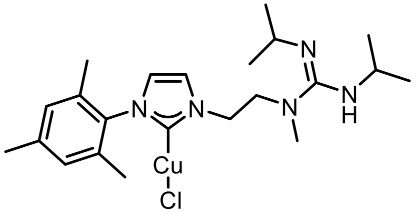
N
N
N
N
N
H
Cu
Cl

5

(*Z*)-1,2-Diphenylethene (13)

^{1}H NMR

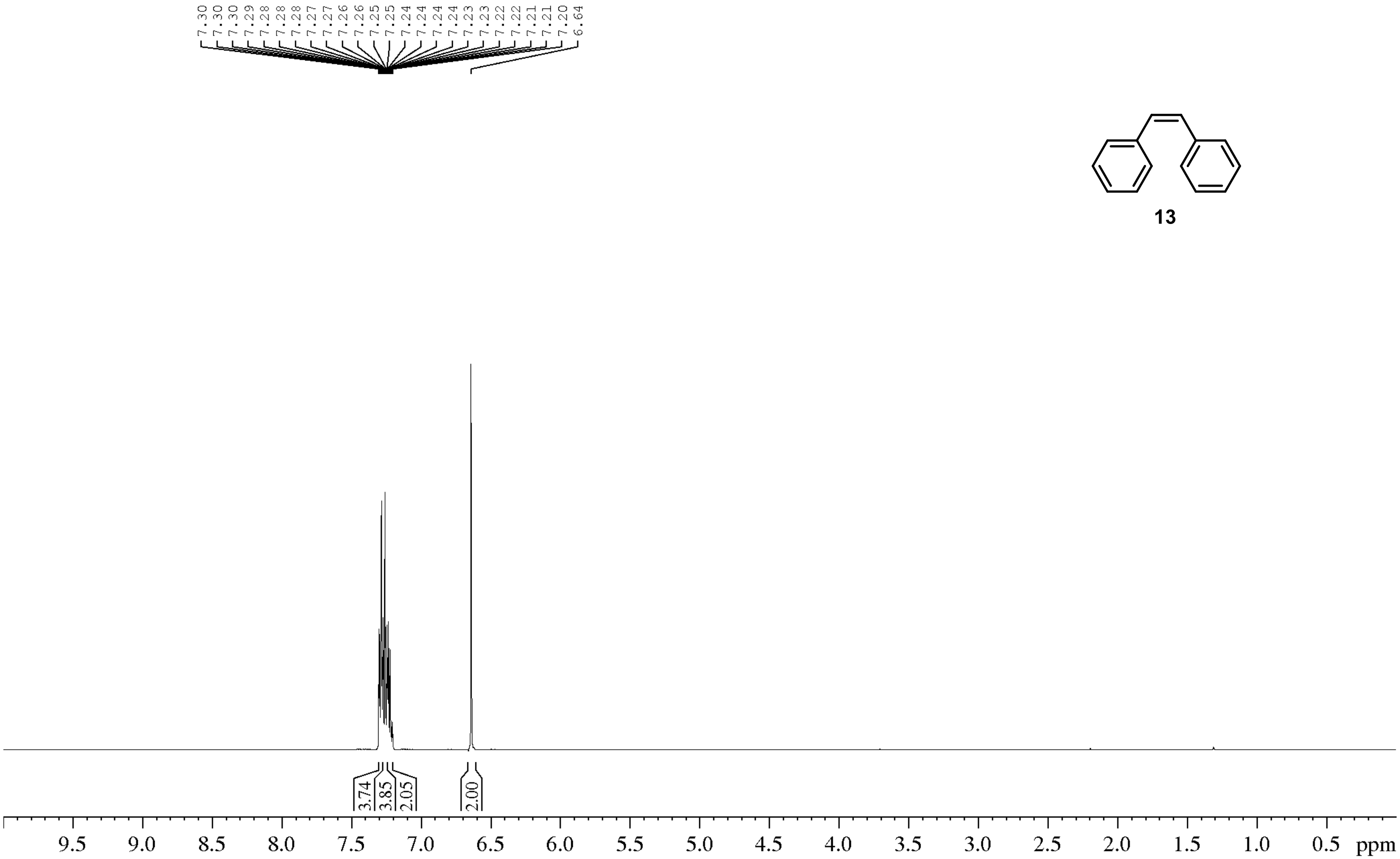

13

^{13}C NMR

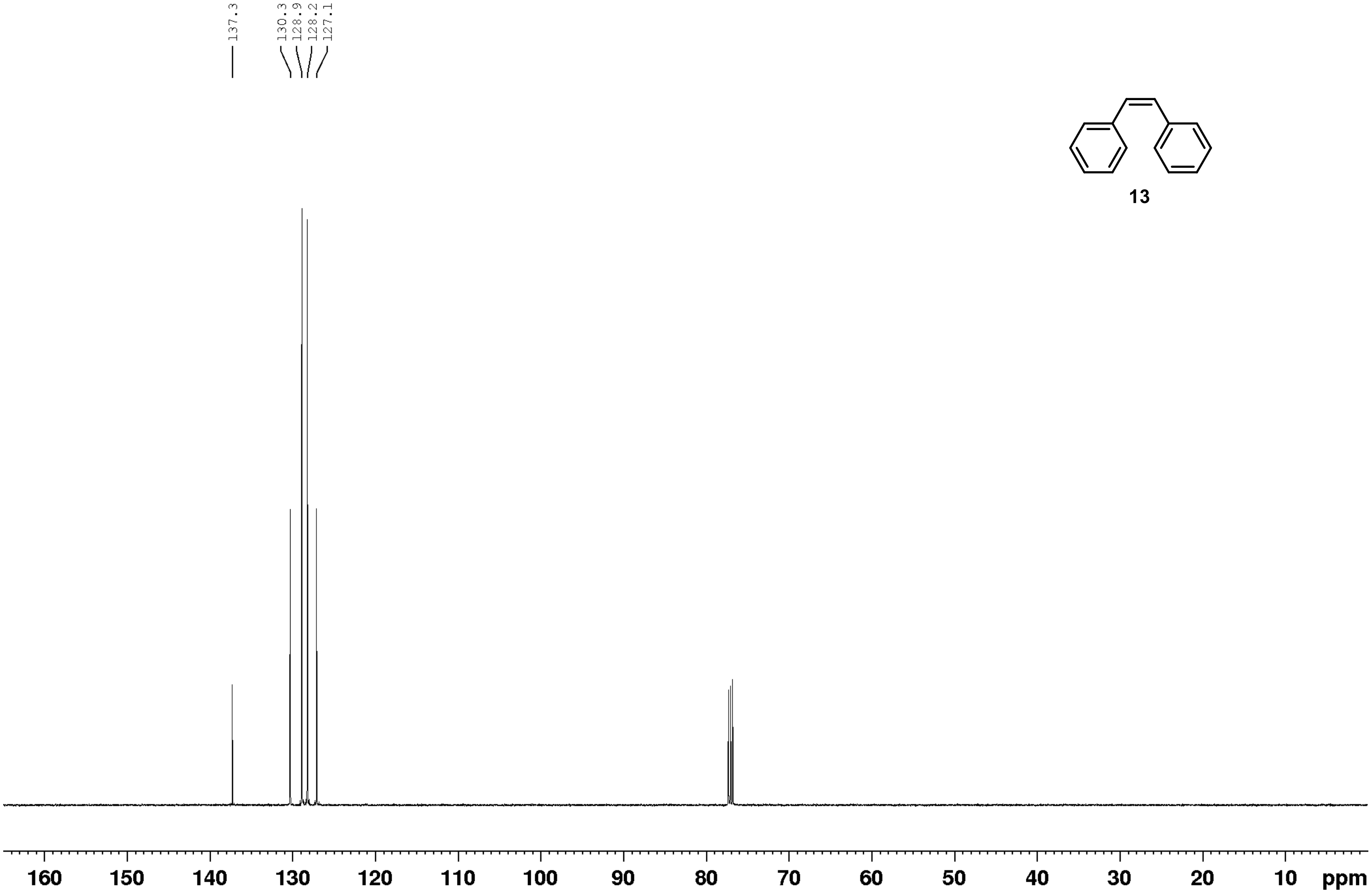
137.3
130.3
128.9
128.2
127.1
160
150
140
130
120
110
100
90
80
70
60
50
40
30
20
10
ppm

13

^{1}H-^{1}H COSY

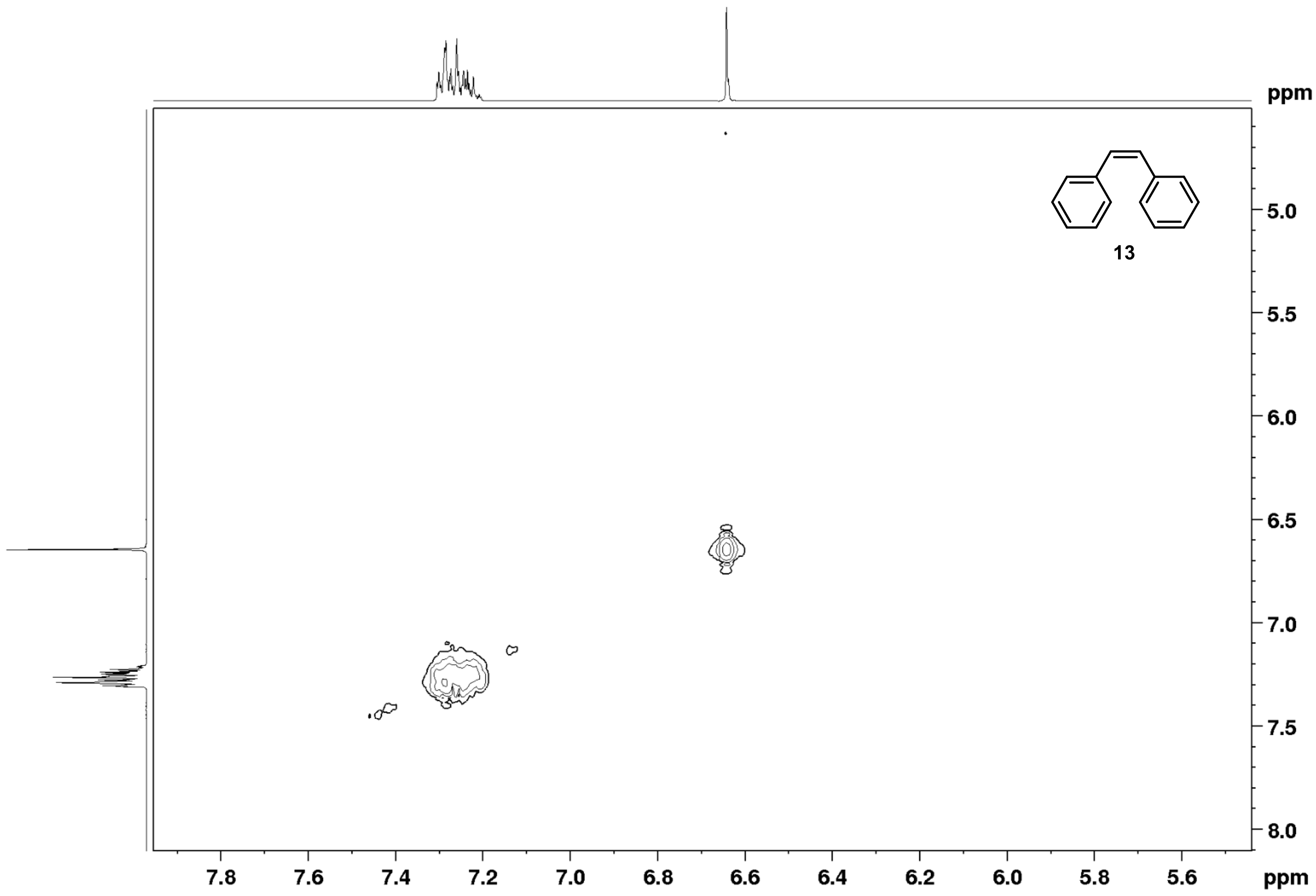

^{1}H-^{13}C HMBC

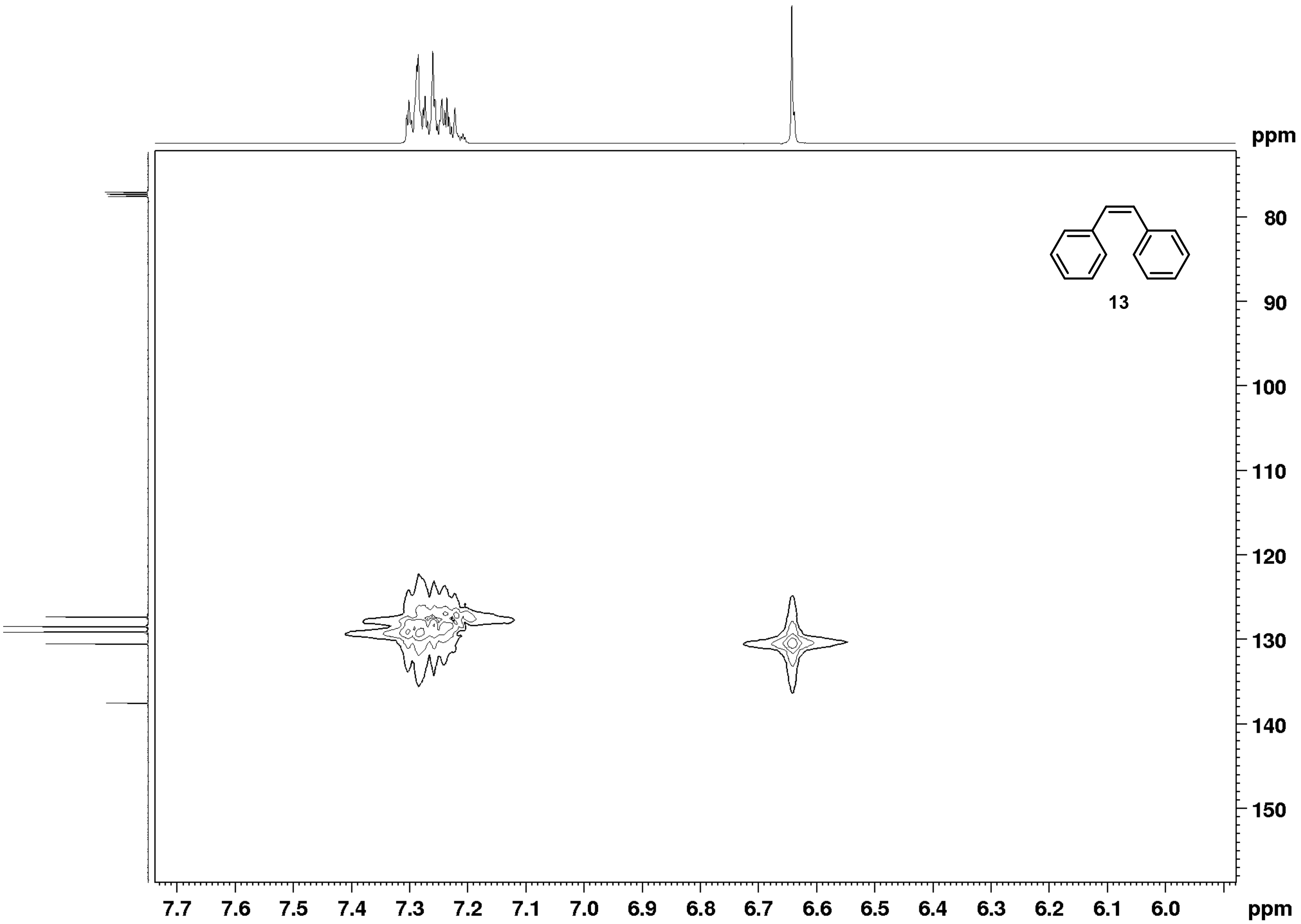
ppm
80
90
100
110
120
130
140
150
13
7.7 7.6 7.5 7.4 7.3 7.2 7.1 7.0 6.9 6.8 6.7 6.6 6.5 6.4 6.3 6.2 6.1 6.0
ppm

^{1}H-^{13}C HSQC

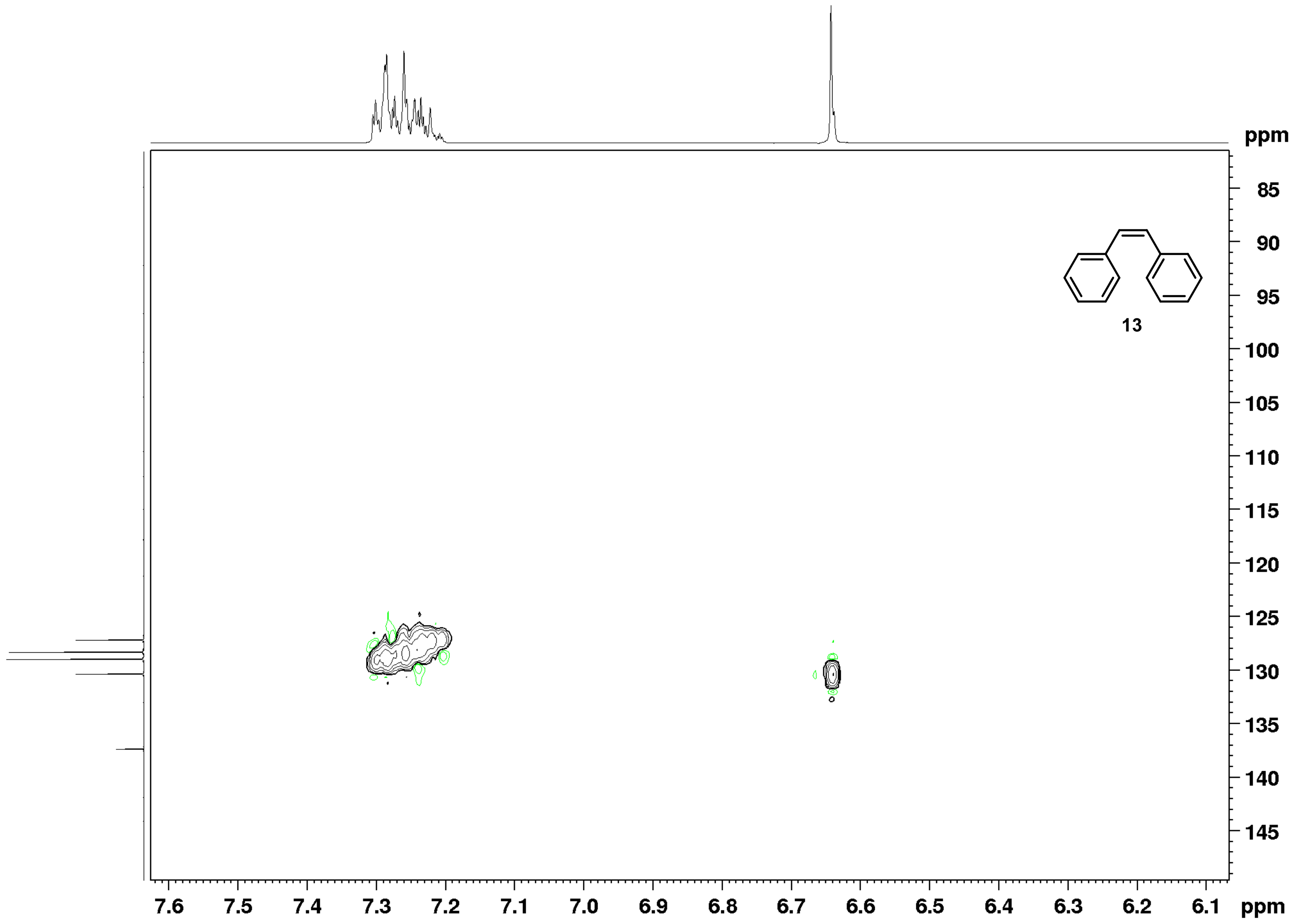

ppm
85
90
95
100
105
110
115
120
125
130
135
140
145
13
7.6
7.5
7.4
7.3
7.2
7.1
7.0
6.9
6.8
6.7
6.6
6.5
6.4
6.3
6.2
6.1
ppm

HRMS

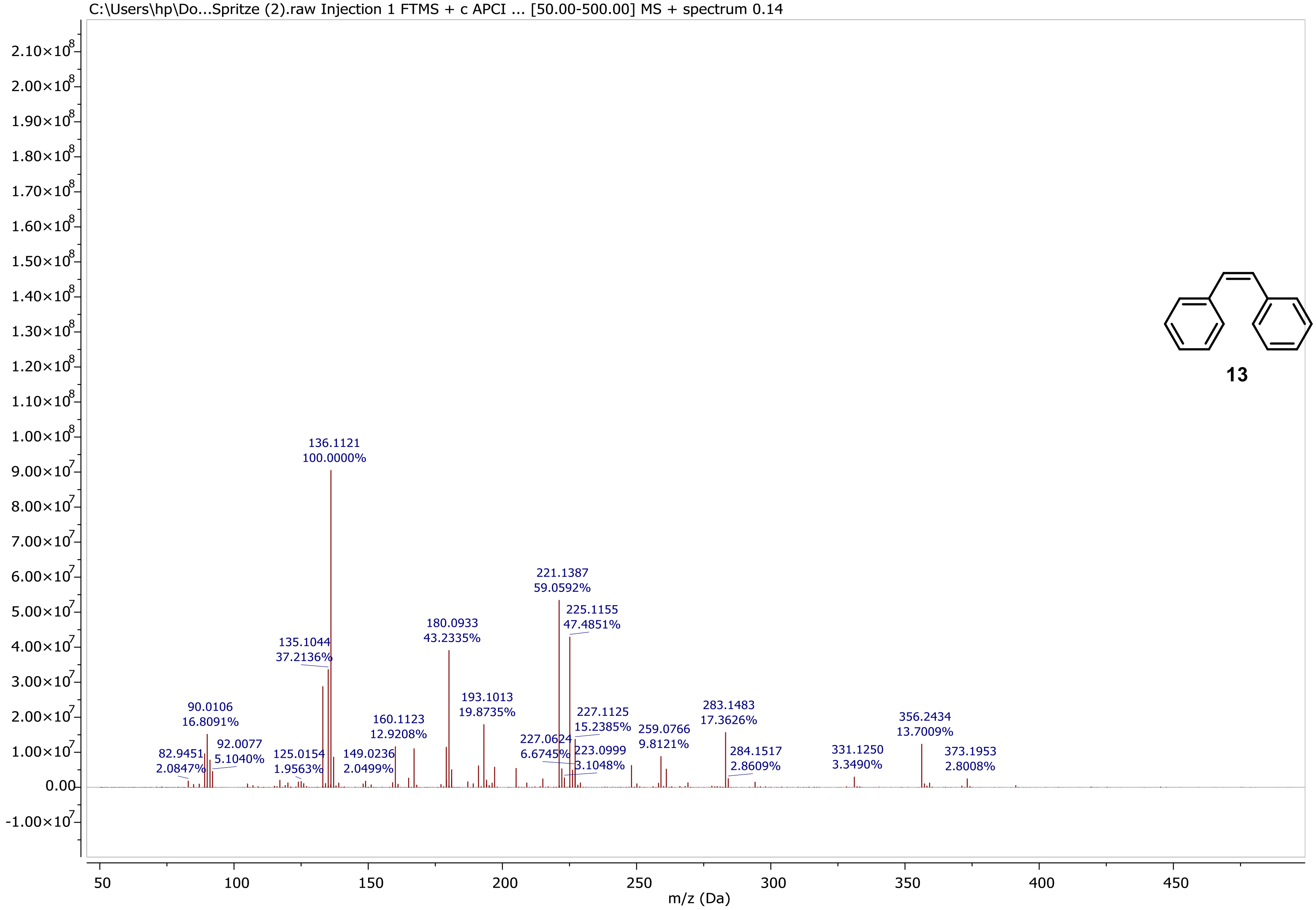
C:\Users\hp\Do...Spritze (2).raw Injection 1 FTMS + c APCI ... [50.00-500.00] MS + spectrum 0.14
136.1121
100.0000%
221.1387
59.0592%
225.1155
47.4851%
180.0933
43.2335%
135.1044
37.2136%
193.1013
19.8735%
283.1483
17.3626%
90.0106
16.8091%
227.1125
15.2385%
356.2434
13.7009%
160.1123
12.9208%
259.0766
9.8121%
227.0624
6.6745%
92.0077
5.1040%
223.0999
3.1048%
331.1250
3.3490%
284.1517
2.8609%
373.1953
2.8008%
82.9451
2.0847%
125.0154
1.9563%
149.0236
2.0499%
m/z (Da)

13

1-Phenylethan-1-ol (16)

^{1}H NMR

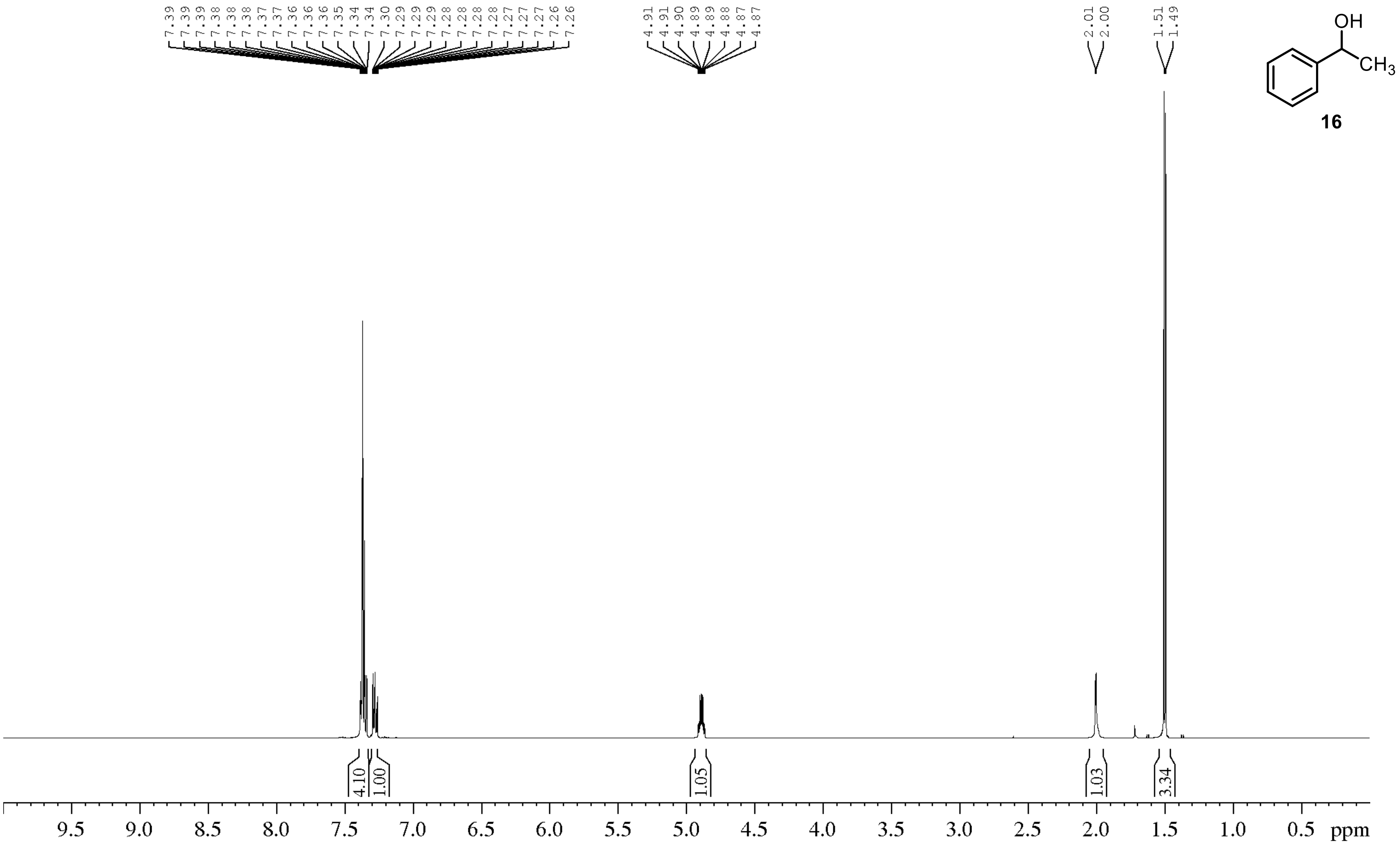

16

^{13}C NMR

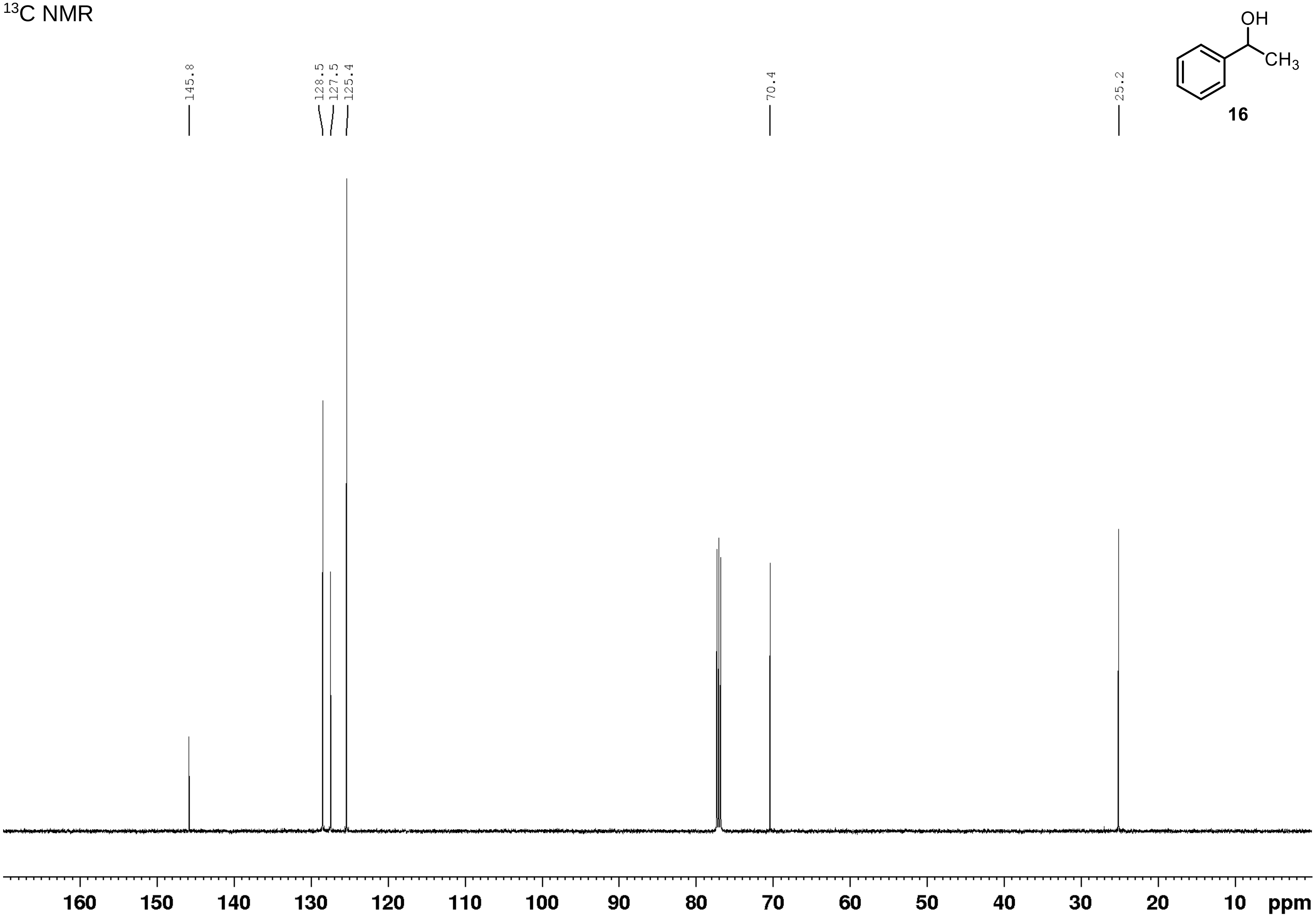

16

^1H-^1H COSY

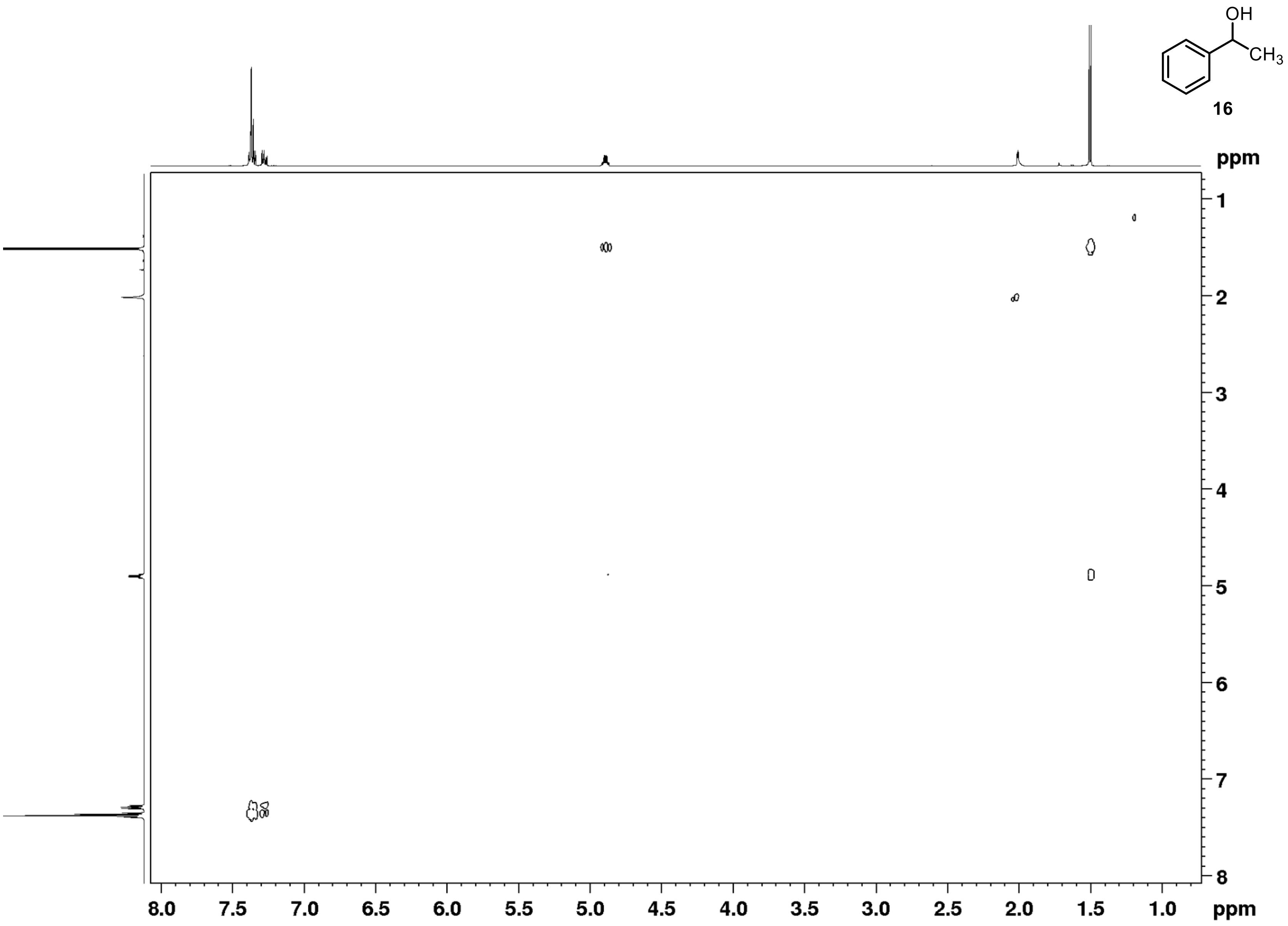

OH
CH3
16
ppm
1
2
3
4
5
6
7
8
8.0
7.5
7.0
6.5
6.0
5.5
5.0
4.5
4.0
3.5
3.0
2.5
2.0
1.5
1.0
ppm

^1H-^{13}C HMBC

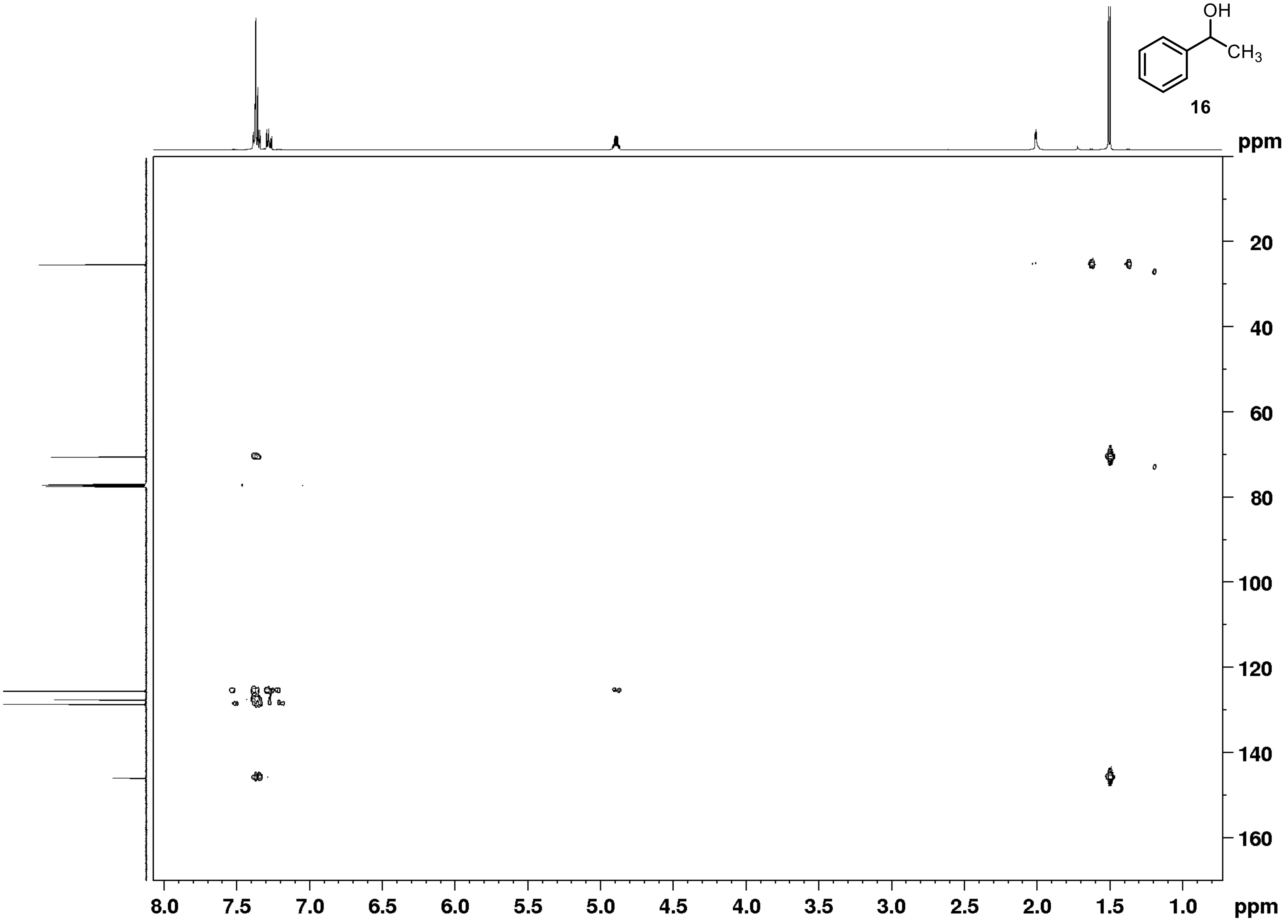
OH
CH3
16
ppm
20
40
60
80
100
120
140
160
8.0 7.5 7.0 6.5 6.0 5.5 5.0 4.5 4.0 3.5 3.0 2.5 2.0 1.5 1.0
ppm

^1H-^{13}C HSQC

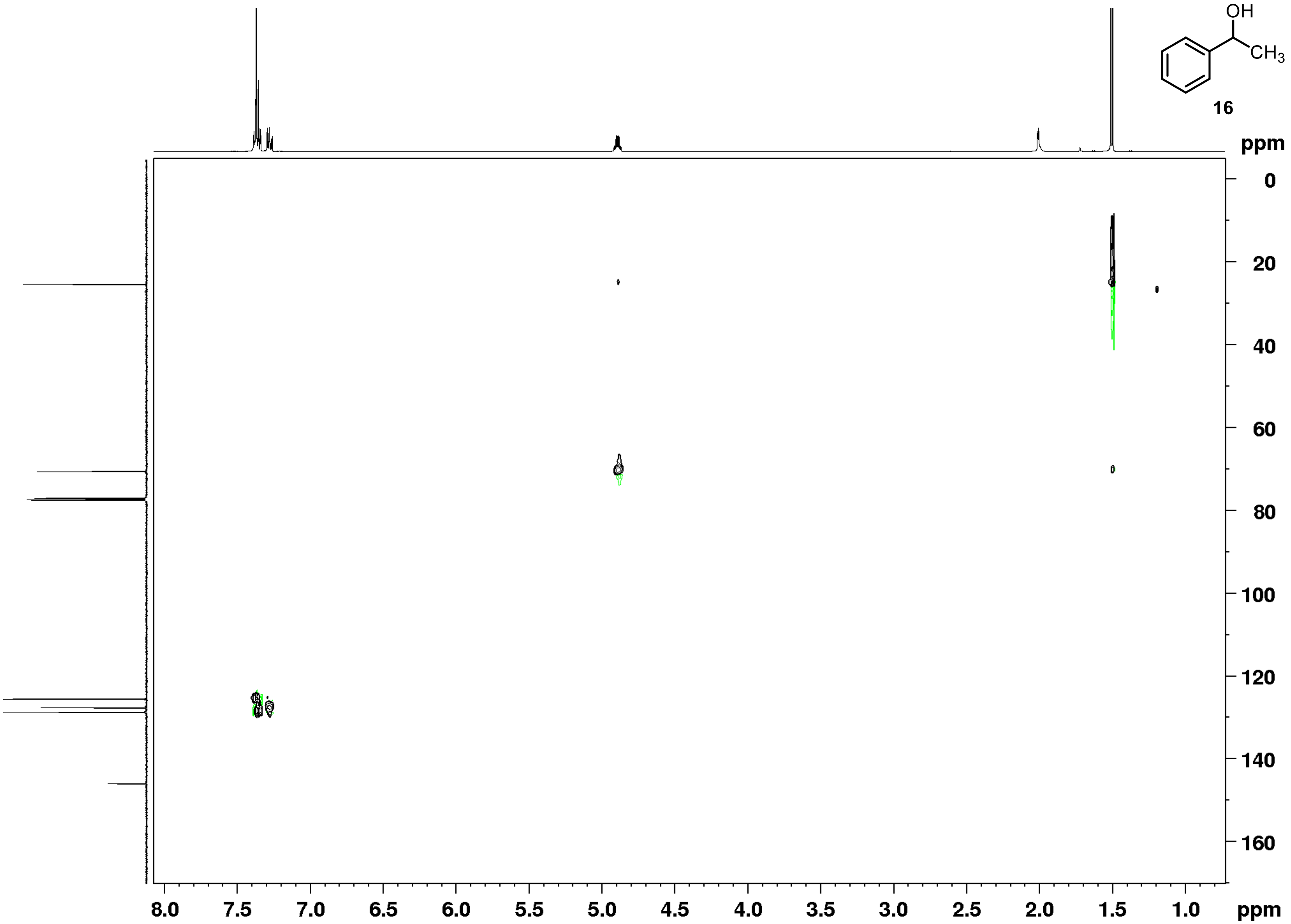

16

HRMS

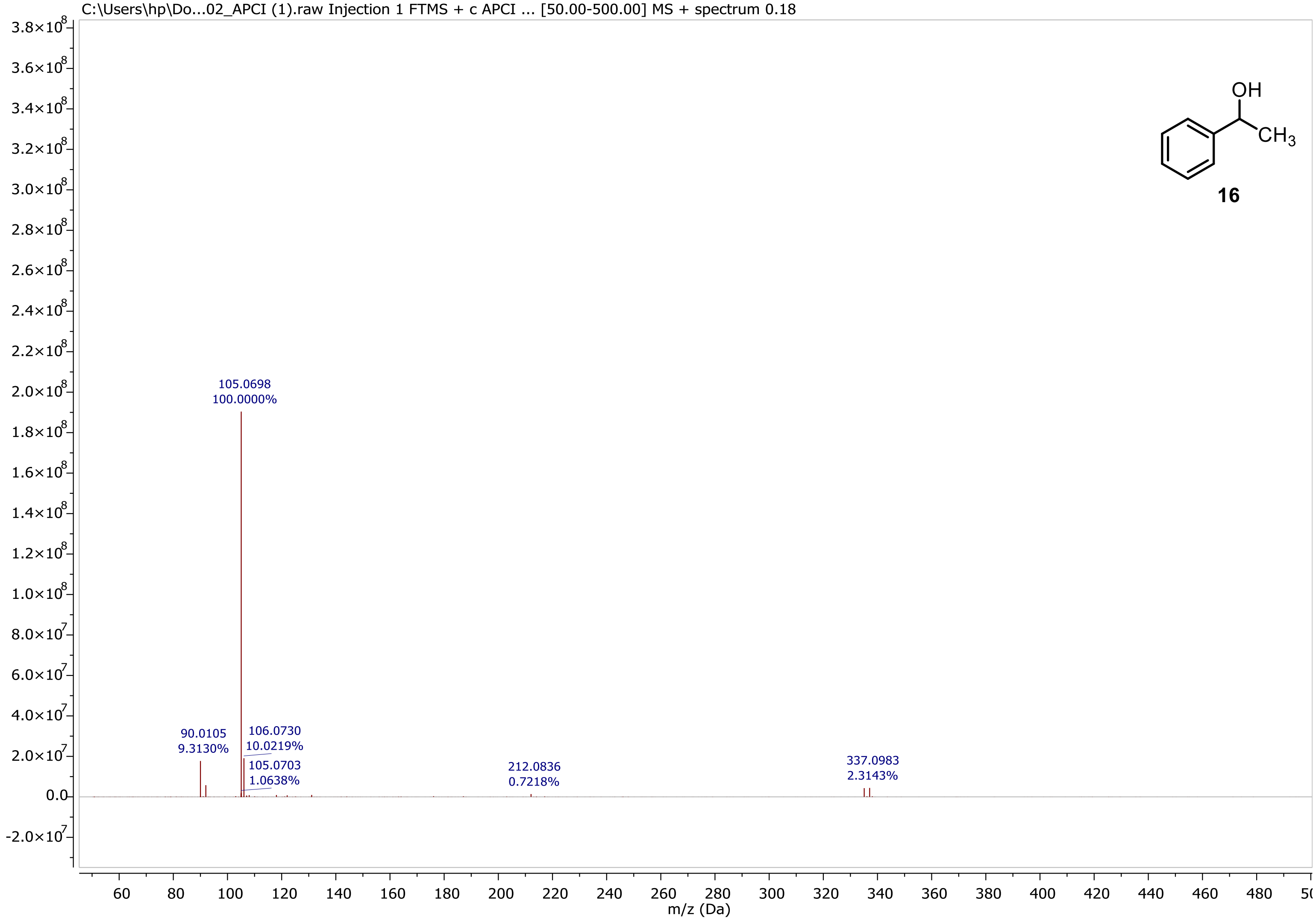

C:\Users\hp\Do...02_APCI (1).raw Injection 1 FTMS + c APCI ... [50.00-500.00] MS + spectrum 0.18
105.0698
100.0000%
90.0105
9.3130%
106.0730
10.0219%
105.0703
1.0638%
212.0836
0.7218%
337.0983
2.3143%
m/z (Da)
OH
CH3

16

Benzyl alcohol (9)

^{1}H NMR

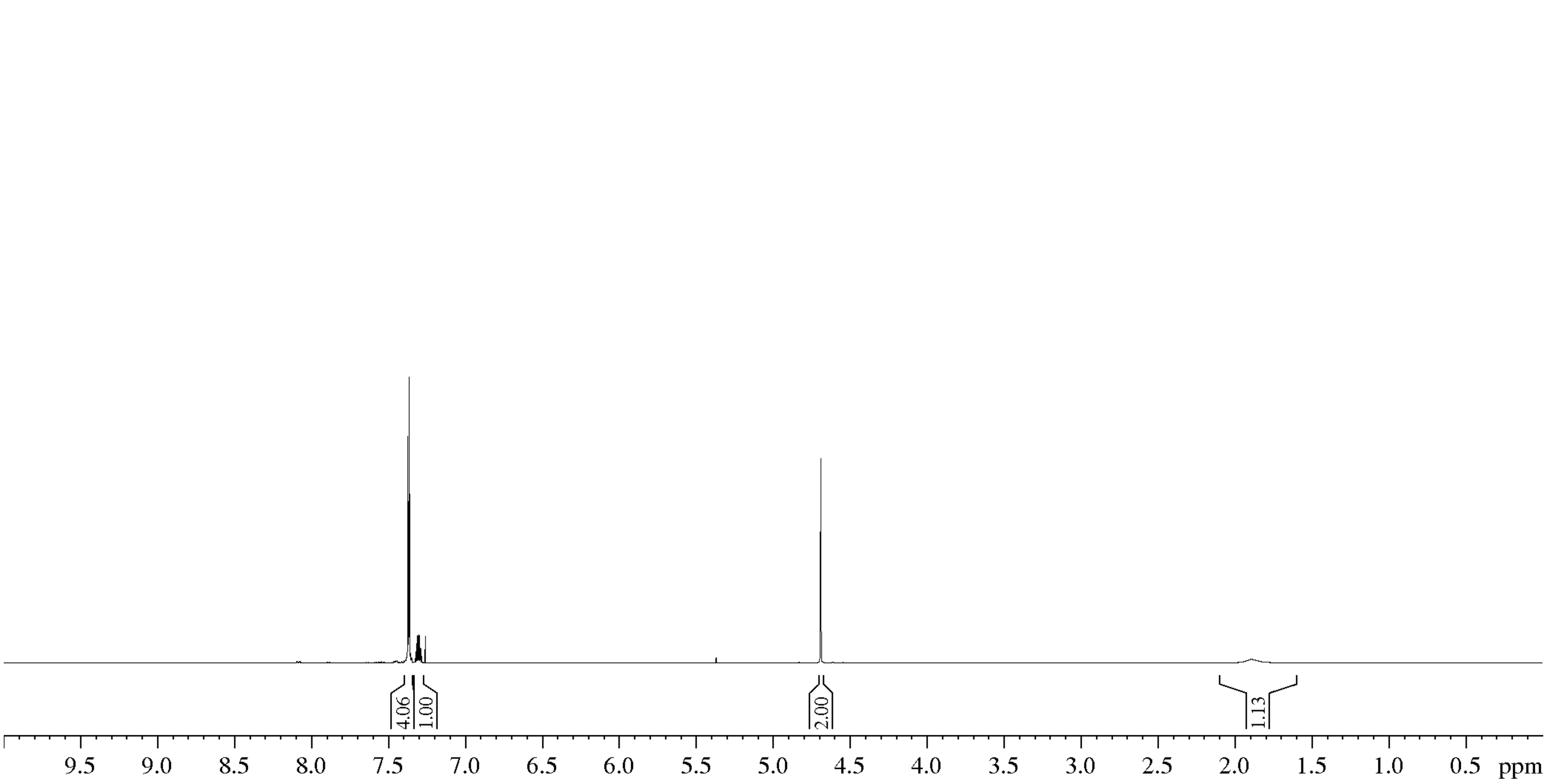

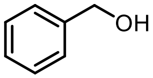

9

^{13}C NMR

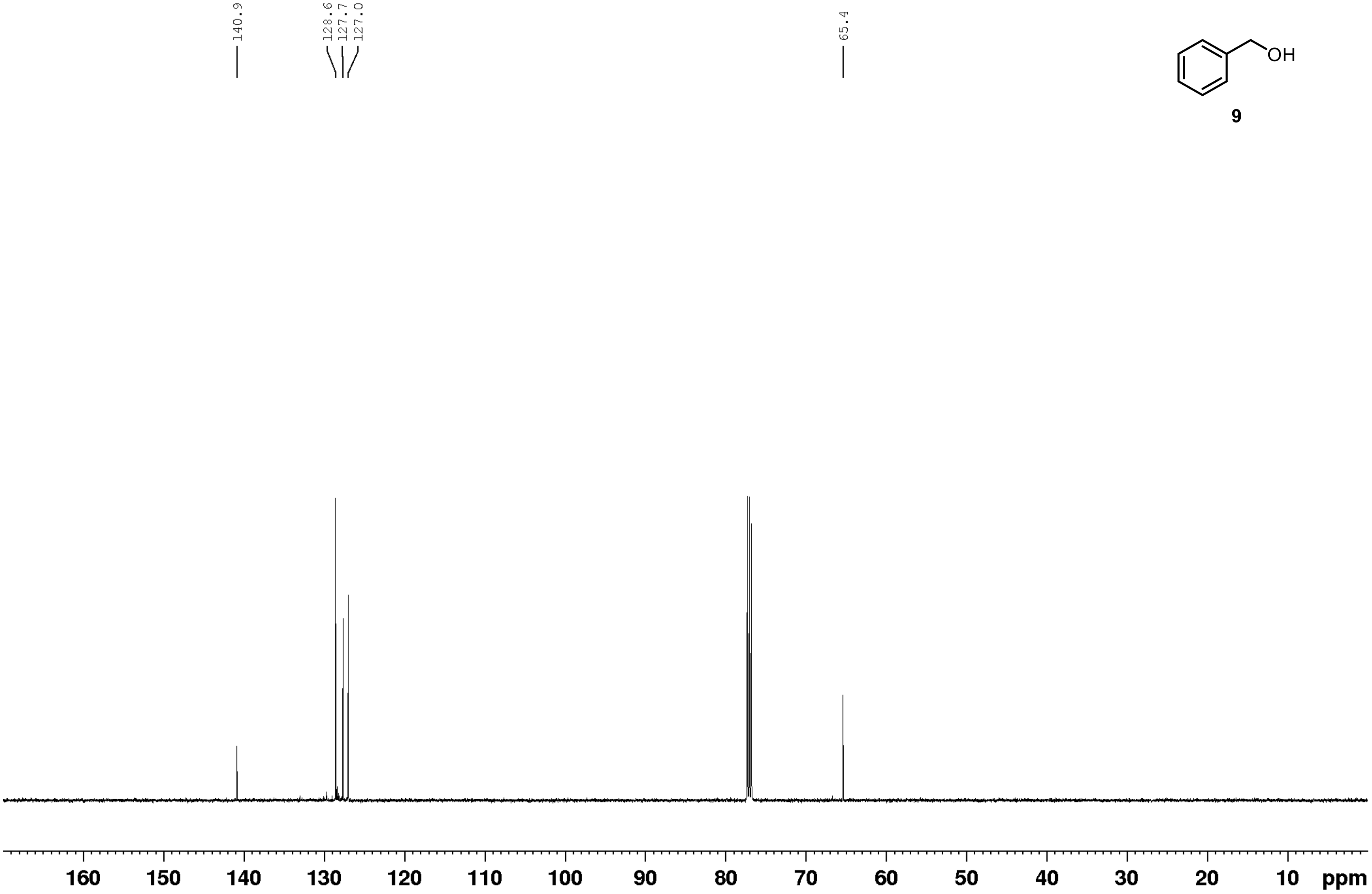

140.9
128.6
127.7
127.0
65.4
OH
160
150
140
130
120
110
100
90
80
70
60
50
40
30
20
10
ppm

9

^1H-^1H COSY

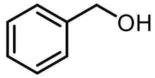

9

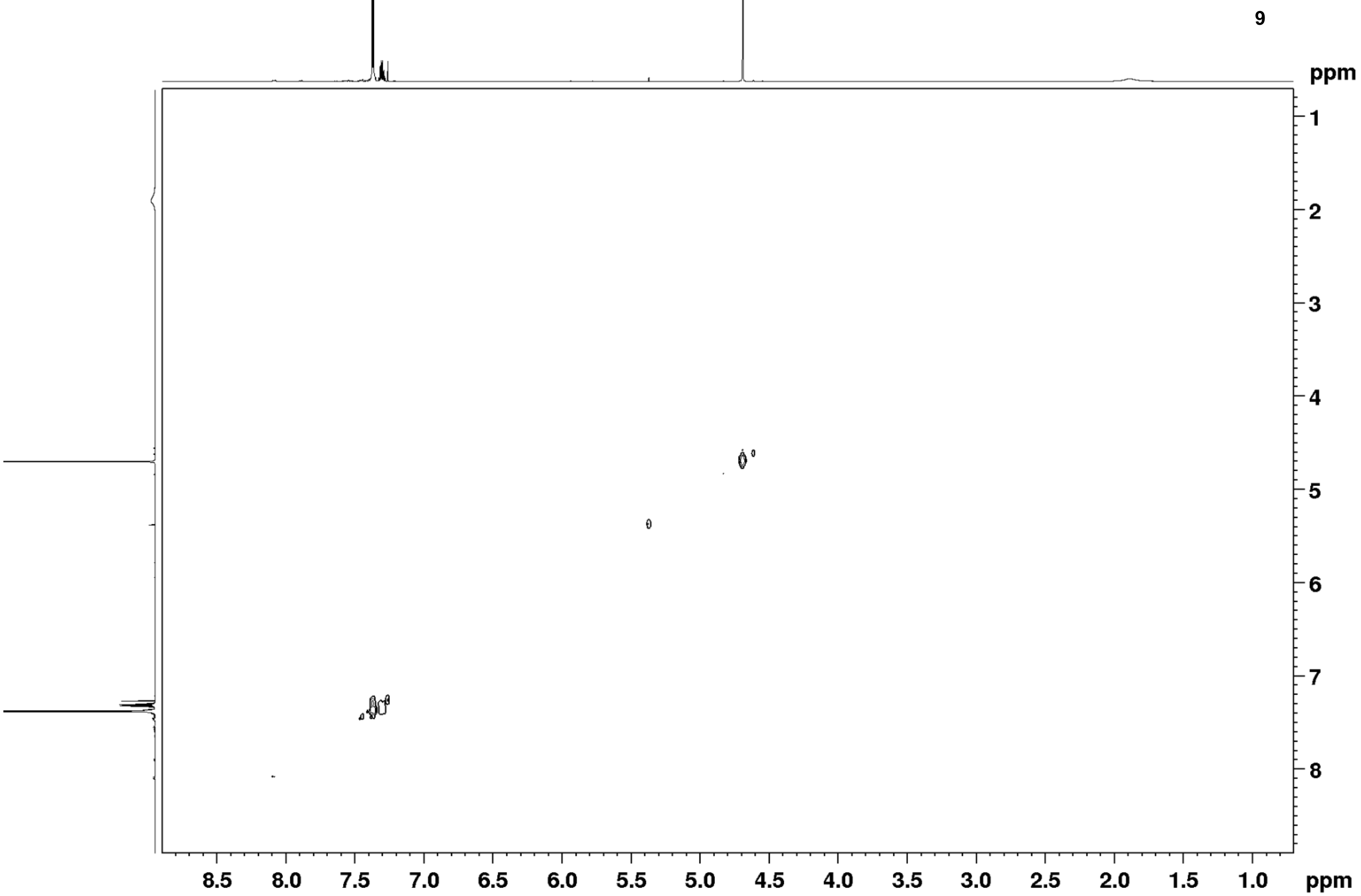

^{1}H-^{13}C HMBC

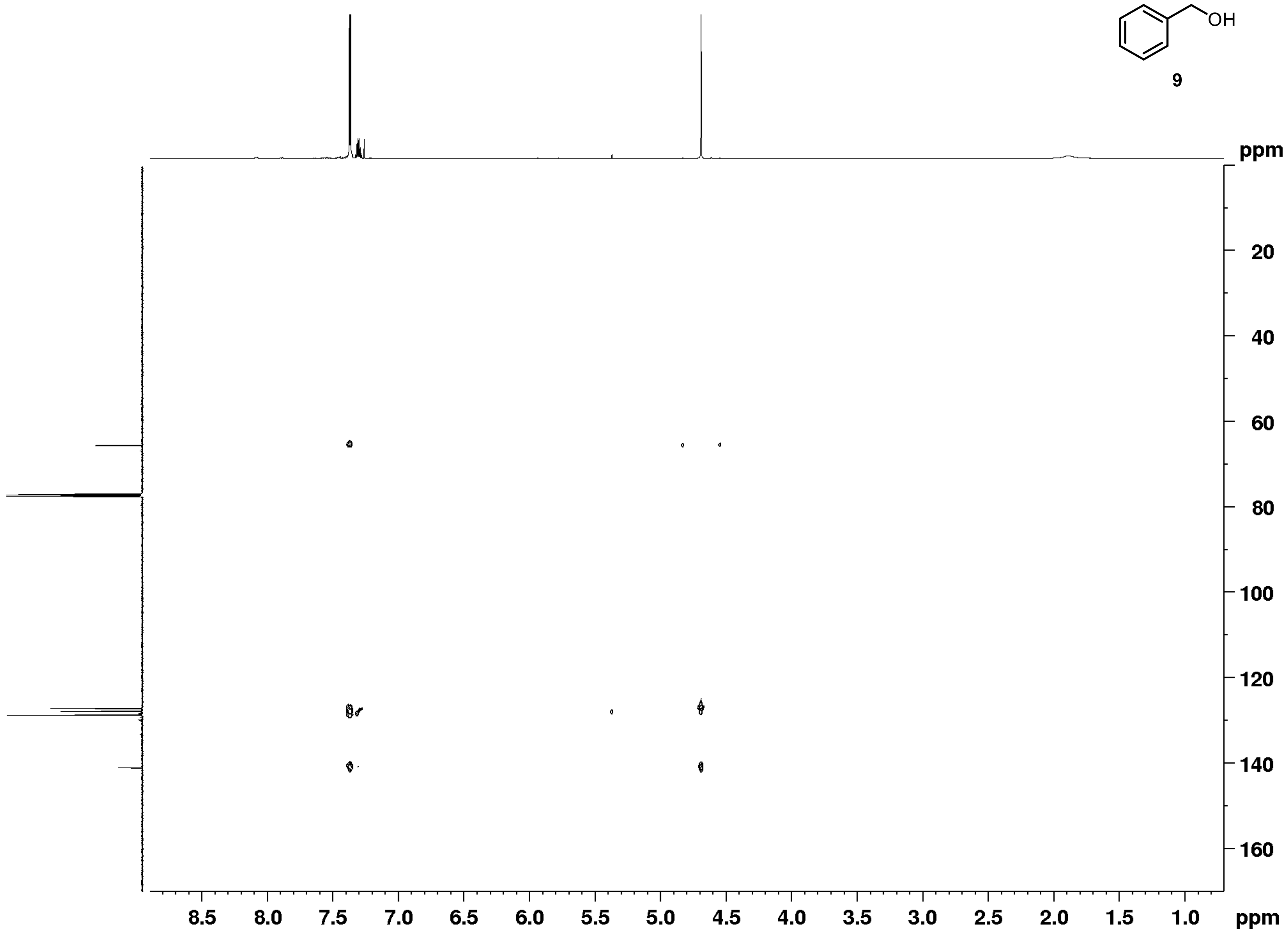

OH
9
ppm
20
40
60
80
100
120
140
160
8.5
8.0
7.5
7.0
6.5
6.0
5.5
5.0
4.5
4.0
3.5
3.0
2.5
2.0
1.5
1.0
ppm

^{1}H-^{13}C HSQC

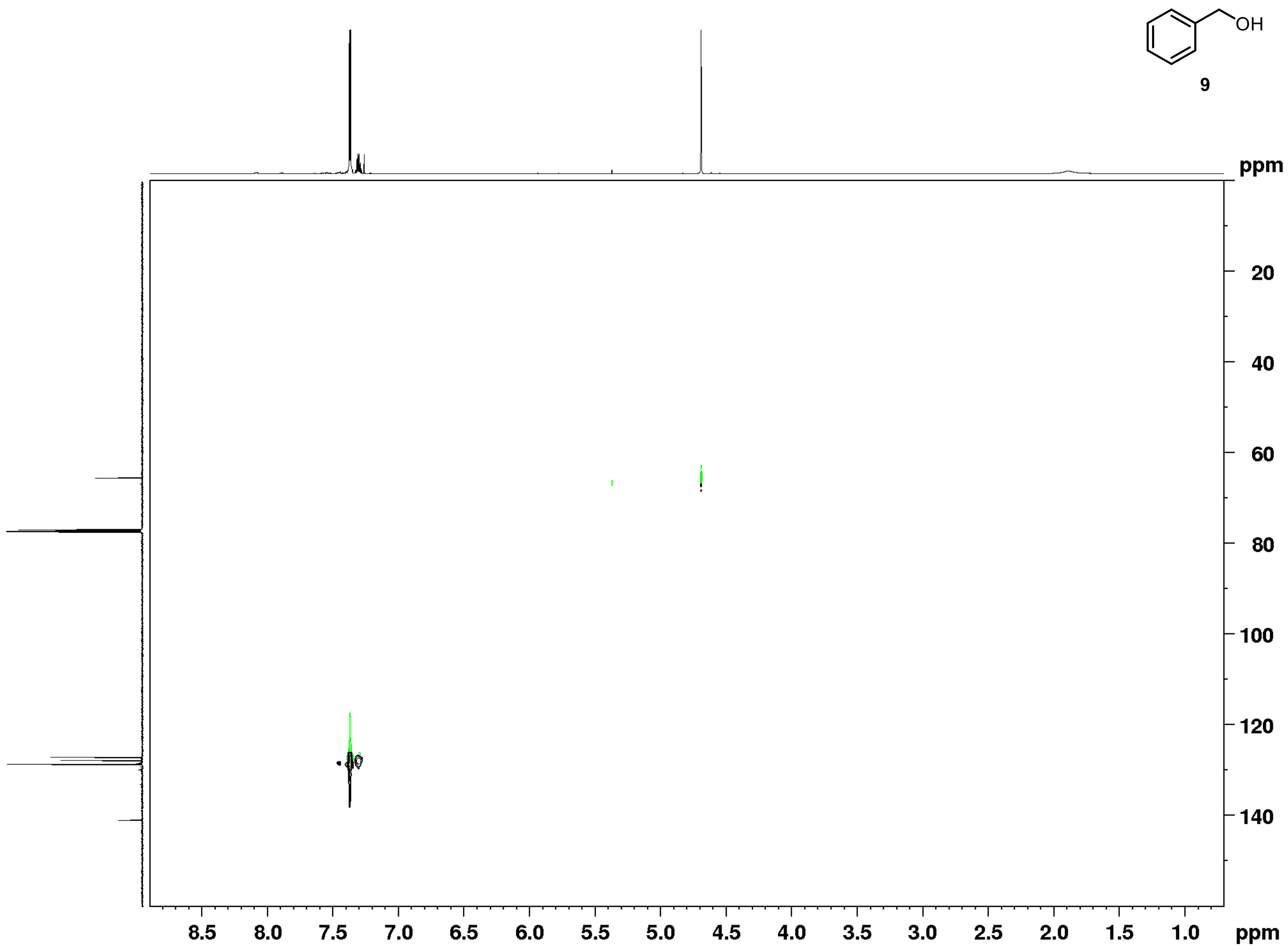

OH
9
ppm
20
40
60
80
100
120
140
8.5
8.0
7.5
7.0
6.5
6.0
5.5
5.0
4.5
4.0
3.5
3.0
2.5
2.0
1.5
1.0
ppm

HRMS

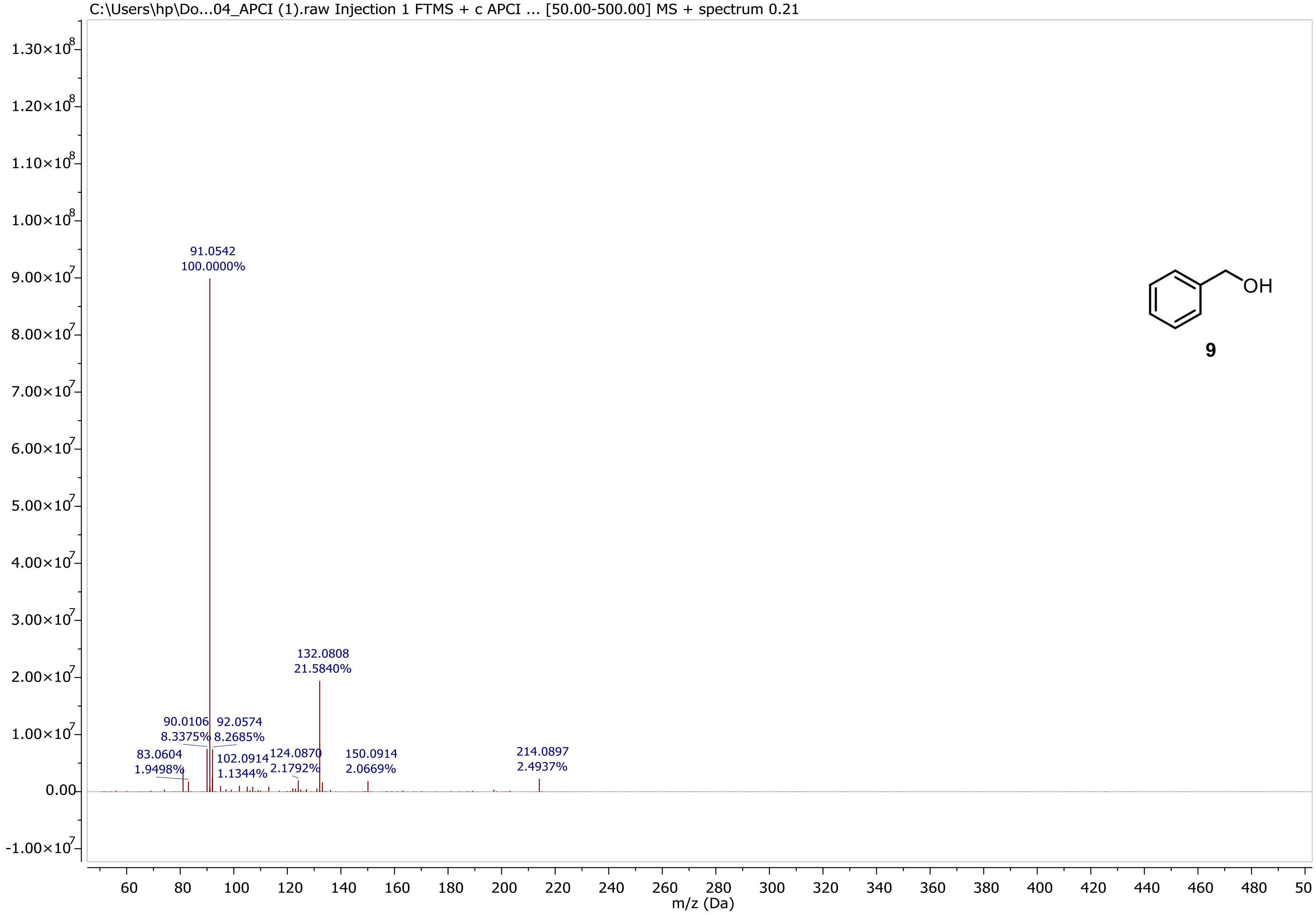
C:\Users\hp\Do...04_APCI (1).raw Injection 1 FTMS + c APCI ... [50.00-500.00] MS + spectrum 0.21
91.0542
100.0000%
132.0808
21.5840%
90.0106
8.3375%
92.0574
8.2685%
83.0604
1.9498%
102.0914
1.1344%
124.0870
2.1792%
150.0914
2.0669%
214.0897
2.4937%
m/z (Da)
OH

9

Benzyl alcohol (9)

^{1}H NMR

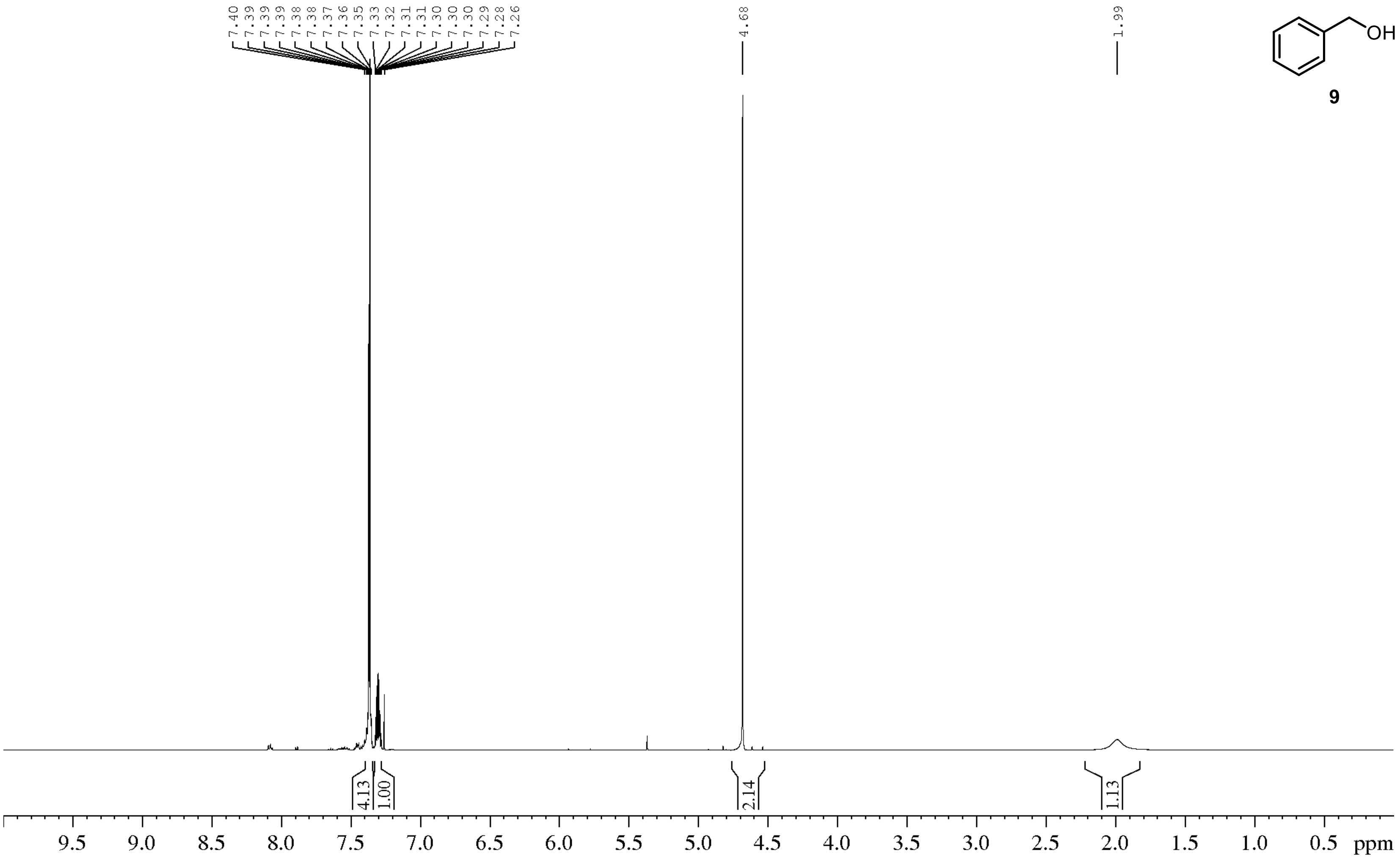

^{13}C NMR

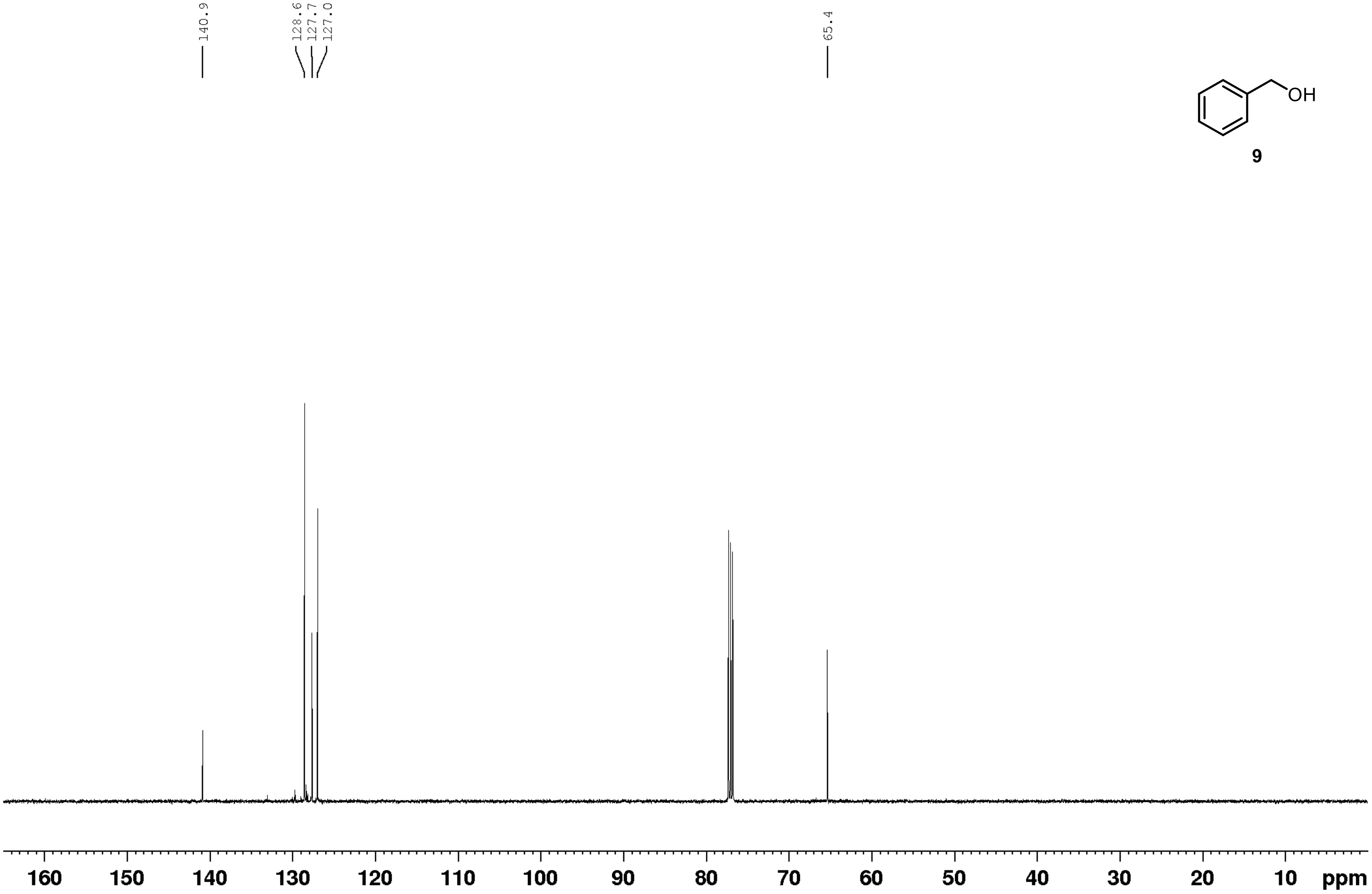

9

^1H-^1H COSY

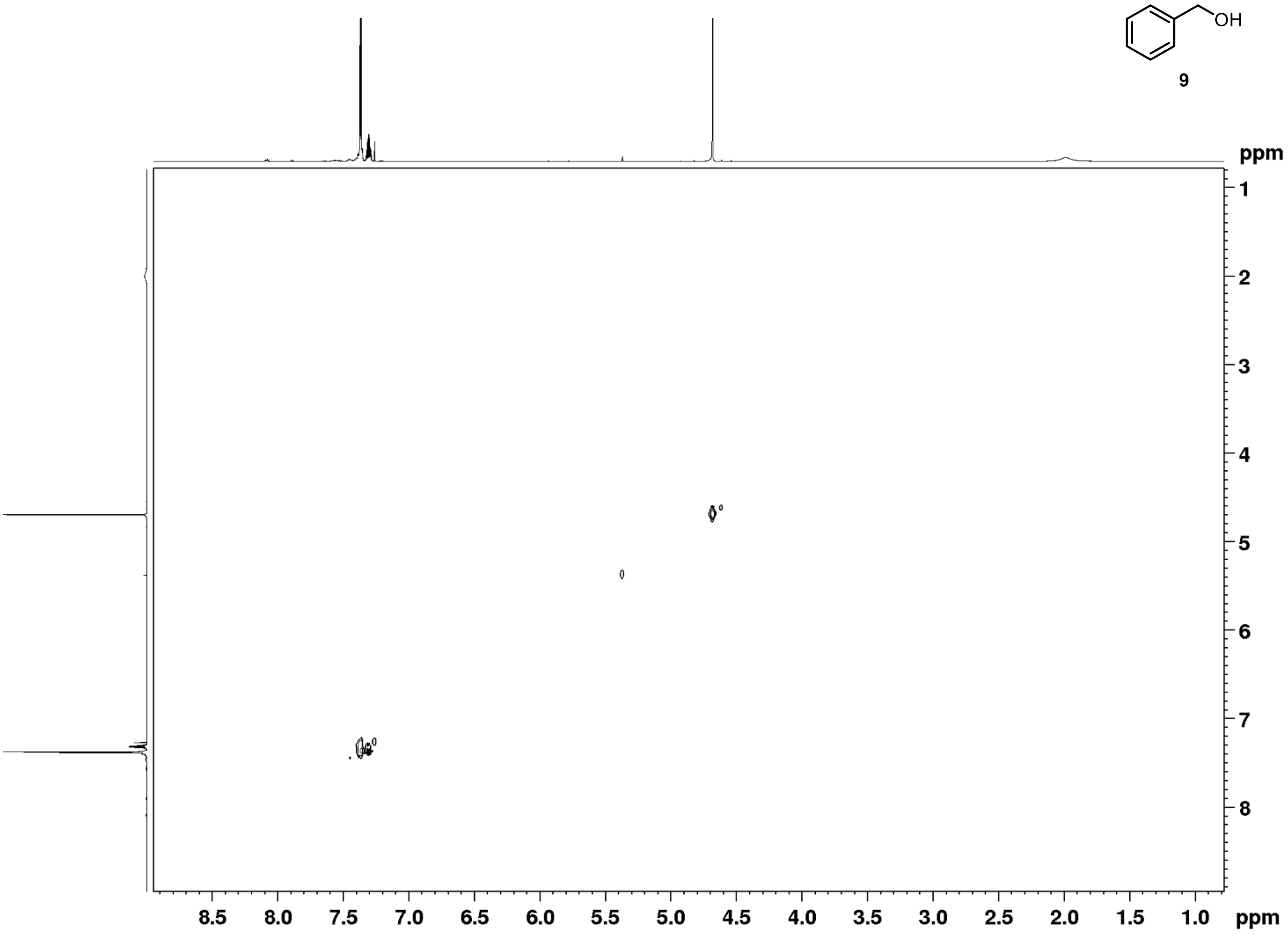

^{1}H-^{13}C HMBC

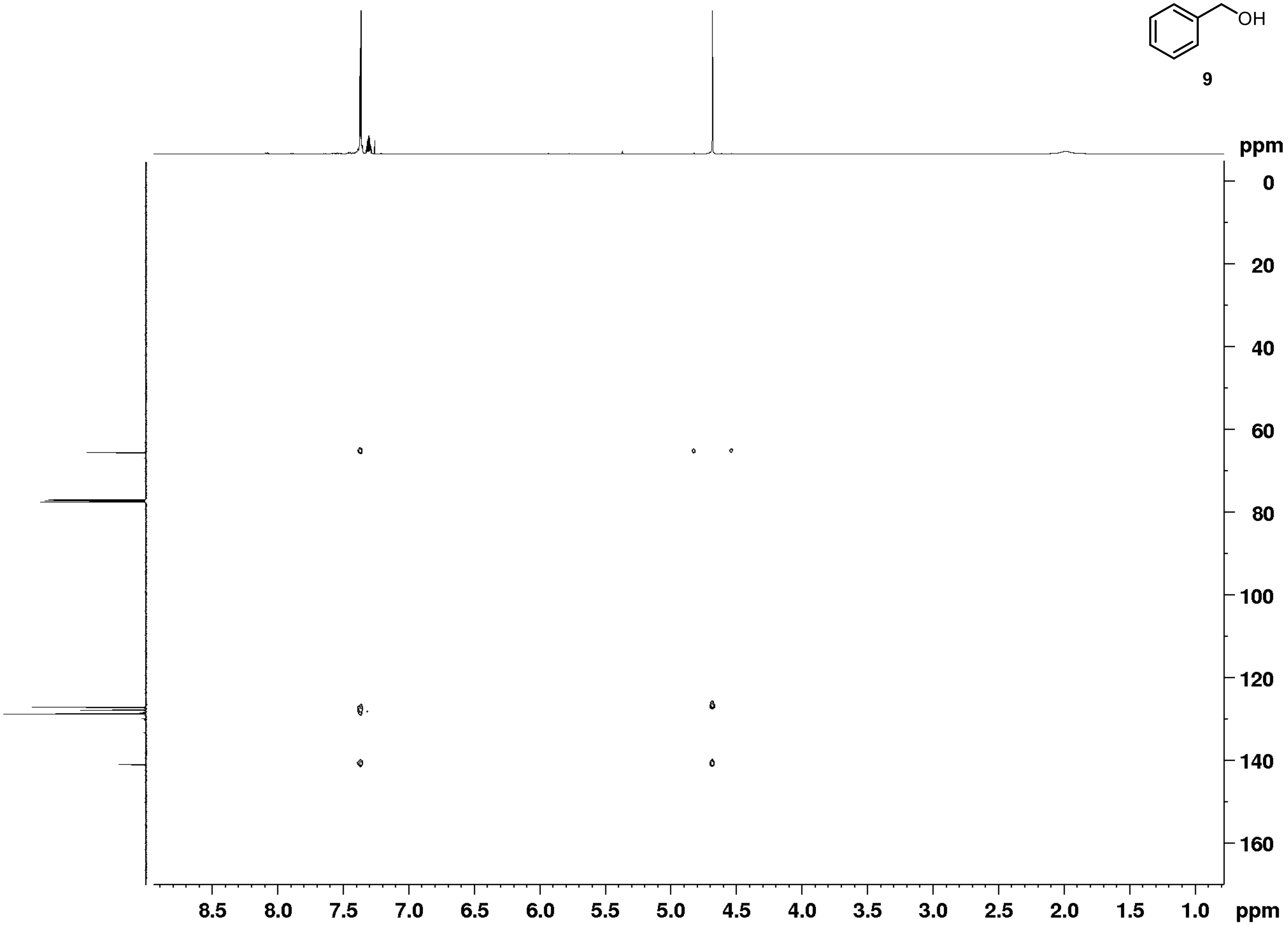

OH
ppm
0
20
40
60
80
100
120
140
160
8.5
8.0
7.5
7.0
6.5
6.0
5.5
5.0
4.5
4.0
3.5
3.0
2.5
2.0
1.5
1.0
ppm

9

^{1}H-^{13}C HSQC

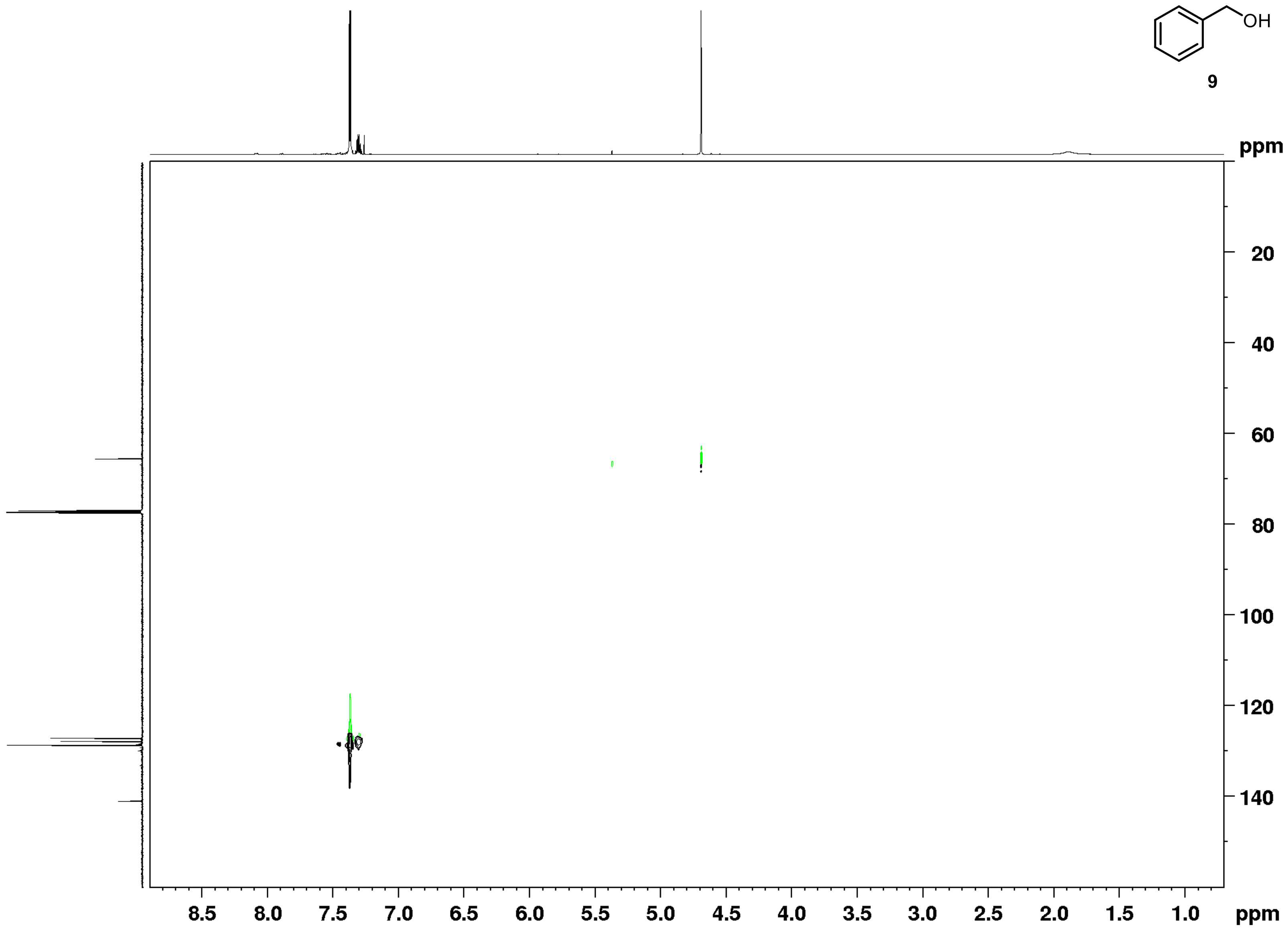
OH
9
ppm
20
40
60
80
100
120
140
8.5
8.0
7.5
7.0
6.5
6.0
5.5
5.0
4.5
4.0
3.5
3.0
2.5
2.0
1.5
1.0
ppm

HRMS

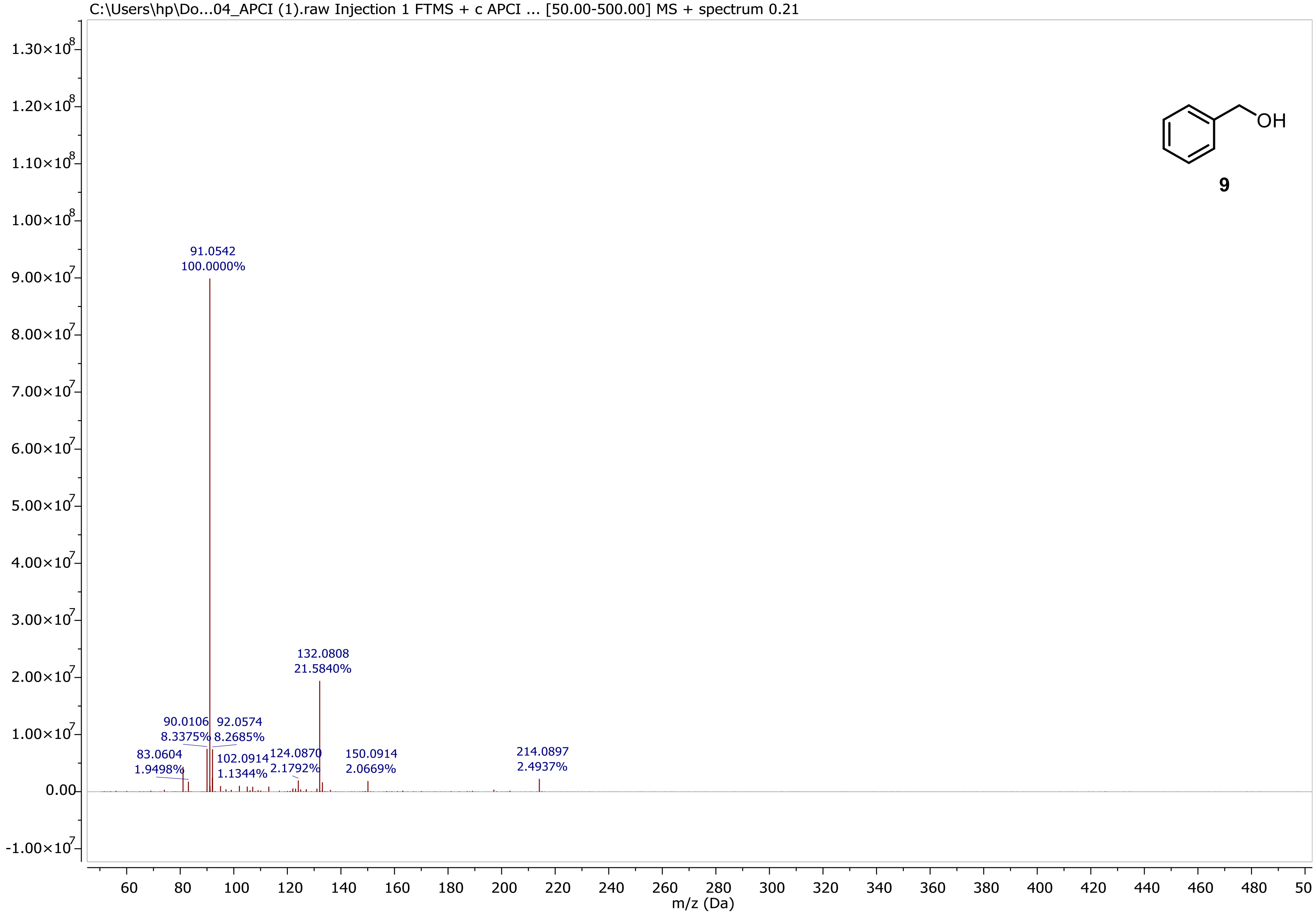

C:\Users\hp\Do...04_APCI (1).raw Injection 1 FTMS + c APCI ... [50.00-500.00] MS + spectrum 0.21
91.0542
100.0000%
132.0808
21.5840%
90.0106
8.3375%
92.0574
8.2685%
83.0604
1.9498%
102.0914
1.1344%
124.0870
2.1792%
150.0914
2.0669%
214.0897
2.4937%
OH
9
m/z (Da)

(4-(1,3-Dioxan-2-yl)phenyl)methanol (11)

^{1}H NMR

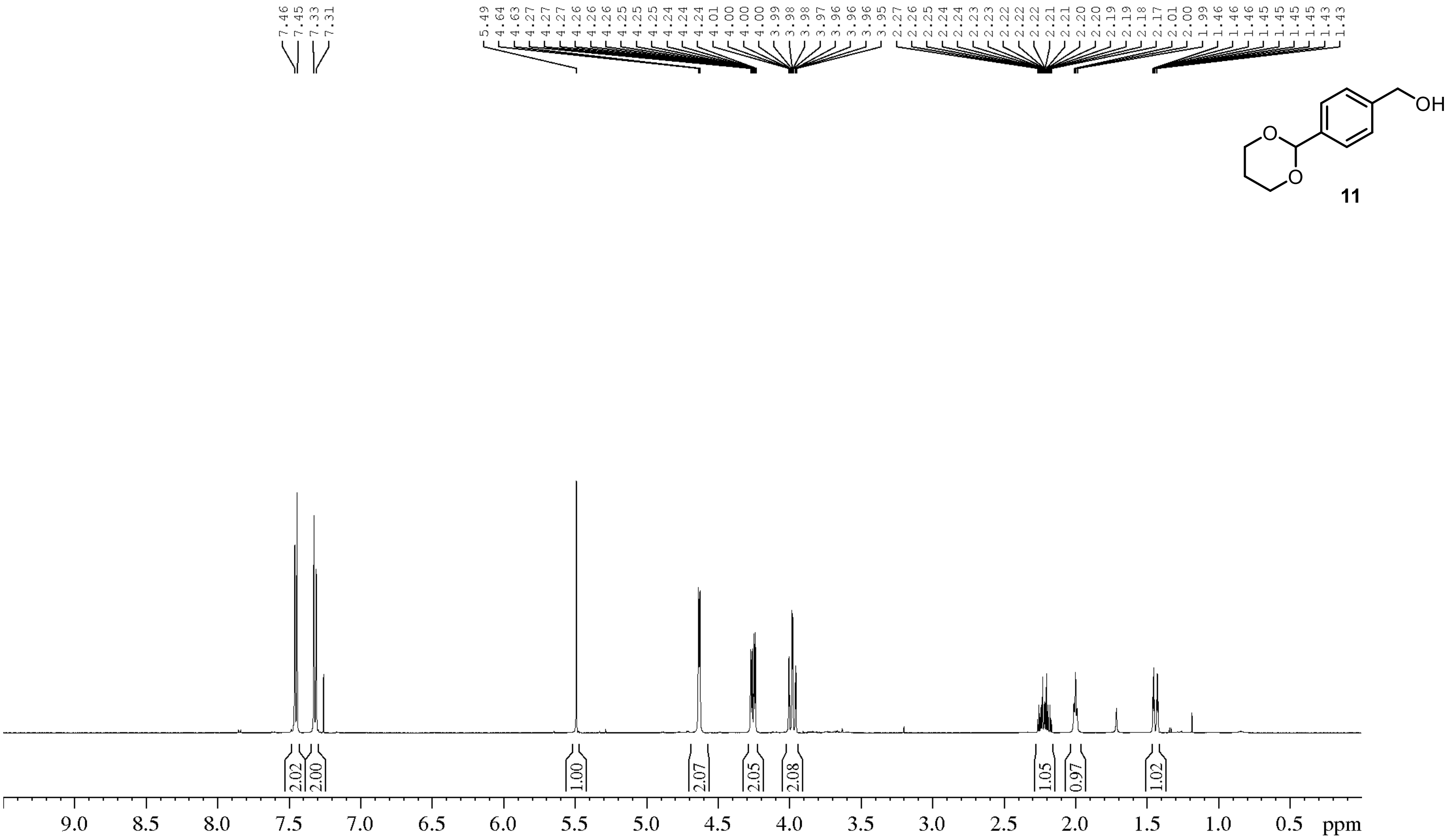

11

^{13}C NMR

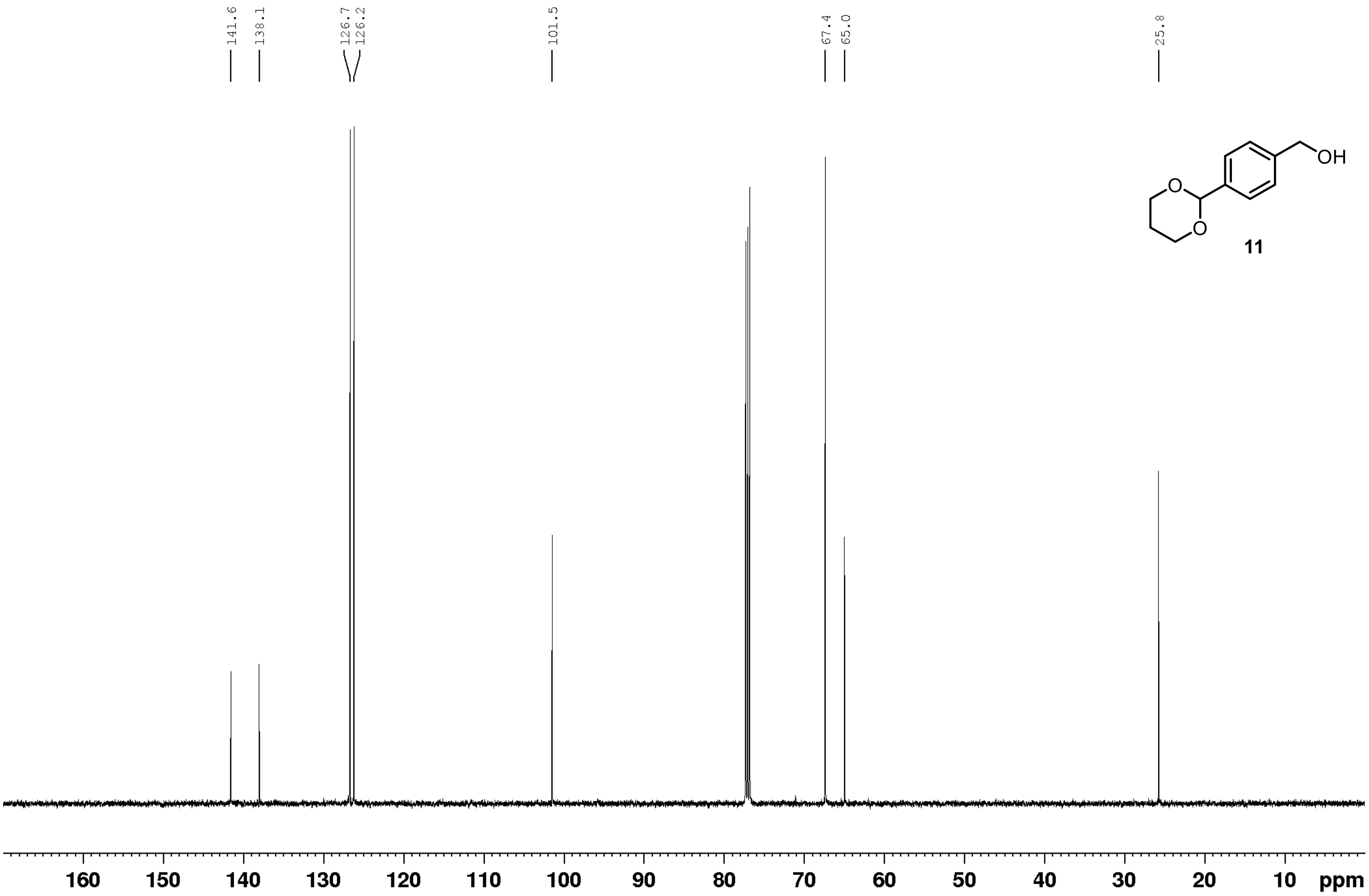

11

^{1}H-^{1}H COSY

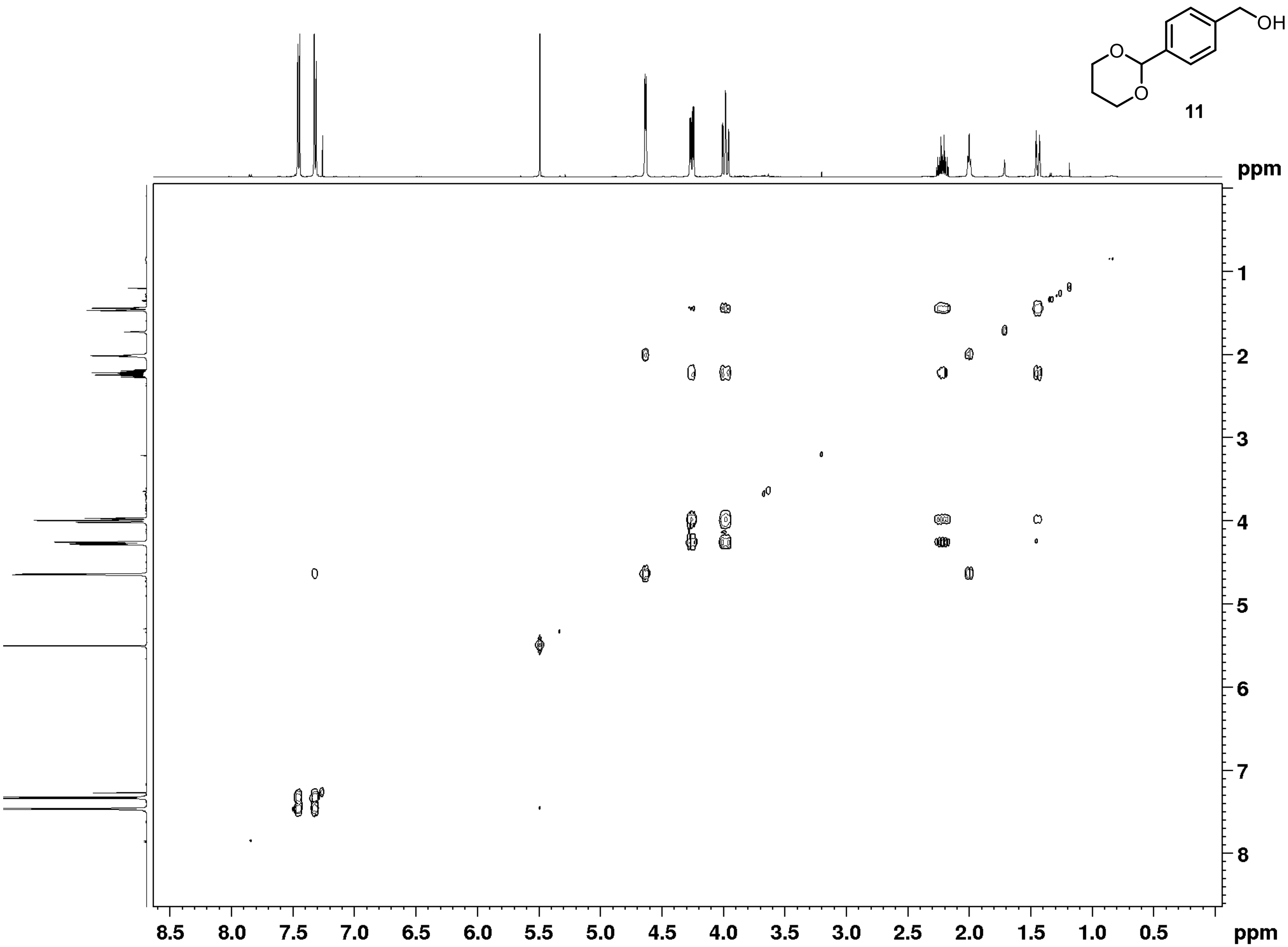

^{1}H-^{13}C HMBC

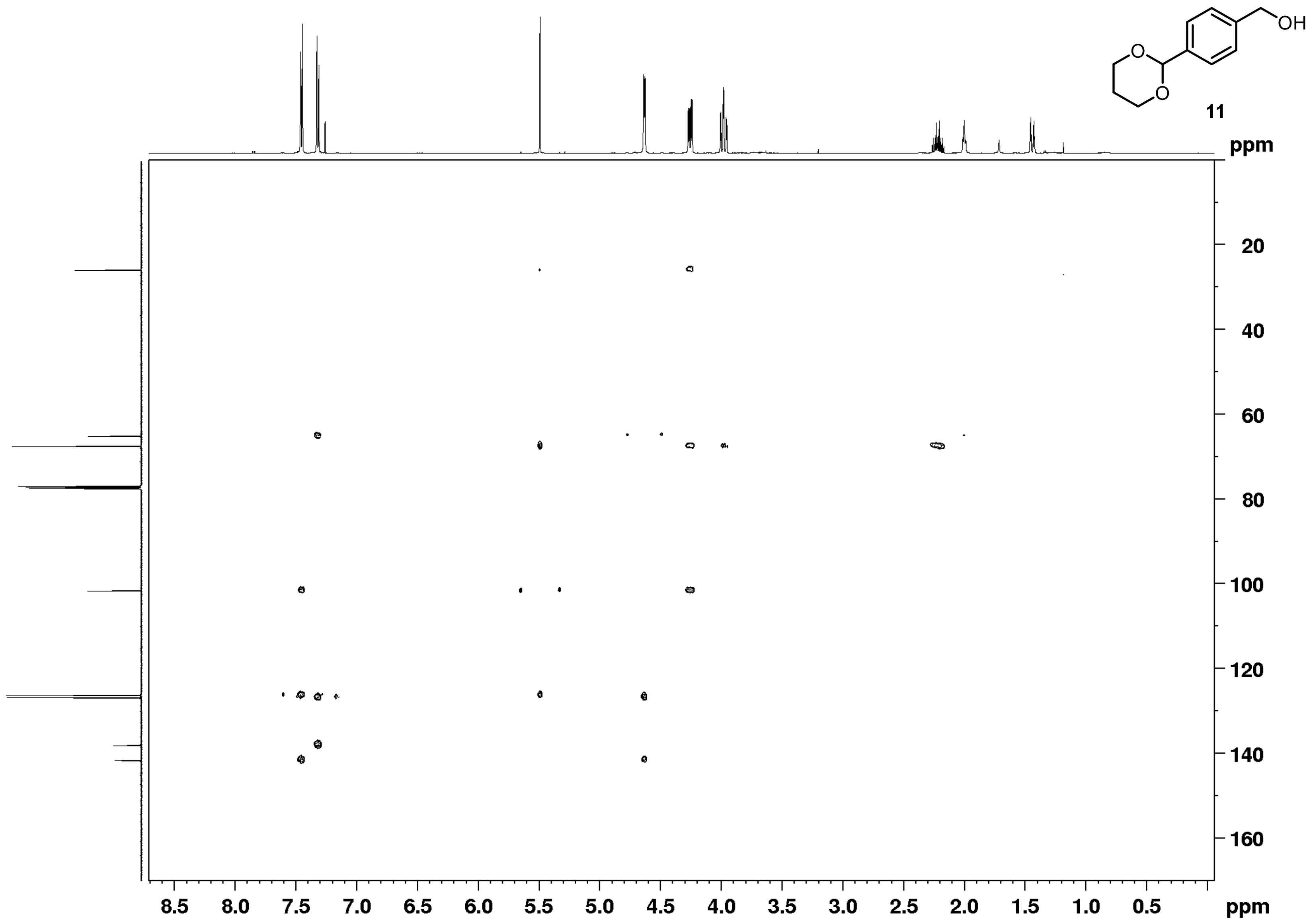

OH
O
O
11
ppm
20
40
60
80
100
120
140
160
8.5
8.0
7.5
7.0
6.5
6.0
5.5
5.0
4.5
4.0
3.5
3.0
2.5
2.0
1.5
1.0
0.5
ppm

^{1}H-^{13}C HSQC

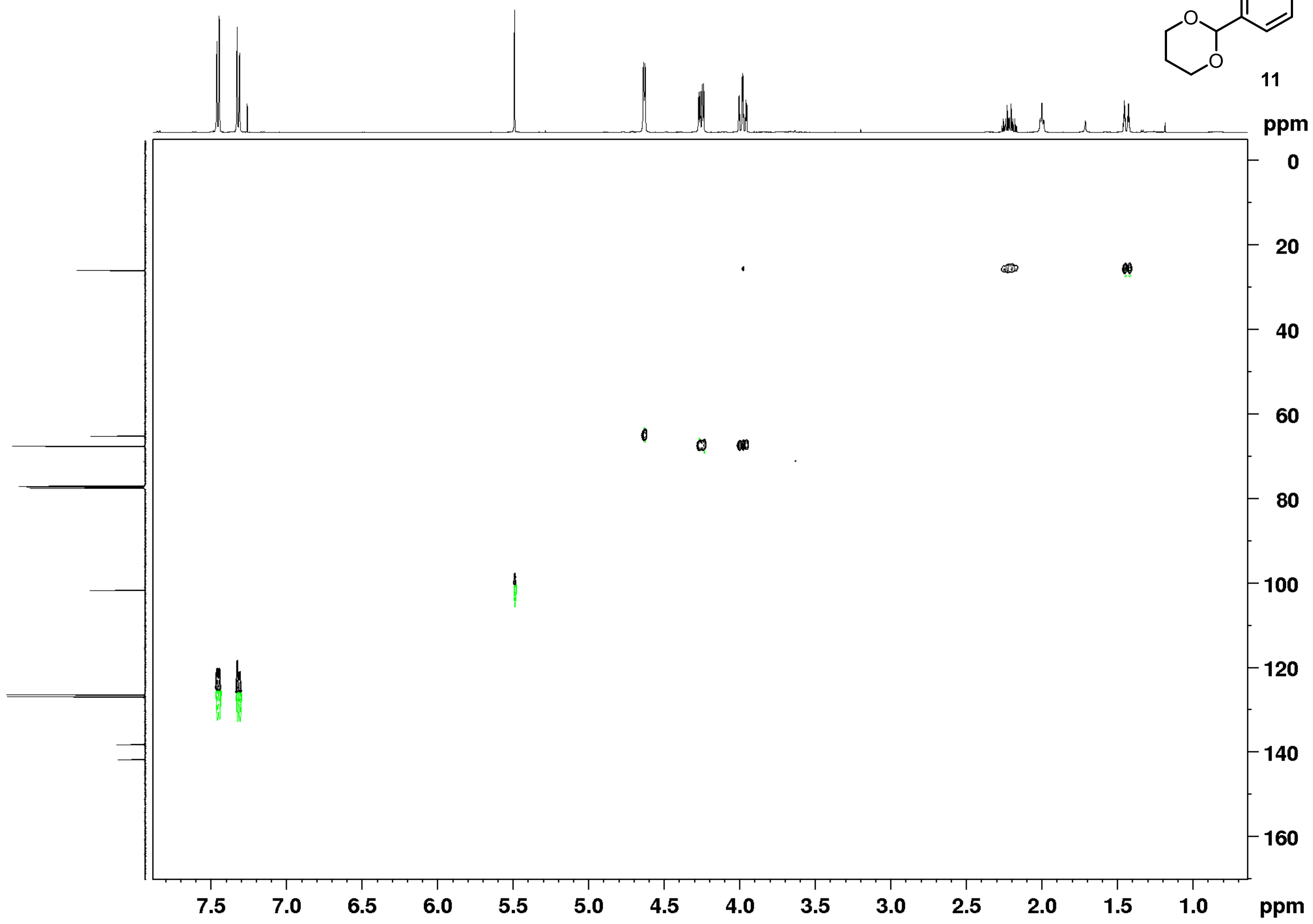

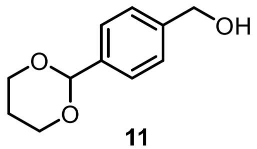

11

HRMS

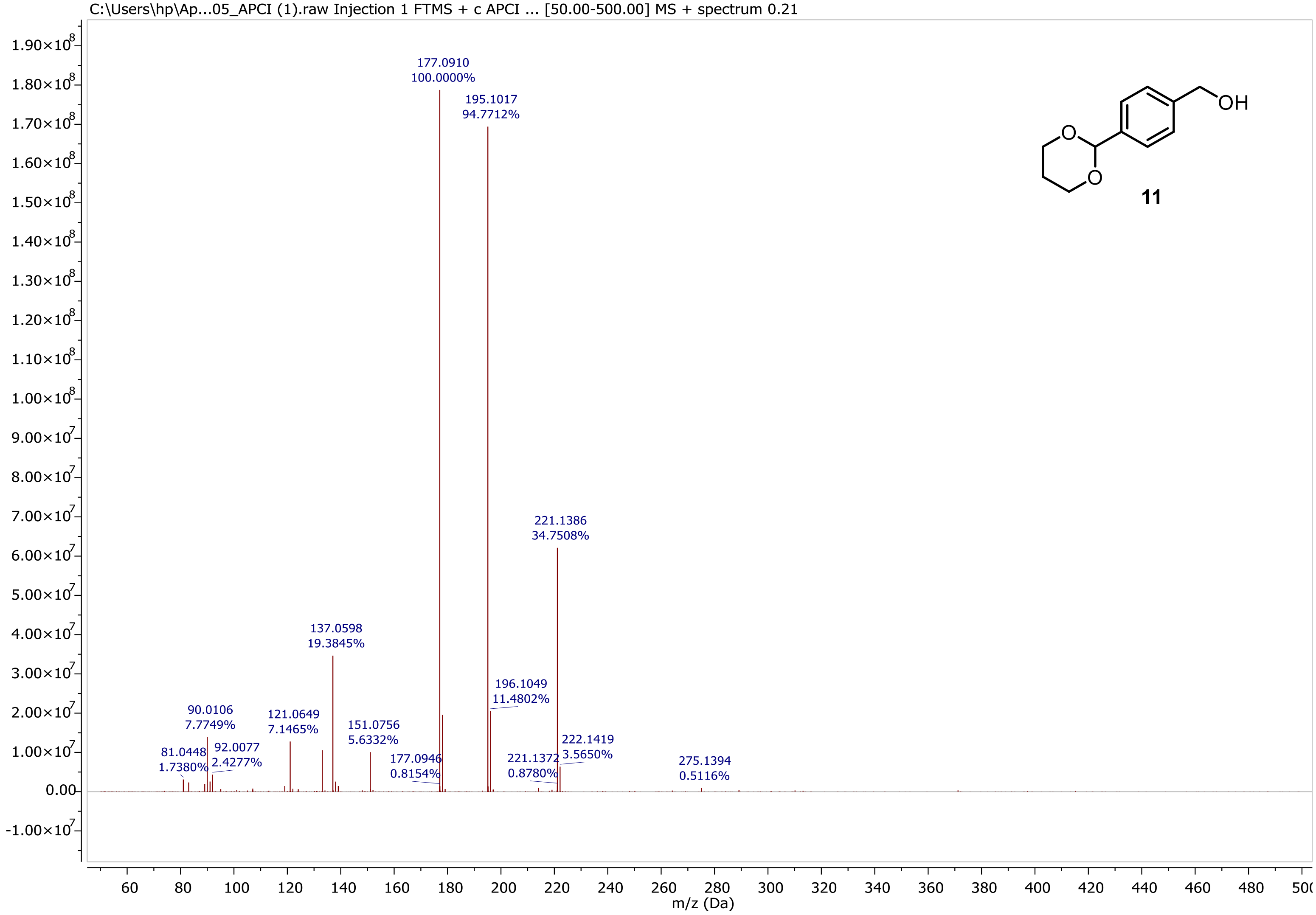

C:\Users\hp\Ap...05_APCI (1).raw Injection 1 FTMS + c APCI ... [50.00-500.00] MS + spectrum 0.21
177.0910
100.0000%
195.1017
94.7712%
221.1386
34.7508%
137.0598
19.3845%
196.1049
11.4802%
90.0106
7.7749%
121.0649
7.1465%
151.0756
5.6332%
81.0448
1.7380%
92.0077
2.4277%
177.0946
0.8154%
221.1372
0.8780%
222.1419
3.5650%
275.1394
0.5116%
m/z (Da)
OH
O
O
11

IR

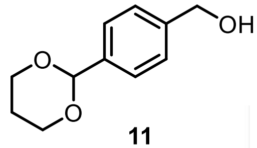

11

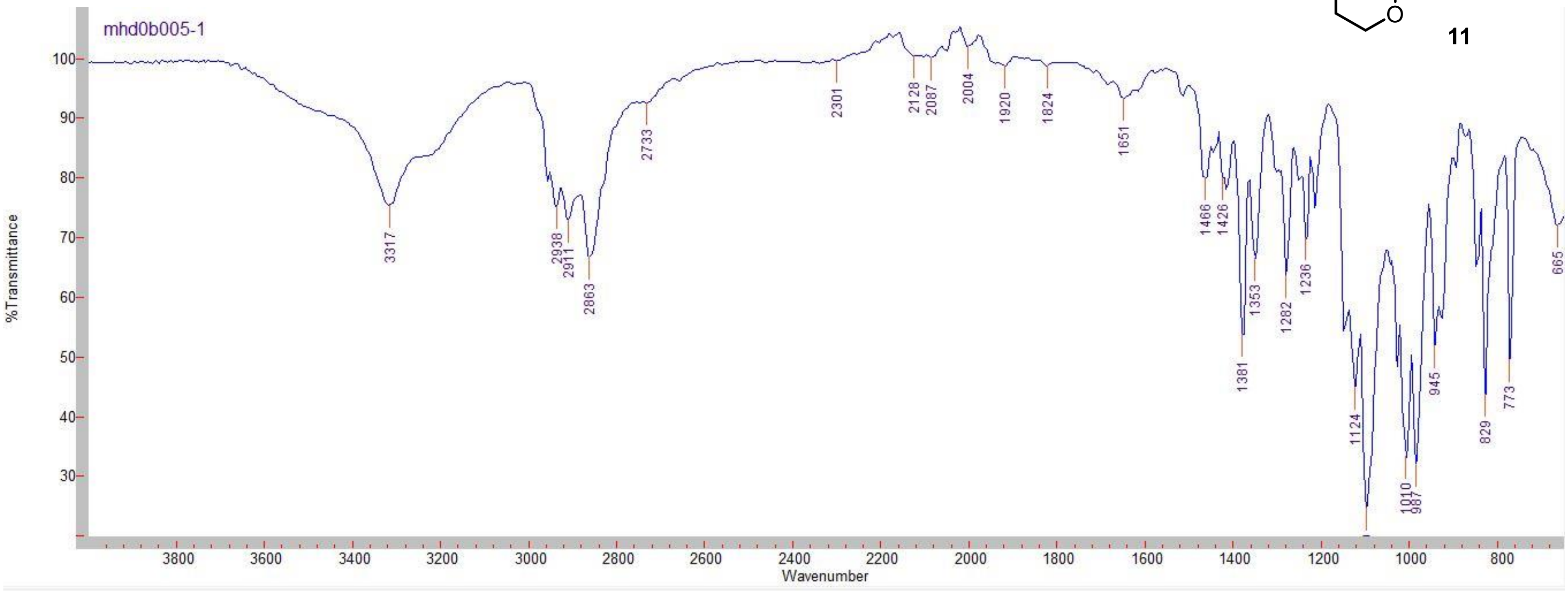
mhd0b005-1
%Transmittance
Wavenumber
3317
2938
2911
2863
2733
2301
2128
2087
2004
1920
1824
1651
1466
1426
1381
1353
1282
1236
1124
1010
987
945
829
773
665

[Cu(IMes)Cl] (7a)

^{1}H-NMR

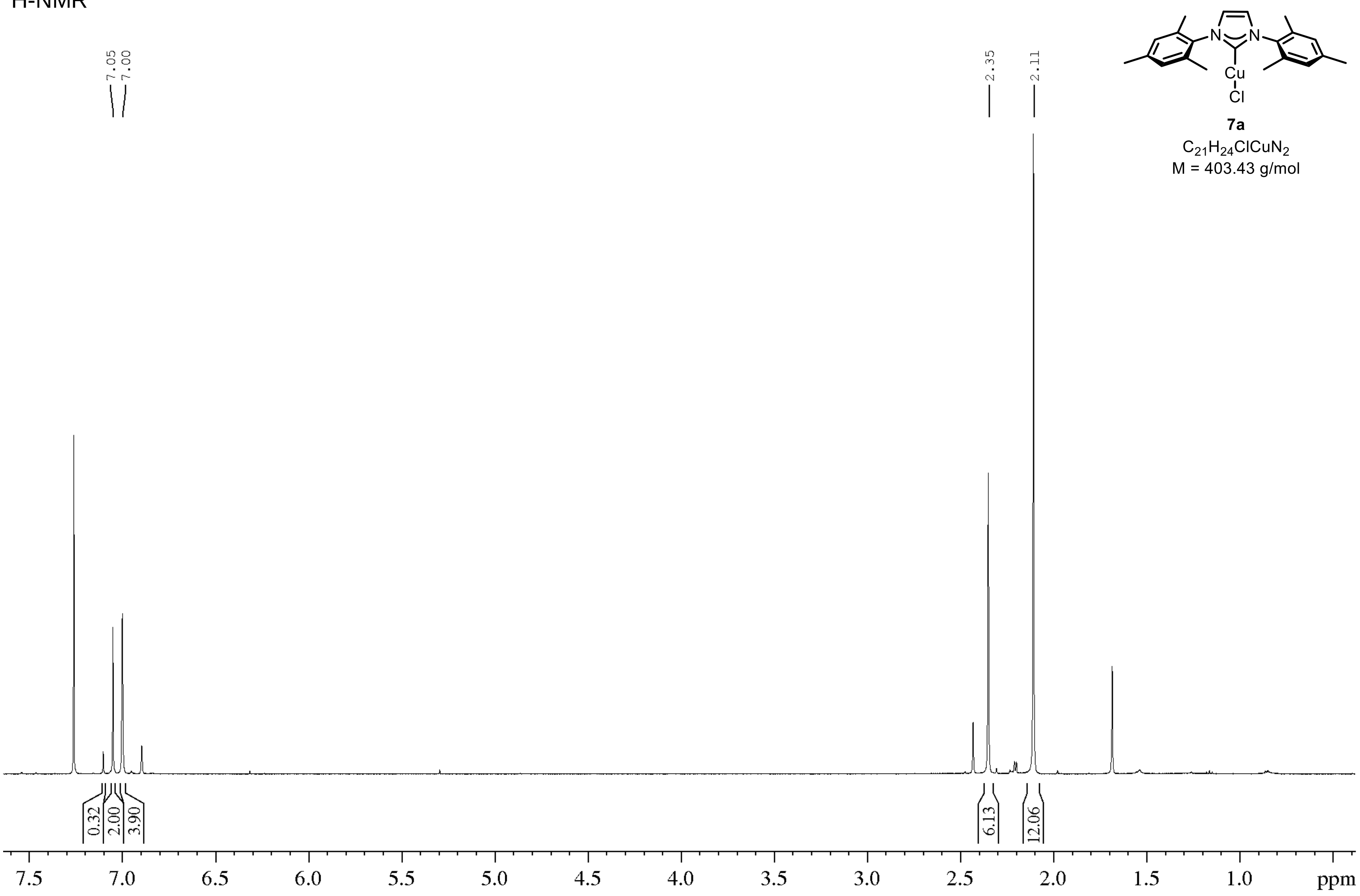

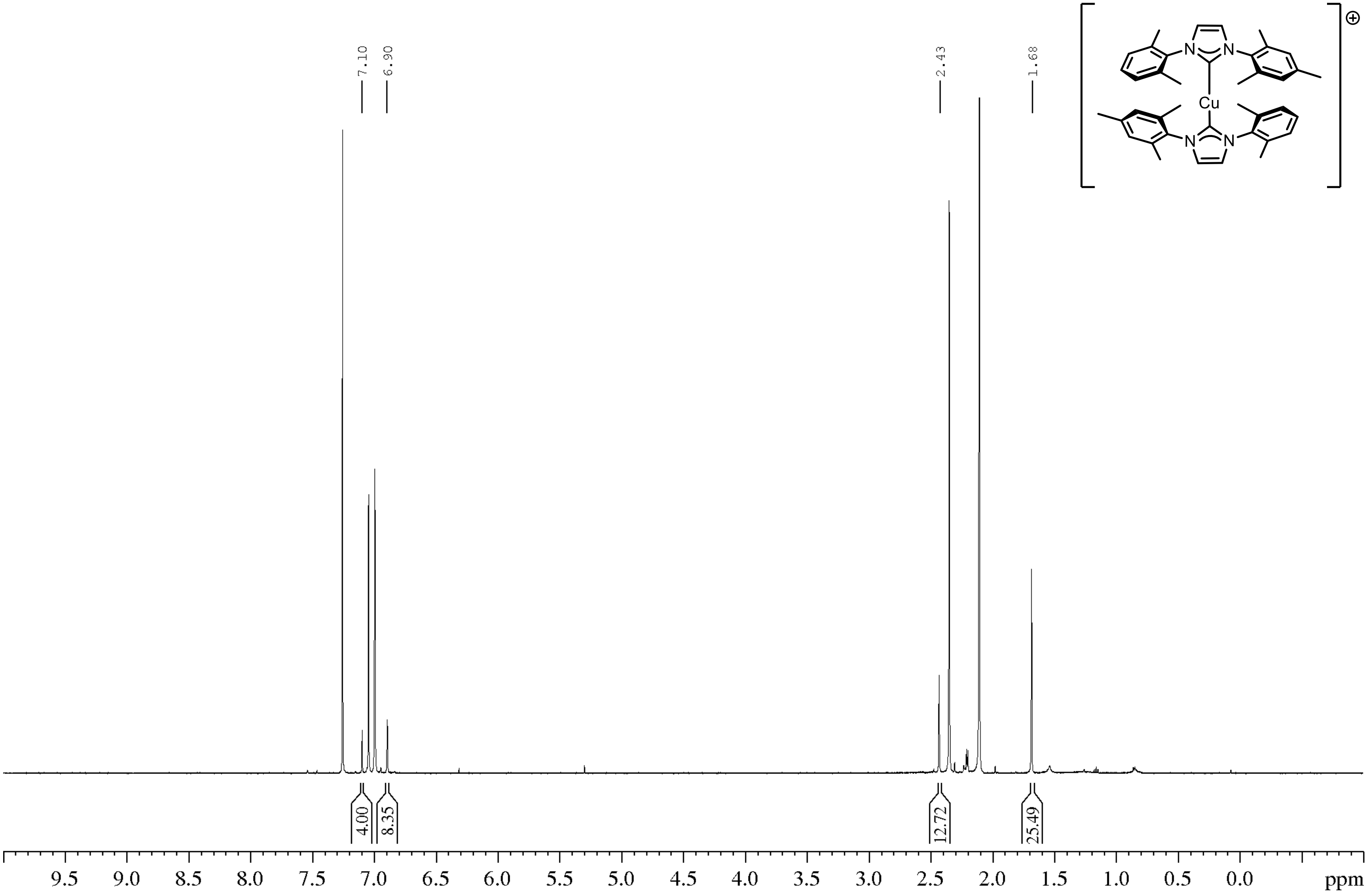
7.10
6.90
2.43
1.68
4.00
8.35
12.72
25.49
9.5
9.0
8.5
8.0
7.5
7.0
6.5
6.0
5.5
5.0
4.5
4.0
3.5
3.0
2.5
2.0
1.5
1.0
0.5
0.0
ppm
N
N
Cu
N
N

[Cu(SIMes)Cl] (7b)

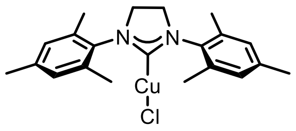

7b

$C_{21}H_{26}ClCuN_2$

M = 405.45 g/mol

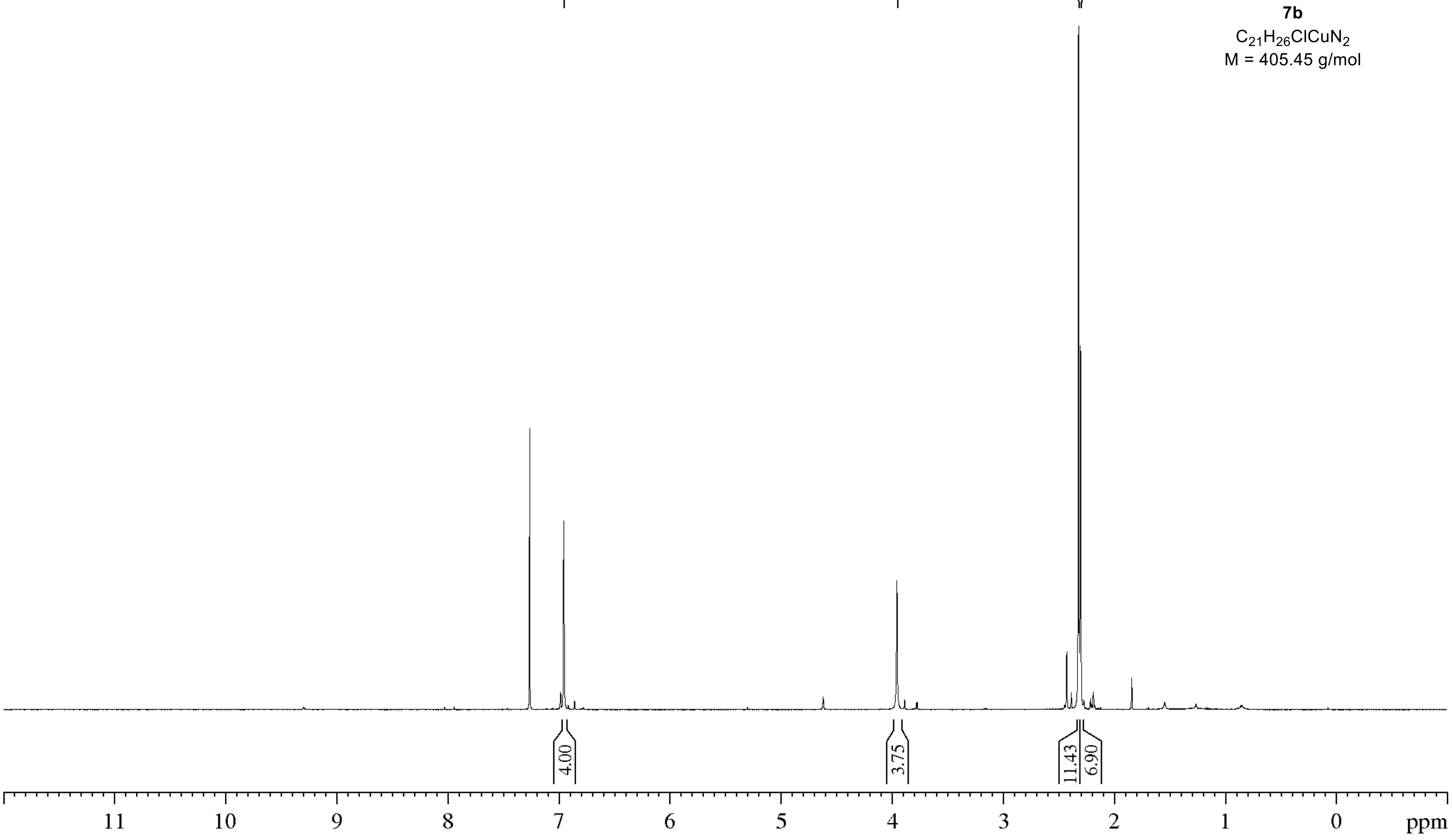

[Cu(IPr)Cl] (7c)

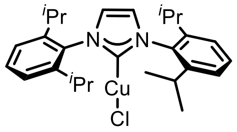

7c

$C_{27}H_{36}ClCuN_2$

M = 487.60 g/mol

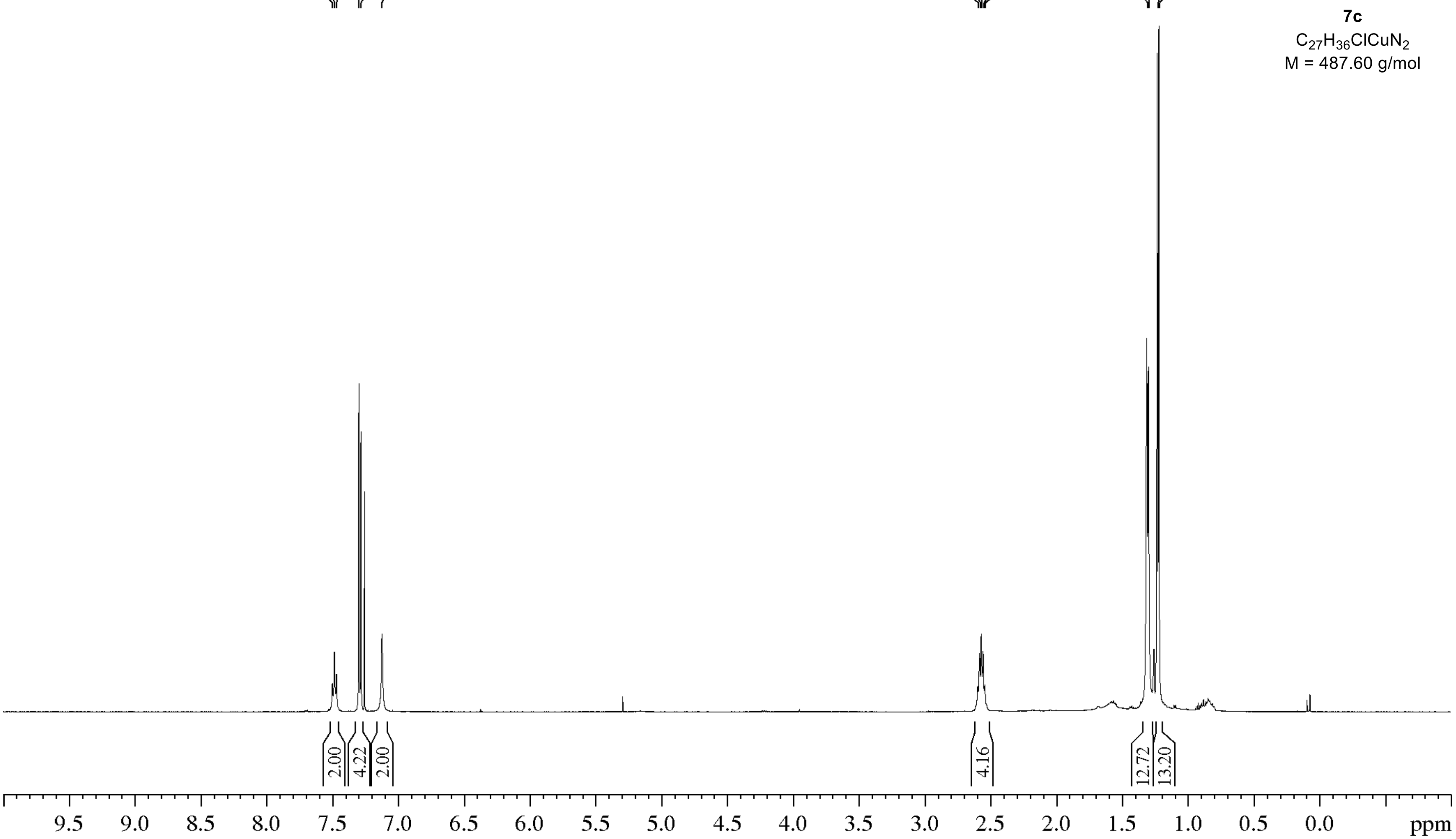

[Cu(SIPr)Cl] (7d)

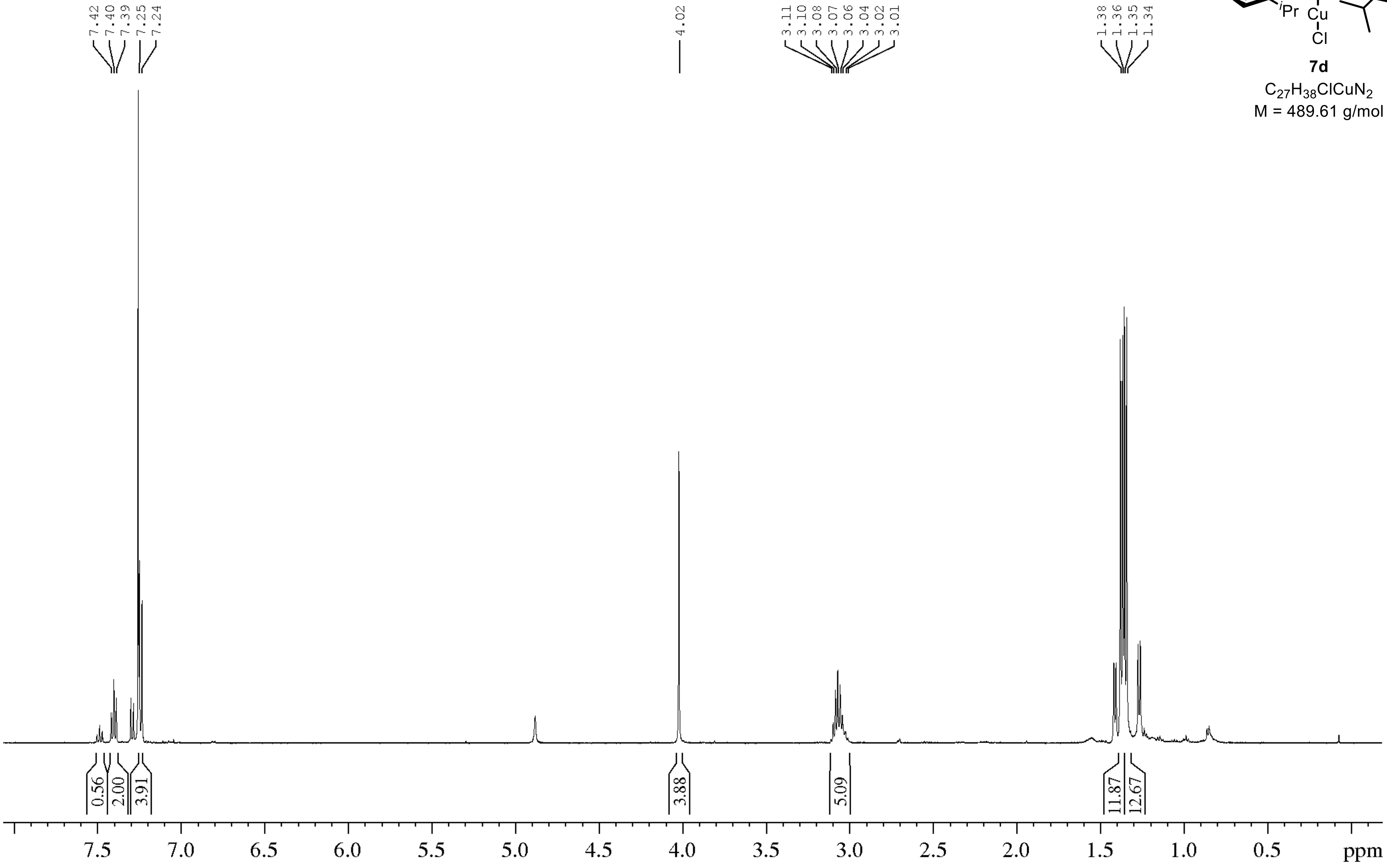

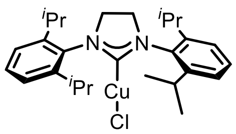

7d

$C_{27}H_{38}ClCuN_2$

M = 489.61 g/mol

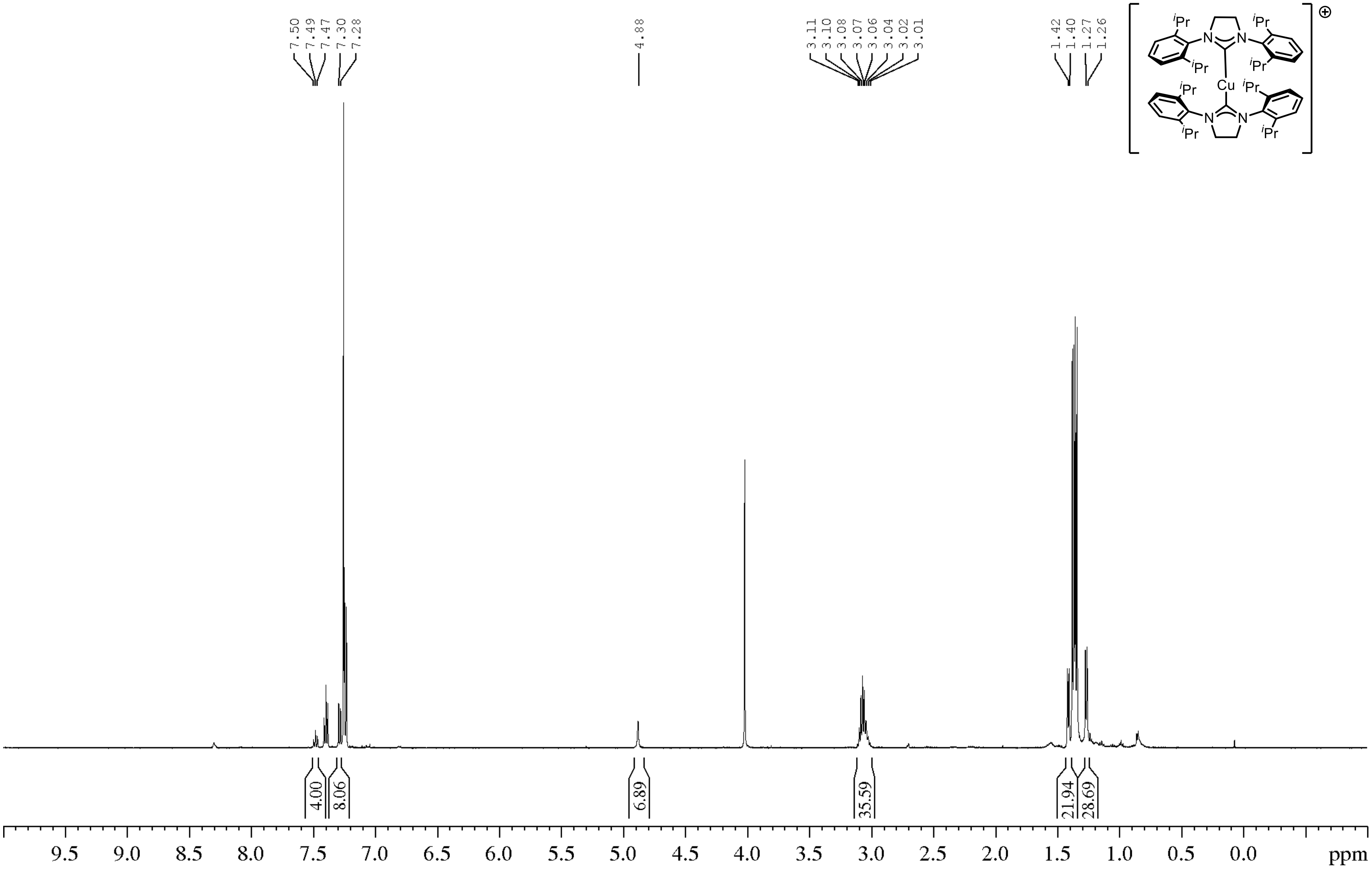
7.50
7.49
7.47
7.30
7.28
4.88
3.11
3.10
3.08
3.07
3.06
3.04
3.02
3.01
1.42
1.40
1.27
1.26
iPr
N
Cu
4.00
8.06
6.89
35.59
21.94
28.69
9.5 9.0 8.5 8.0 7.5 7.0 6.5 6.0 5.5 5.0 4.5 4.0 3.5 3.0 2.5 2.0 1.5 1.0 0.5 0.0
ppm
